# RPL6 Interacts with HMGCS1 to Stabilize HIF‐1α by Promoting Cholesterol Production in Hepatocellular Carcinoma

**DOI:** 10.1002/advs.202501373

**Published:** 2025-07-12

**Authors:** Minli Yang, Shengtao Cheng, Huiying Gu, Shan Zhong, Dapeng Zhang, Xin He, Weixian Chen, Haijun Deng, Jihua Ren, Peng Chen, Ming Tan, Hui Zhang, Fan Li, Zhenzhen Zhang, Yuan Hu, Juan Chen

**Affiliations:** ^1^ Department of Infectious Diseases Key Laboratory of Molecular Biology for Infectious Diseases (Ministry of Education) Institute for Viral Hepatitis The Second Affiliated Hospital Chongqing Medical University Chongqing 400010 China; ^2^ Department of Clinical Laboratory Bishan Hospital of Chongqing Medical University Chongqing 402760 China; ^3^ State Key Laboratory of Ultrasound in Medicine and Engineering College of Biomedical Engineering Chongqing Medical University Chongqing 400016 China; ^4^ Department of Endocrine and Breast Surgery The First Affiliated Hospital of Chongqing Medical University Chongqing 400016 China; ^5^ Department of Infectious Disease Children's Hospital of Chongqing Medical University National Clinical Research Center for Child Health and Disorders Ministry of Education Key Laboratory of Child Development and Disorders Chongqing Key Laboratory of Child Rare Diseases in Infection and Immunity Chongqing 401122 China

**Keywords:** cholesterol, hepatocellular carcinoma, HMGCS1, metastasis, RPL6

## Abstract

The identification of predictive markers to determine the premetastatic phase before metastasis is critical for developing effective strategies for early detection and prevention. By applying the dynamic network biomarker (DNB) approach to analyze time‐series transcriptomic data from a pulmonary metastasis HCC mouse model, it is revealed that the premetastatic phase occurred during the fourth week after implantation. A total of 142 DNB genes are identified as functionally important biomarkers for HCC metastasis, among which 60S ribosomal protein L6 (RPL6) is a core DNB member. RPL6 is significantly upregulated in HCC tissues with extrahepatic metastasis and is strongly correlated with poor prognosis in HCC patients. RPL6 promotes the invasion and metastasis of HCC cells, both in vitro and in vivo. Mechanistically, RPL6 directly binds to the HMGCS1 mRNA 3′UTR, a rate‐limiting enzyme in cholesterol biosynthesis, thus increasing HMGCS1 mRNA stability and protein expression and subsequently elevating intracellular cholesterol level. Elevated cholesterol inhibits the ubiquitin‐dependent degradation of HIF‐1α, which further results in activation of HIF‐1α signaling pathway. Together, this study provides new insights into the dynamic transcriptome profiles of HCC pulmonary metastasis and establishes an important role for the RPL6‐HMGCS1‐HIF‐1α axis in HCC metastasis, suggesting potential prognostic biomarkers and therapeutic targets in HCC.

## Graphical abstract

The study found that RPL6 is frequently upregulated in HCC tissues with extrahepatic metastasis. Elevated RPL6 bound to and increased the stability of HMGCS1 mRNA, thereby enhancing intracellular cholesterol levels. Subsequently, increased cholesterol promotes cancer metastasis in HCC by stabilizing HIF‐1α protein. These findings provide insights into the crosstalk between cholesterol metabolism and hypoxia during HCC metastasis initiation and a promising therapeutic target for the treatment of HCC.

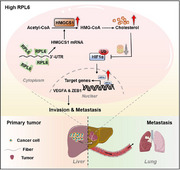

## 1 Introduction

Hepatocellular carcinoma (HCC) is a highly prevalent malignancy worldwide, ranking as the third leading cause of cancer‐related deaths.^[^ ^1^, ^2^ ^]^ Metastasis, a pivotal characteristic of HCC, is responsible for treatment failure and high mortality rates.^[^ ^3^ ^]^ HCC is known for both intrahepatic and extrahepatic metastases, with the lungs being the most common site for extrahepatic dissemination.^[^ ^4^ ^]^ The limited availability of treatments and the unfavorable prognosis underscore the necessity for a deeper investigation into the mechanisms underlying HCC metastasis to ultimately improve patient outcomes.

Although numerous dysregulated or dysfunctional oncogenes and tumor suppressor genes have been reported to be involved in the metastasis process,^[^ ^5^ ^]^ identifying dynamic markers capable of predicting the premetastatic phase and guiding interventions remains a significant challenge. Dynamic disease observation models and analysis methodologies have paved the way for exploring dynamic biomarkers, aiming for the early detection and management of premetastatic HCC.^[^ ^6^, ^7^ ^]^ In this context, we developed a pulmonary metastasis HCC mouse model and examined the dynamic sequential changes in tumor samples. Utilizing the dynamic network biomarker (DNB) approach,^[^ ^8^, ^9^ ^]^ we identified the fourth week after implantation as the tipping point, representing a critical state preceding metastasis initiation. Concurrently, we successfully identified a group of DNB genes with potential early warning value, in which 60S ribosomal protein L6 (RPL6) was identified as a prime DNB member involved in metastasis initiation.

Ribosomal proteins (RPs), which are traditionally recognized as essential components of the ribosome, play key roles in ribosome biogenesis and protein translation.^[^ ^10^ ^]^ Recent studies have shown that RPs have additional extraribosomal functions, independent of protein biosynthesis, in the regulation of diverse pathological processes, especially tumorigenesis and cell transformation.^[^ ^11^ ^]^ Many RPs, including RPS7, RPS15, RPS20, RPL15, RPL23, RPL27a, RPL34 and RPL39L, are overexpressed in various human cancers.^[^ ^12^, ^13^ ^]^ These overexpressed RPs contribute to cancer cell proliferation, cell apoptosis, cell cycle progression, and DNA repair.^[^ ^14^, ^15^, ^16^, ^17^ ^]^ In breast cancer, RPL15 is enriched in circulating tumor cells, and facilitates metastatic growth in various organs.^[^ ^18^ ^]^ RPS27a has been shown to promote cell proliferation and inhibit cell apoptosis in lung adenocarcinoma cells by interacting with RPL11, disrupting the RPL11‐MDM2 complex, and preventing p53 ubiquitination and degradation.^[^ ^19^ ^]^ RPS15 has been shown to interact with IGF2BP1 to promote the proliferation and metastasis of human esophageal squamous cell carcinoma cells by recognizing m6A modification.^[^ ^20^ ^]^ In summary, the above researches strongly indicate that RPs are tightly associated with tumorigenesis in various cancers. Consistently, RPL6, a component of the large ribosomal subunit, is involved in various nonribosomal activities, such as the DNA damage response, apoptosis regulation, cell cycle control, and defense against microbial infections.^[^ ^21^, ^22^, ^23^, ^24^ ^]^ However, the exploration of the functional role of RPL6 in cancer is limited.^[^ ^25^, ^26^ ^]^ And the important role and molecular mechanism of RPL6 in HCC metastasis remain unexplored.

In this study, we reported that RPL6 was frequently upregulated in a subset of HCC tissues with extrahepatic metastasis, and was correlated with poor prognosis in HCC patients. RPL6 bound to and increased the stability of HMGCS1 mRNA, the transcript of a key enzyme in cholesterol synthesis, thereby enhancing intracellular cholesterol levels. We further identified HIF‐1α as the key downstream target of cholesterol and revealed the mechanism by which cholesterol stabilizes the HIF‐1α protein to promote HCC metastasis. Taken together, our data demonstrate that RPL6, as a core DNB member, is involved in an extraribosomal mechanism, providing insights into the crosstalk between cholesterol metabolism and hypoxia during HCC metastasis initiation and a promising therapeutic target for the treatment of HCC.

## 2 Results

### 2.1 Identifying RPL6, an RNA Binding Protein, as a Key Factor in HCC Metastasis

**Figure 1:**
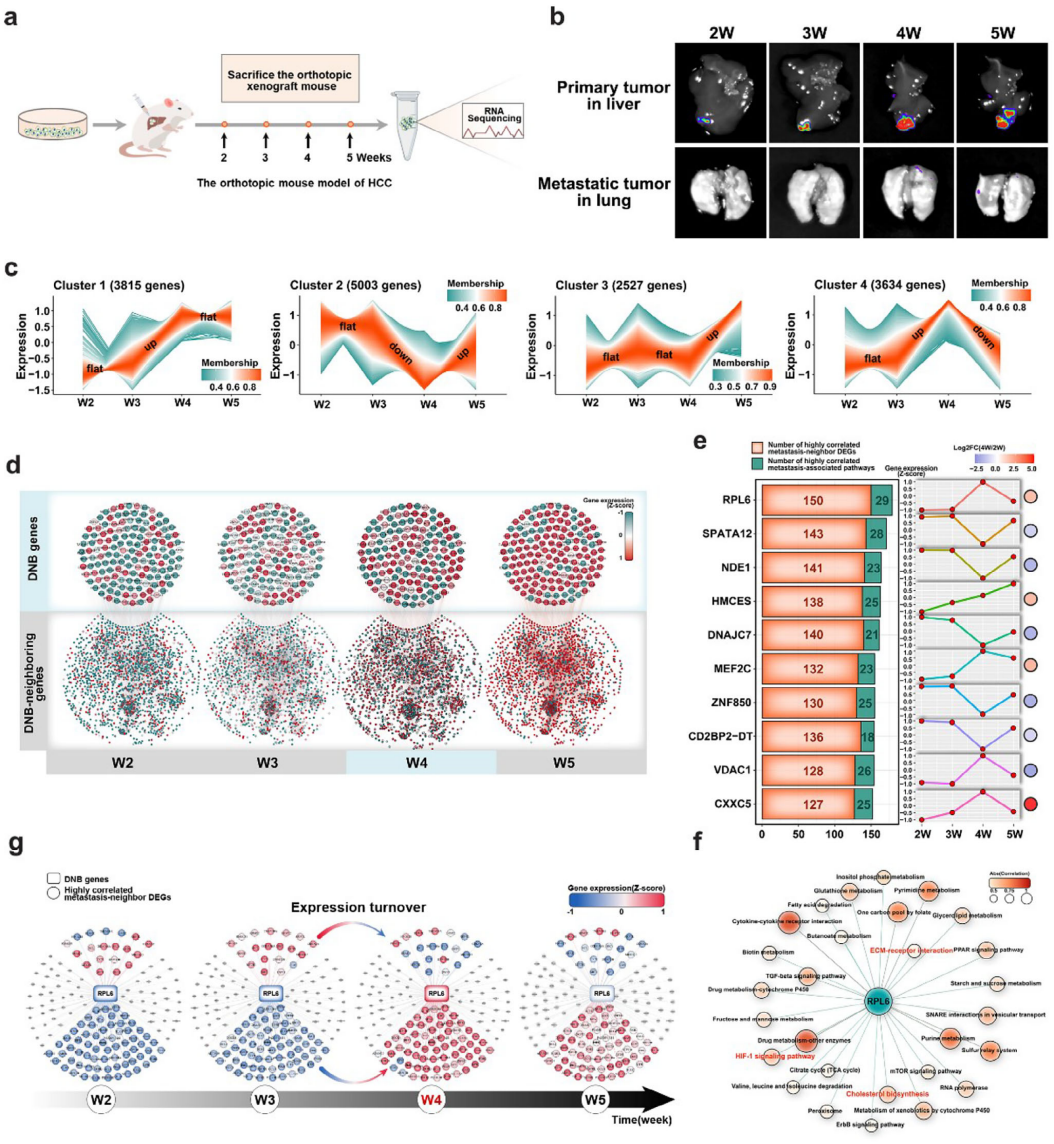
The identification of RPL6 as a crucial gene closely linked to HCC metastasis. a) A flow chart illustrating the process for creating an orthotopic liver cancer mouse model with pulmonary metastasis using MHCC97H‐GFP cells. b) The representative bioluminescence images showing liver tumors and pulmonary metastases at four time points in the orthotopic xenograft MHCC97H mouse model. c) Temporal trend clustering of gene expression captured by R package Tcseq. d) Cytoscape visualization of the expression profiles and PPI interaction networks between the DNB genes and DNB‐Neighbor DEGs at four time points. A color gradient from green to red is applied to represent gene expression levels, ranging from low to high. e) The analysis includes correlation studies between DNB genes and metastasis‐specific DNB‐Neighbor DEGs, as well as GSVA to assess the correlation of DNB genes with metastasis‐associated KEGG pathways (left). The temporal trends in the expression of the corresponding DNB genes are presented (right). The top 10 most significant DNB genes are highlighted. f) Global view of the expression patterns associated with RPL6‐mediated metastasis pathways. g) Dynamic changes of expression levels of RPL6 and its 84 metastasis‐neighbor DEGs at four time points. DNB, dynamic network biomarker.

To systematically explore the pivotal phases and important genes associated with HCC metastasis, we established a pulmonary metastasis HCC mouse model and the gene expression patterns HCC metastasis were analyzed comprehensively. In this model, GFP‐tagged MHCC97H cells with high metastatic potential were orthotopically transplanted into the livers of nude mice (**Figure** **1**a). Continuous observation revealed that hepatic tumors in orthotopic xenograft MHCC97H‐GFP mice grew gradually from the second week to the fifth week after orthotopic implantation, whereas distant pulmonary metastasis initially occurred at the fourth week (Figure 1b; Figure S1a, Supporting Information).

Based on our RNA sequencing (RNA‐seq) data of HCC tissues from orthotopic xenograft mice at four time points, dynamic genetic perturbation screens were performed by utilizing the R package Tcseq. Large‐scale gene perturbation (14 979/40 283, 38.7%), which exhibits four distinct non‐linear expression patterns, was captured throughout the metastasis process (Figure 1c). The R package BioTIP (which encapsulates the DNB algorithm) was then employed to detect the DNB genes based on perturbed gene sets. We obtained the 142 DNB members and corresponding DNB‐neighboring genes whose dynamics signal the state transition during HCC metastasis (Figure 1d). Those DNB genes functioning were associated with ECM‐receptor interaction, focal adhesion, regulation of actin cytoskeleton, and tight junction (Figure S1b, Supporting Information). Importantly, a total of 39 genes (39/142, 27.46%) were directly involved in cancer metastasis‐related pathways (Figure S1c, Supporting Information), of which ribosomal protein L6 (RPL6) was highly correlated with the greatest number of metastasis‐associated neighboring genes and pathways (Figure 1e; Figure S1d, Supporting Information). In particular, RPL6 was directly involved in the pathways associated with metastasis initiation, such as regulation of the ECM‐receptor interaction, cholesterol biosynthesis, and the HIF‐1 signaling pathway (Figure 1f). Thus, we analyzed the dynamics of RPL6 and its metastasis‐associated neighboring genes throughout metastatic progression at the network level. The expression levels of RPL6 and its 84 (84/150, 56%) metastasis‐associated neighboring genes were all significantly reversed before and after the tipping point of metastasis initiation (Figure 1g). Furthermore, we analyzed the dynamic activity of the related KEGG pathways involving RPL6 and its neighboring genes in different dynamic patterns. We found that metastasis‐related pathways, such as the HIF‐1 signaling pathway, were significantly enriched (Figure S1e, Supporting Information). These data suggest that RPL6 plays an important role in HCC metastasis. Therefore, we focused on the pivotal roles and underlying molecular mechanism of RPL6 in driving HCC metastasis.

### 2.2 RPL6 Is Upregulated in Metastatic Tumors and Correlated with Poor Prognosis of HCC Patients

**Figure 2:**
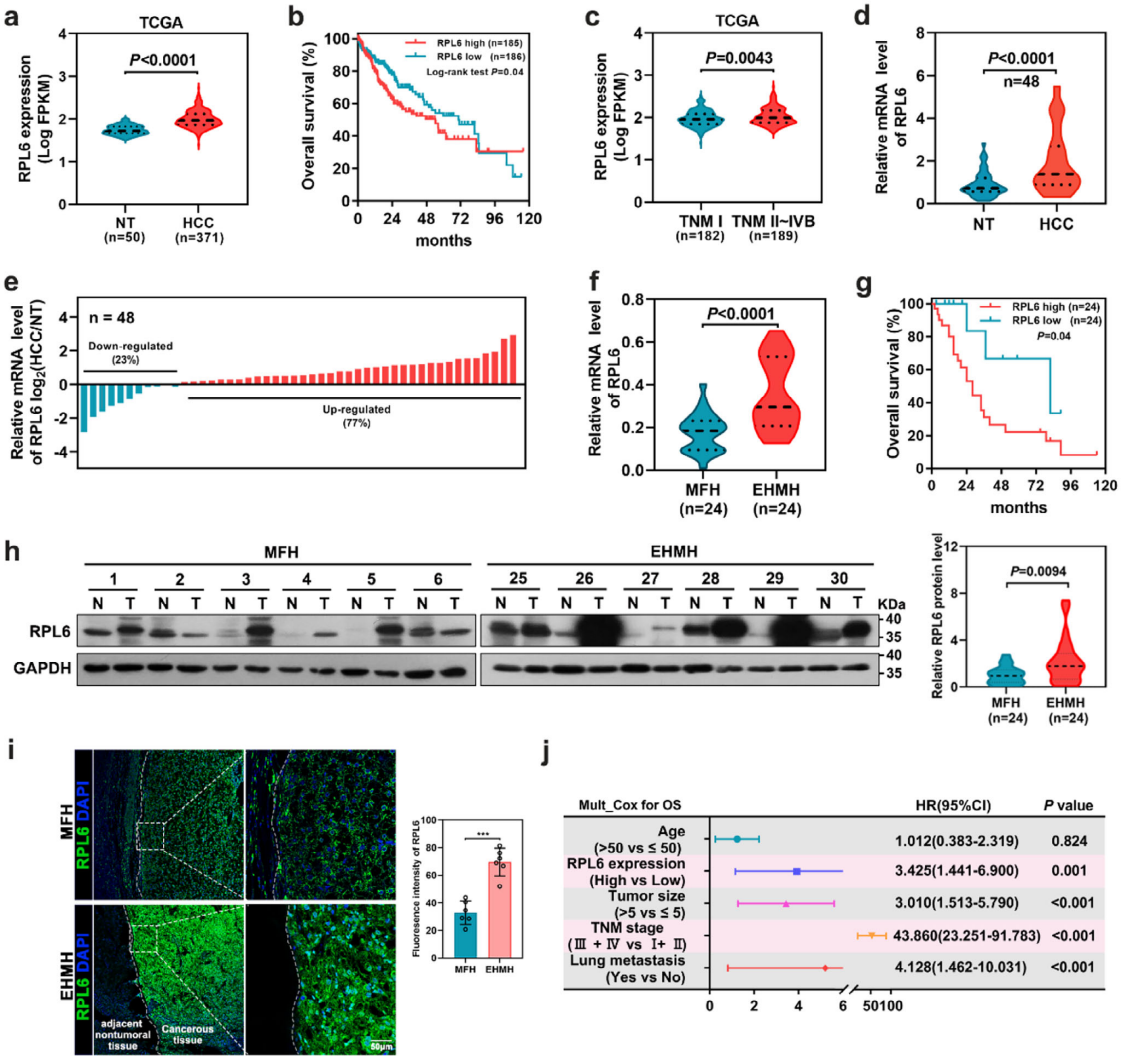
RPL6 is up‐regulated and correlated with HCC patient prognosis. a) The mRNA level of RPL6 in HCC tissues (n = 371) and non‐tumor liver tissues (n = 50) based on the TCGA‐LIHC database. b) The Kaplan‐Meier plot illustrates overall survival within the TCGA‐LIHC cohort, stratified based on the median expression level of RPL6. c) The mRNA level of RPL6 in HCC metastasis group (TNM II to IVB, n = 189) and HCC non‐metastasis group (TNM I, n = 182) based on the TCGA‐LIHC database. d,e, The mRNA level of RPL6 was analyzed by qRT‐PCR in 48 paired HCC tissues and their corresponding adjacent tissues. f) The mRNA level of RPL6 in primary HCC tissues with EHMH and MFH was assessed by qRT‐PCR analysis. g) The Kaplan‐Meier plots illustrating the correlation between the RPL6 mRNA level and overall survival in 48 HCC patients. h) The protein level of RPL6 in primary HCC tissues with EHMH (n = 24) and MFH (n = 24) was assessed by western blot analysis. Band intensity was quantified using image J. i) The representative images demonstrated that RPL6 expression in primary HCC of the EHMH and MFH groups was assessed using multiple immunofluorescence (mIFC) staining. Scar bar, 50 µm. j) The forest plot displayed the results of the multivariate analysis of factors associated with overall survival (OS). Data are shown as mean ± SD. Statistical significance was determined by two‐tailed paired Student's t‐test (d,f,h), two‐tailed unpaired Student's t‐test or log‐rank test (a,c). Multivariate Cox regression analysis was used to assess HRs and the associated 95% CIs (j). *P < 0.05; **P < 0.01; ***P < 0.001. NT, non‐tumor; HCC, hepatocellular carcinoma; TNM, Tumor‐Node‐Metastasis classification; MFH, HCC tissues with metastasis‐free; EHMH, HCC tissues with extrahepatic metastasis; N, non‐tumor; T, tumor.

To determine the clinical relevance of RPL6 in HCC, high expression of RPL6 in HCC samples was detected in the Cancer Genome Atlas‐Liver Hepatocellular Carcinoma (TCGA‐LIHC) dataset, and found to be strongly associated with poor patient outcome (**Figure** **2**a,b). According to the tumor‐node‐metastasis (TNM) staging system, 371 patients with HCC in TCGA‐LIHC were divided into two groups: 189 patients with HCC with intra‐ and/or extrahepatic metastases (TNM II to IVB group) and 182 patients with HCC without metastases (TNM I group). RPL6 was more highly expressed in the metastasis group compared with the metastasis‐free group (Figure 2c).

To elucidate the clinical significance of RPL6 in HCC, we next examined the expression level of RPL6 in 48 matched pairs of primary HCC tissues and their corresponding non‐tumoral liver samples. There were 24 pairs of HCC tissues with extrahepatic metastasis (EHMH) and another 24 pairs of HCC tissues with metastasis‐free (MFH). We found that the mRNA level of RPL6 was significantly increased in tumor tissues in comparison to their matched adjacent normal tissues (Figure 2d). In 48 pairs of tissue samples, RPL6 expression was markedly upregulated in 77% (37/48) of the HCC samples (Figure 2e), and higher in the EHMH group compared with MFH group (Figure 2f). Moreover, there was a negative correlation between RPL6 expression in HCC tissues and the overall survival rates of the respective HCC patients (Figure 2g). Consistently, the protein expression level of RPL6 was significantly raised in the EHMH compared to the MFH group (Figure 2h; Figure S2a, Supporting Information), which was further confirmed by multiple immunofluorescence (mIFC) and IHC assays (Figure 2i; Figure S2b, Supporting Information). Correlation analysis also revealed that higher RPL6 expression was strongly associated with aggressive tumor traits. This includes increased vascular invasion, lung metastasis, and advanced TNM stage. However, no significant correlation was detected between RPL6 expression and factors such as gender, age, AFP level, liver cirrhosis, or tumor encapsulation (Table S1, Supporting Information). Furthermore, multivariate Cox regression analysis indicated that high expression of RPL6 has the potential to be an independent predictor of survival outcomes of HCC patients (Figure 2j). Collectively, these results suggest that RPL6 might be a useful prognostic biomarker in HCC.

### 2.3 RPL6 Promotes HCC Growth and Metastasis In Vitro and In Vivo

**Figure 3:**
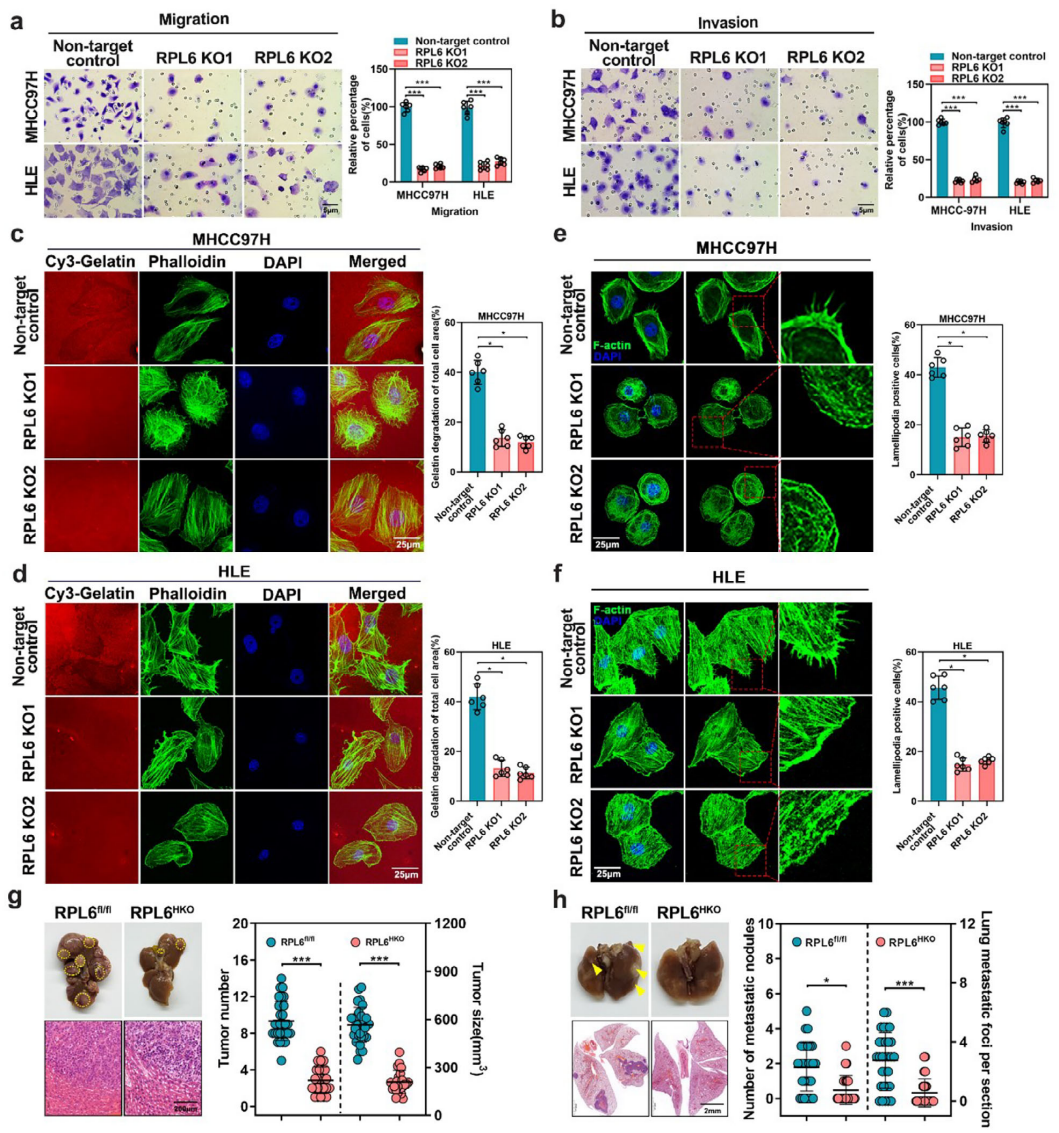
Knockout of RPL6 inhibits metastasis of HCC cells in vitro and in vivo. a,b, Migratory (a) and invasive (b) properties of MHCC97H and HLE HCC cells with RPL6 knockout compared to non‐target control. The relative numbers of migrated or invaded cells were expressed as percentage of the control group. Cell counts were counted from 6 images. c,d, The effect of RPL6 knockout on invadopodia function in MHCC97H (c) and HLE (d) cells was evaluated using a gelatin degradation assay. Cells were seeded on Cy3‐gelatin matrix (red) and cultured for 48 h. After fixing the cells and staining with FITC‐phalloidin (green) and DAPI (blue), confocal images were captures and representative images are displayed. Degraded areas were quantified using Image J from at least six fields. Scar bar, 25 µm. e,f, The effect of RPL6 knockout on lamellipodia function was assessed by F‐actin staining in MHCC97H (e) and HLE (f) cells. Representative images were presented, and the percentage of cells exhibiting lamellipodia formation was counted from at least six fields. Scar bar: 25 µm. g,h, A DEN/CCl4‐induced HCC model was established in hepatocyte‐specific RPL6 knockout mice (RPL6^HKO^) and corresponding control mice (RPL6^fl/fl^). Liver and lung tissues collected at 10 months post DEN treatment were analyzed, n = 25‐30 per group. g) Representative macroscopic images and H&E staining of tumor‐bearing livers in hepatocyte‐specific RPL6 knockout mice (RPL6^HKO^, n = 25) and corresponding control mice (RPL6^fl/fl^, n = 30). Tumor number and tumor size were examined. Scar bar, 200 µm. h) Representative macroscopic images and H&E staining of tumor‐metastasis lung in RPL6^HKO^ and RPL6^fl/fl^ mice. The number of metastatic nodules and foci were counted. Scar bar, 2 mm. Data are shown as mean ± SD of at least three independent experiments (a‐f). Statistical significance was determined by two‐tailed unpaired Student's t‐test. *P < 0.05; ***P < 0.001.

To explore the biological function of RPL6 in HCC, we established stable RPL6 knockout cell models in HCC cell lines (MHCC97H and HLE) with high metastatic potential by utilizing the CRISPR/Cas9 system (Figure S3a–c, Supporting Information). CCK‐8 and colony‐formation assays revealed that RPL6 knockout significantly suppressed the proliferation rate and colony‐forming abilities in HCC cells (Figure S3d,e, Supporting Information). Moreover, RPL6 knockout markedly decreased the migratory and invasive abilities of MHCC97H and HLE cells, as evidenced by the results of wound‐healing, transwell migration, and invasion assays (**Figure** **3**a,b; Figure S3f, Supporting Information). Invadopodia are actin‐rich membrane protrusions with a degradation activity of extracellular matrix (ECM) during cell invasion. The function of invadopodia refers to the colocalization and matrix degradation of filamentous actin, as indicated by the localized proteolytic degradation of gelatin. The results revealed that RPL6 knockout reduced the invasive ability of HCC cells, as demonstrated by a decreased area of gelatin degradation per cell (Figure 3c,d). Lamellipodia, the dynamic cell surface extensions responsible for driving cell movement, were also examined. The F‐actin staining showed that lamellipodia formation was notably suppressed in RPL6‐knockout MHCC97H and HLE cells (Figure 3e,f). In contrast, RPL6 overexpression dramatically promoted the cell proliferation rate, colony formation ability and migration and invasion of Huh7 and PLC/PRF/5 cells, which have low metastatic potential (Figure S4a‐e, Supporting Information). Gelatin degradation assay and F‐actin staining also revealed that overexpression of RPL6 significantly increased the abilities of invadopodia and lamellipodia formation in HCC cells (Figure S4f,g, Supporting Information).

To investigate whether RPL6 has an impact on HCC progression in vivo, hepatocyte‐specific RPL6‐knockout (RPL6^HKO^) mice were generated by crossing RPL6^fl/fl^ mice with Alb‐Cre mice (Figure S5a,b, Supporting Information). Then, we constructed a DEN/CCl_4_‐induced hepatocellular carcinoma model by the intraperitoneal injection of DEN or CCl4 into RPL6^fl/fl^ and RPL6^HKO^ mice. Compared with the control mice, RPL6^HKO^ mice exhibited a decreased number of liver tumors, and reduced tumor size (Figure 3g). Moreover, a decreased incidence of lung metastases was observed in the RPL6^HKO^ mice compared with the RPL6^fl/fl^ mice (Figure 3h). Approximately 83.3% (25/30) of the RPL6^fl/fl^ mice had lung metastases, while only 32% (8/25) of the RPL6^HKO^ mice developed lung metastases (Figure S5c, Supporting Information). In addition, we generated an intrahepatic injection mouse model of lung metastasis by injecting RPL6‐overexpressing Huh7 cells into the left lobe of the orthotopic liver of nude mice. Eight weeks after injection, we observed that RPL6 overexpression significantly promoted the number and size of liver tumors, as well as lung metastatic nodules and foci (Figure S6a‐d, Supporting Information). Collectively, these in vitro and in vivo findings suggest that the upregulation of RPL6 expression is an important oncogenic event during HCC progression.

### 2.4 RPL6 Interacts with HMGCS1 mRNA and Increases Its Stability to Promote HCC Metastasis

**Figure 4:**
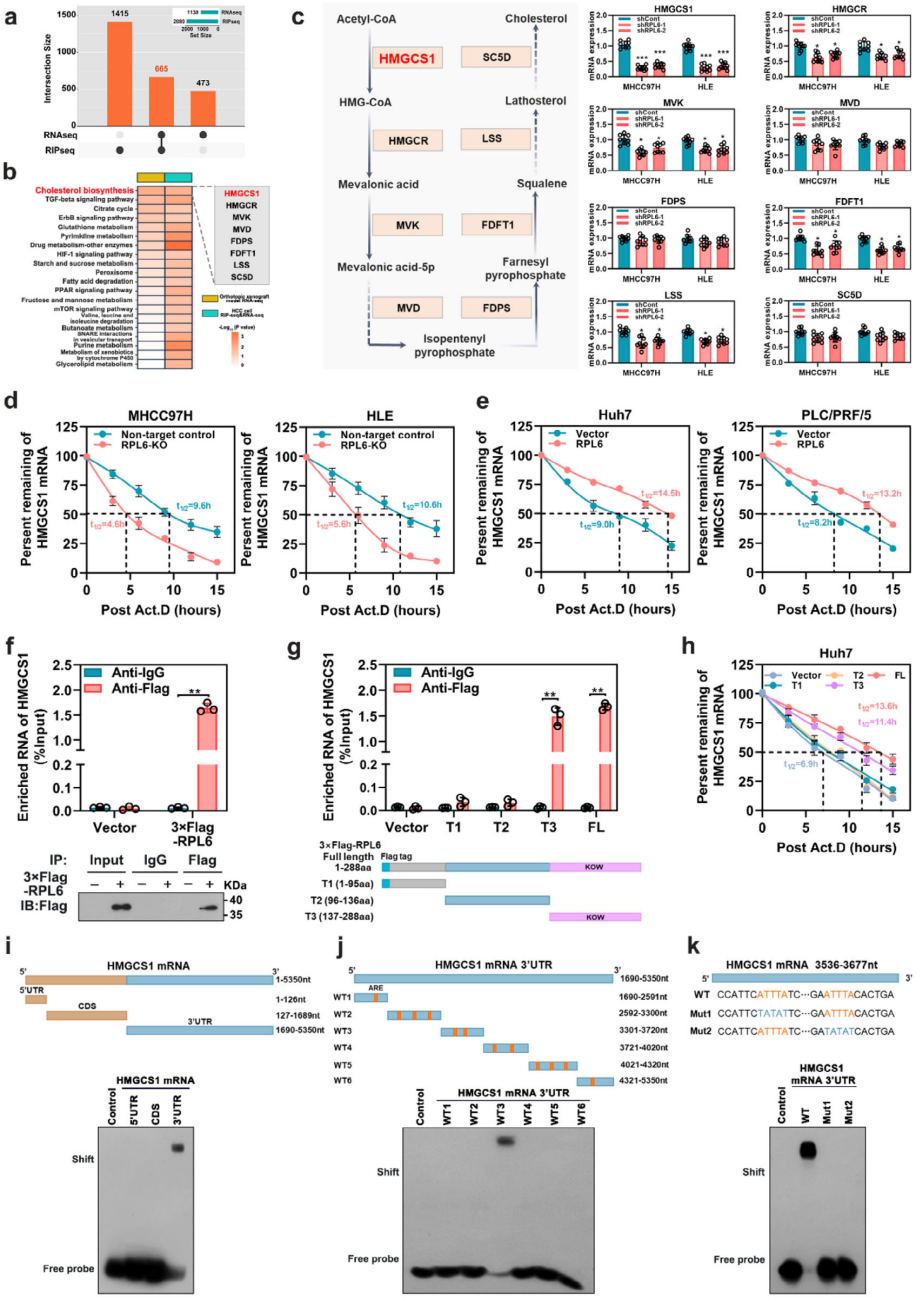
RPL6 interacts with HMGCS1 mRNA in HCC cells. a) An UpSet plot illustrated both the RPL6‐associated mRNAs identified by RIP‐seq and RPL6‐regulated downstream mRNAs determined by RNA‐seq. b) Association analysis of KEGG pathway enrichment was performed between the Orthotopic xenograft model RNA‐seq and HCC cell RIP‐seq and RNA‐seq (left), showing differential expression of key genes in the cholesterol biosynthesis pathway (right). c) Differential expression of key genes in the cholesterol biosynthesis pathway in RPL6‐knockout MHCC97H and HLE cells. d) qRT‐PCR analysis of HMGCS1 mRNA half‐life in RPL6‐knockout MHCC97H and HLE cells after actinomycin D treatment. e) qRT‐PCR analysis of HMGCS1 mRNA half‐life in RPL6‐overexpressing Huh7 and PLC/PRF/5 cells after actinomycin D treatment. f) The enrichment of HMGCS1 mRNA on RPL6 was detected by RIP assay (upper). Western blot was performed to confirm that 3xFlag‐RPL6 immunoprecipitated in the RIP experiments (lower). g) RIP experiments detected the binding between RPL6 truncation mutants and HMGCS1 mRNA (upper). The diagrams illustrate the full length of Flag‐tagged RPL6 (FL, 1–288aa) and its Flag‐tagged truncations, including the N‐terminal domain (T1, 1–95aa), the middle intervening sequence (T2, 95–136aa), and the C‐terminal KOW domain (T3, 136–288aa). h) qRT‐PCR analysis measured the HMGCS1 mRNA half‐life in Huh7 HCC cells overexpressing RPL6 truncation mutants after treatment with actinomycin D. i) An RNA Electrophoretic mobility shift (REMSA) assay was used to determine the interaction between purified RPL6 protein and a biotin‐labeled probe of HMGCS1 truncations (5′UTR, CDS, and 3′UTR). j,k) REMSA assays were conducted to determine the interaction between the RPL6 protein and biotin‐labeled probes of specified truncations of HMGCS1 3′UTR. Data are shown as mean ± SD of at least three independent experiments (c‐h). Statistical significance was determined by two‐tailed unpaired Student's t‐test (c–g) or one‐way analysis of variance (ANOVA) (h). *P < 0.05; **P < 0.01; ***P < 0.001. Act.D, actinomycin D; T, truncation; FL, full length; UTR, untranslated regions; CDS, coding sequences; WT, wild type; Mut, mutation.

As an RNA‐binding protein, RPL6 has been reported to bind RNA through RNA‐binding domains to change the fate or function of the bound RNA.^[^ ^27^ ^]^ To identify direct targets of RPL6, we performed RNA immunoprecipitation sequencing (RIP‐seq) in MHCC97H cells expressing Flag‐RPL6 and identified 2080 RPL6‐associated mRNAs (Figure S7a, Supporting Information). We analyzed RPL6‐regulated genes by using RNA sequencing (RNA‐seq) and identified 1138 differentially expressed genes in response to RPL6 knockdown (log2FC >1, *P* value <0.05) (Figure S7b, Supporting Information). Combining the RIP‐seq data and the RNA‐seq data, we found 665 closely RPL6‐associated mRNAs with differential expression in RPL6‐knockdown cells (**Figure** **4**a), Based on previous RNA‐seq data of HCC tissues from orthotopic xenograft mice at four time points, we also revealed that RPL6 was directly involved in several pathways, among which the cholesterol biosynthesis pathway was the most pronounced (Figure 4b). Notably, the mRNA levels of several key enzymes involved in cholesterol synthesis, including HMG‐CoA synthase (HMGCS1), HMG‐CoA reductase (HMGCR), mevalonate kinase (MVK), mevalonate‐5‐pyrophosphate decarboxylase (MVD), farnesyl diphosphate synthase (FDPS), farnesyl‐diphosphate farnesyltransferase 1 (FDFT1), lanosterol synthase (LSS), and lathosterol 5‐desaturase (SC5D), were significantly decreased. Considering that SREBP2, a master transcriptomic factor, is a functional regulator in initializing cholesterol biosynthetic pathways. We then investigated whether RPL6 influences the SREBP2 pathway. Neither SREBP2 expression nor SREBP2 processing (as indicated by the levels of the precursor form [pSREBP2] and the active form [nSREBP2] of SREBP2) was regulated by RPL6 in HCC cells (Figure S7c‐f, Supporting Information), demonstrating that RPL6 did not modulate cholesterol homeostasis via SREBP2. We next screened these eight genes by qRT‐PCR and ultimately focused on HMGCS1, whose mRNA expression dramatically decreased in response to RPL6 knockdown (Figure 4c). Consistently, RPL6 knockdown inhibited the protein expression of HMGCS1 in HCC cells (Figure S7g, Supporting Information). We then performed experiments to elucidate whether the decrease in HMGCS1 mRNA was due to a change in RNA synthesis or RNA decay. The nascent RNA capture assay found that the nascent HMGCS1 mRNA level remained unchanged in RPL6‐knockout cells, and the results of luciferase reporter assay confirmed that RPL6 knockout did not affect the promoter activity of the HMGCS1 gene, while overexpression of RPL6 had no effect on the level of nascent mRNA or the promoter activity of HMGCS1 (Figure S7h,i, Supporting Information). RPL6 knockout significantly reduced the half‐life of HMGCS1 mRNA after actinomycin D treatment, whereas RPL6 overexpression enhanced the stability of HMGCS1 mRNA, as determined by mRNA decay assay (Figure 4d,e). The RNA immunoprecipitation (RIP) assay revealed that HMGCS1 mRNA was significantly enriched in RPL6‐immuneprecipitates compared with the amount of GAPDH mRNA, which served as a negative control (Figure 4f).

We performed depletion mapping analysis and RIP assays to locate the specific fragment of RPL6 that binds to HMGCS1. The results revealed that only the fragments containing the KOW domain (136‐288 aa, T3 and FL) could enrich HMGCS1 mRNA (Figure 4g) and maintain HMGCS1 mRNA stability (Figure 4h), indicating that the KOW domain was responsible for RPL6 mediated HMGCS1 mRNA stability regulation. Moreover, electrophoretic mobility shift assay (EMSA) and RNA pull‐down assay using biotin‐labeled HMGCS1 mRNA showed that RPL6 specifically interacted with the 3′UTR of HMGCS1 but not the 5′UTR or CDS region (Figure 4i; Figure S7j, Supporting Information). We further divided the 3′UTR of HMGCS1 mRNA into six fragments (WT1‐6) and found that the 3301–3720 nt (WT3) fragment located in the HMGCS1‐3′UTR was the main binding site for RPL6 (Figure 4j; Figure S7k, Supporting Information). As consecutive AUUUA motifs serve as the AU‐rich elements (ARE)‐binding sites of RBPs, we focused on the two AUUUA motifs in the HMGCS1‐3′UTR. Intriguingly, mutation of either motif (Mut1 or Mut2) in the HMGCS1‐3′UTR resulted in its inability to bind to RPL6 (Figure 4k; Figure S7l, Supporting Information). Luciferase reporter assays also verified that RPL6 increased HMGCS1 mRNA stability by binding to AUUUA motifs on the HMGCS1‐3′UTR (Figure S7m, Supporting Information). Taken together, these results indicate that RPL6 upregulates HMGCS1 expression by binding HMGCS1 mRNA and increasing HMGCS1 stability.

### 2.5 Upregulated HMGCS1 Enhances HCC Metastasis via Increasing Intracellular Cholesterol Level

**Figure 5:**
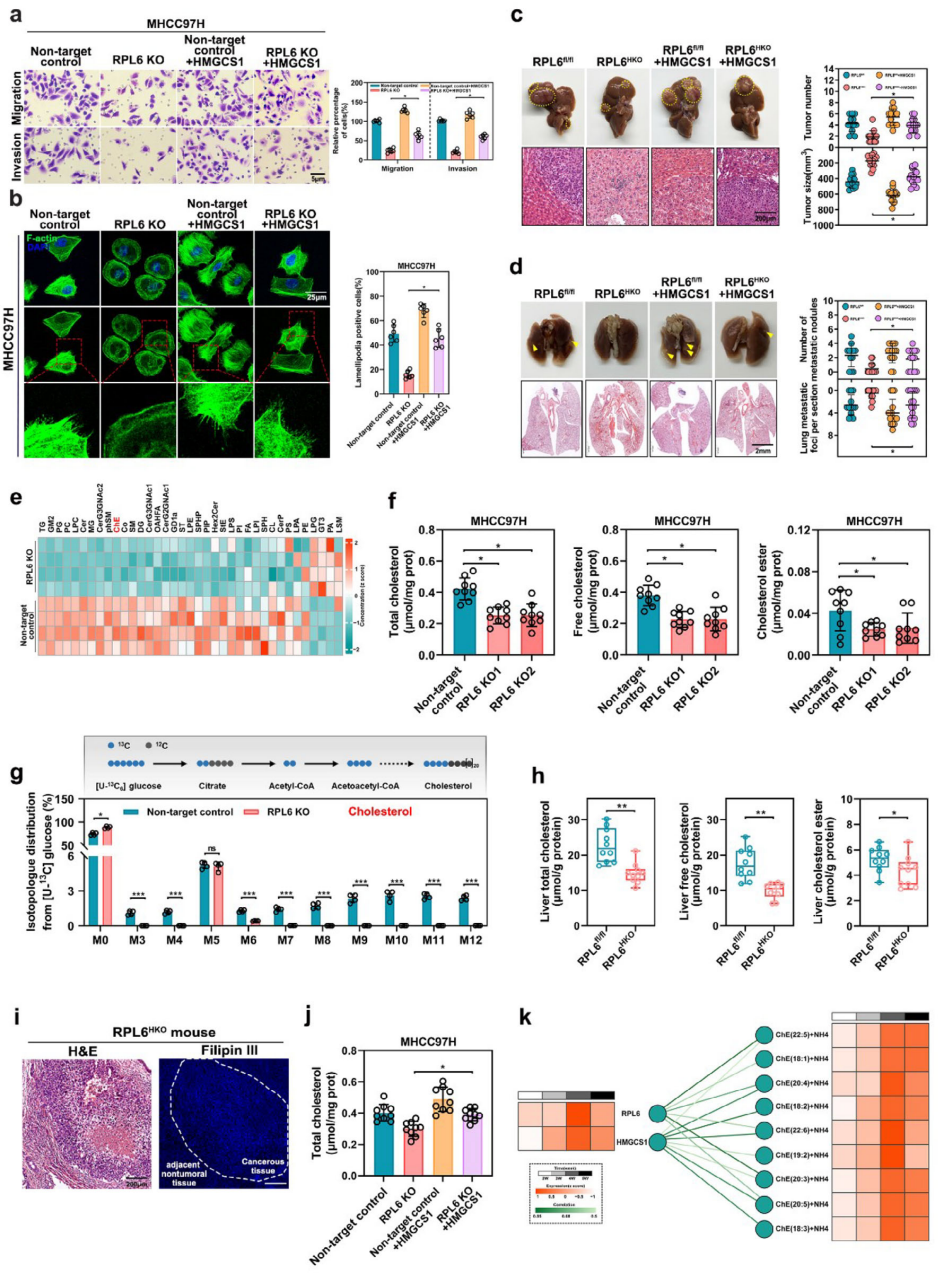
RPL6‐upregulated HMGCS1 expression promotes HCC metastasis by increasing intracellular cholesterol level. a) The effect of HMGCS1 overexpression on the migration and invasion of RPL6 knockout cells was assessed through transwell assays. The cells were counted from 6 images. b) The effect of HMGCS1 overexpression on the lamellipodia formation of RPL6 knockout cells was tested by F‐actin staining assay. Representative images were acquired by confocal, and the percentage of cells with lamellipodia formation were counted from at least six fields. Scar bar, 25 µm. c,d, 10‐month‐old DEN‐treated RPL6^HKO^ or RPL6^fl/fl^ mice were injected via the tail vein with AAV8‐GFP or AAV8‐HMGCS1. The liver and lung were harvested at 8 weeks post‐injection for H&E staining. n = 12‐14 per group. c) Representative macroscopic images and H&E staining of the tumor‐bearing livers in these mice were provided. The tumor number and tumor size were examined. Scar bar, 200 µm. d) Representative macroscopic images and H&E staining of the lungs with tumor metastatic nodules and foci were calculated. Scar bar, 2 mm. e) Heatmap of lipid metabolomics in RPL6 knockout versus non‐target control MHCC97H cells (n = 4). f) Levels of total cholesterol, free cholesterol, and cholesterol ester in RPL6‐knockout MHCC97H cells. g) Isotopologue distribution of cholesterol from ^13^C glucose in RPL6 knockout versus non‐target control MHCC97H cells (n = 4). h) Levels of total, free, and esterified cholesterol in tumor tissues of RPL6^HKO^ and RPL6^fl/fl^ mice (n = 10). i) Liver tumor tissue of RPL6^HKO^ mouse (indicated with dashed lines) and the adjacent normal tissues were stained with HE and Filipin III. Scale bar, 200 µm. j) The total intracellular cholesterol content was quantified in HMGCS1 overexpression of RPL6‐knockout cells. k) Correlation analysis between RPL6 and HMGCS1 mRNA expression levels and the content of cholesterol esters (ChEs) with varying fatty acid chain lengths in the pulmonary metastasis HCC mouse model. Data are shown as mean ± SD of at least three independent experiments (a, b f, h, and j). Statistical significance was determined by two‐tailed unpaired Student's t‐test (e‐h) or one‐way analysis of variance (ANOVA) (a‐d and j). *P < 0.05; **P < 0.01. ChE, cholesteryl ester; H&E, hematoxylin and eosin.

To elucidate the role of HMGCS1 in hepatocarcinogenesis, we conducted several experiments both in vitro and in vivo. Expectedly, HMGCS1 knockdown markedly inhibited HCC cells migration and invasion (Figure S8a–f, Supporting Information). Consistently, HMGCS1 knockdown suppressed tumor formation in the liver and lung metastasis in the orthotopic nude mouse HCC model (Figure S8g–j, Supporting Information). In contrast, HMGCS1 overexpression significantly enhanced the migration and invasion activities of HCC cells (Figure S9a–f, Supporting Information). To further investigate whether HMGCS1 was responsible for RPL6‐mediated HCC metastasis, we overexpressed HMGCS1 in RPL6‐knockout HCC cells. The results showed that overexpressing HMGCS1 markedly restored the migration and invasive capability in RPL6‐knockout HCC cells (**Figure** **5**a,b). Moreover, HMGCS1 overexpression also resulted in increased numbers and sizes of liver tumors, and a higher incidence of lung metastasis in DEN‐treated RPL6^HKO^ mice (Figure 5c,d; Figure S10a, Supporting Information). In contrast, silencing of HMGCS1 markedly diminished the migration and invasive capability in RPL6‐overexpressing HCC cells (Figure S10b,c, Supporting Information). Taken together, these results suggested that HMGCS1 is responsible for RPL6‐mediated HCC metastasis.

Given that HMGCS1 is a key enzyme in cholesterol metabolism, we proposed that RPL6 may upregulate HMGCS1 to increase the intracellular cholesterol level. Lipidomic analysis revealed that the RPL6 knockout led to decreased production of cholesterol ester content (Figure 5e). Indeed, RPL6‐knockout cells exhibited reduced levels of total, free, and esterified cholesterol, with the free cholesterol showing the most significant decrease (Figure 5f), while RPL6 overexpression raised free cholesterol levels (Figure S11a, Supporting Information). To evaluate the impact of RPL6 on cholesterol biosynthesis in HCC cells, we performed tracing experiments using stable isotope‐labelled subtrate, [U^13^C]‐glucose, that feeds into cholesterol synthesis via acetyl‐CoA. The quantification of labeled metabolites using mass spectrometry from HCC cells incubated with [U^13^C]‐glucose indicated that RPL6 knockout cells attenuated cholesterol biosynthesis (Figure 5g). In contrast, overexpression of RPL6 significantly activated cholesterol biosynthesis (Figure S11b, Supporting Information), indicating that RPL6 could activate cholesterol biosynthesis in HCC cells. In parallel, Filipin III staining (a cholesterol probe) showed that RPL6 knockout lowered free cholesterol levels, while RPL6 overexpression raised cholesterol levels (Figure S11c,d, Supporting Information). Meanwhile, the downregulation of cholesterol esters levels was also demonstrated in RPL6 knockout cells via BODIPY staining, and the opposite result was obtained in RPL6‐overexpressing cells (Figure S11e, Supporting Information). Moreover, a reduction of serum cholesterol level, as well as liver tumor cholesterol level were found in RPL6^HKO^ mice (Figure 5h; Figure S11f, Supporting Information). Moreover, the histological analysis confirmed that liver tumors exhibited a substantially higher cholesterol level compared with the adjacent nontumoral tissues (Figure 5i). Importantly, HMGCS1 overexpression markedly restored the cholesterol level of RPL6 knockout cells, while HMGCS1 knockdown notably abolished RPL6‐induced cholesterol biosynthesis (Figure 5j; Figure S11g‐j, Supporting Information). We also examined the correlations between RPL6, HMGCS1, and cholesterol level in the pulmonary metastasis HCC mouse model. By combining transcriptomic data and non‐target lipidomics analysis, correlation analysis revealed a positive correlation between RPL6 and HMGCS1, RPL6 and cholesterol level. In particular, the serum cholesterol level dramatically elevated at the fourth week, which was consistent with the highest expression of RPL6 and HMGCS1 (Figure 5k). Collectively, these data demonstrate that RPL6‐upregulated HMGCS1 enhances HCC metastasis via increasing intracellular cholesterol level.

### 2.6 RPL6‐Mediated Elevation of Cholesterol Facilitates HCC Growth and Metastasis

**Figure 6:**
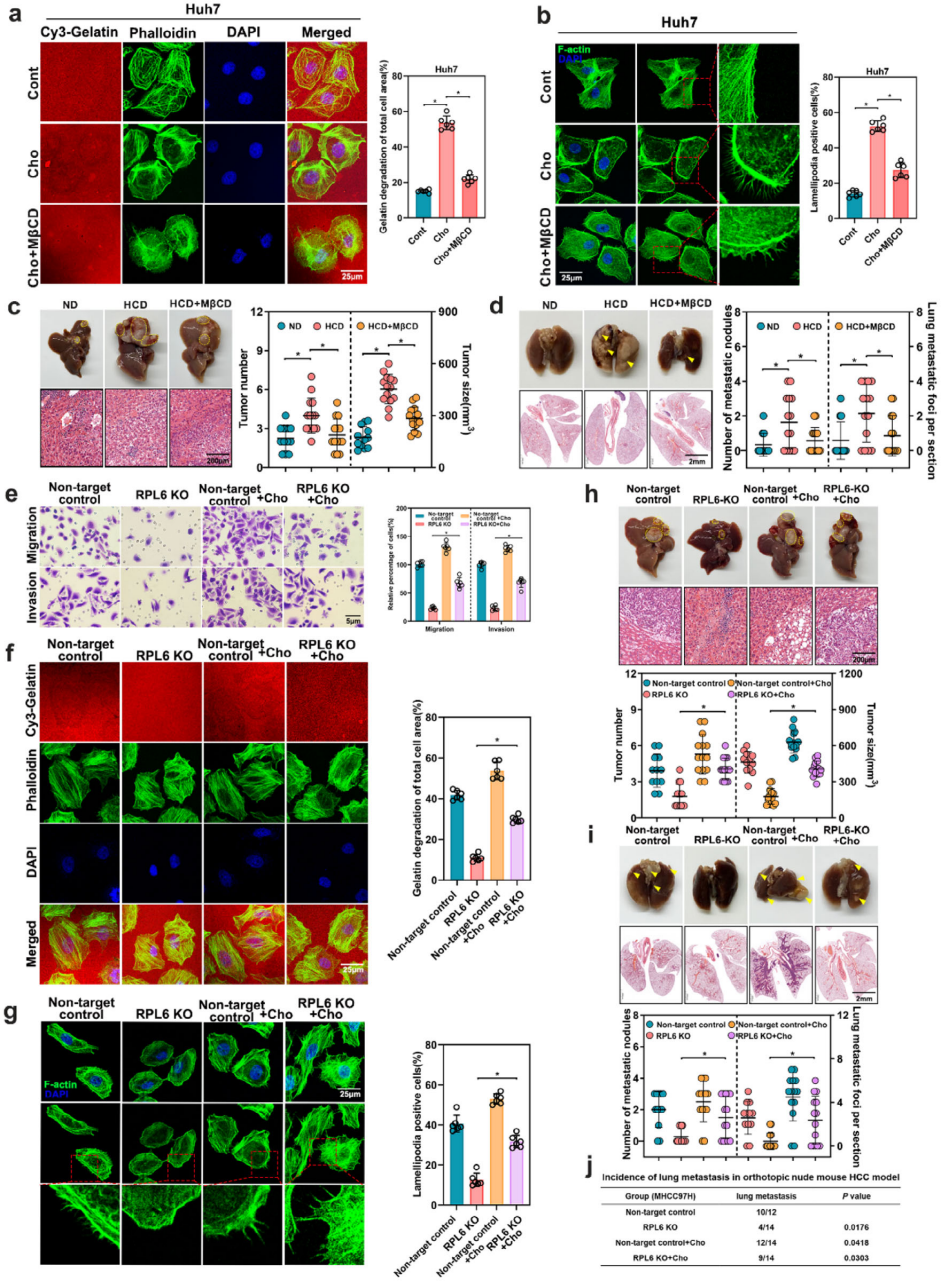
Cholesterol is involved in RPL6‐mediated HCC metastasis. a) The effect of cholesterol (10 µM) and the addition of methyl‐b‐cyclodextrin (MβCD) on invadopodia function in Huh7 cells was tested by gelatin degradation assay. Images were obtained by confocal microscopy and the degraded areas of at least six fields were quantified using image J software. Scale bars, 25 µm. b) The effect of cholesterol (10 µM) and the addition of methyl‐b‐cyclodextrin (MβCD) on lamellipodia formation in Huh7 cells was evaluated through F‐actin staining. Representative images were shown and the percentage of cells with lamellipodia formation was counted from at least six fields. Scale bar, 25 µm. c,d) Huh7 cells were orthotopically injected into the left lobe liver of nude mice, which were then subjected a normal diet (ND), high cholesterol diet (HCD) or HCD+ MβCD (10 mg/kg of mouse, i.p.), to establish a lung metastasis model. Liver and lung tissues collected at 8 weeks post injection were used for detection. n = 12–14 per group. c) Representative macroscopic images and H&E staining of the tumor‐bearing liver from mice on normal diet (ND), high cholesterol diet (HCD) or HCD+ MβCD mice. The tumor number and tumor size were examined. Scar bar, 200 µm. d) Representative macroscopic images and H&E staining of tumor‐metastasis lungs from these mice. The number of metastatic nodules and metastatic foci were examined. Scar bar, 2 mm. e–g) The effect of cholesterol on migration and invasion, invadopodia function, and lamellipodia formation in RPL6‐knockout MHCC97H cells was determined by transwell assays e), gelatin degradation assays f) and F‐actin staining assays g), respectively. h‐j, RPL6‐knockout MHCC97H cells and non‐target control cells were orthotopically injected into the left lobe liver of nude mice, which were then subjected to either an ND or HCD to establish a lung metastasis model. Liver and lung tissues collected at 8 weeks post injection were used for detection. n = 12‐14 per group. h) Representative macroscopic images and H&E staining of tumor‐bearing liver in these mice. The tumor number and tumor size were examined. Scar bar, 200 µm. i) Representative macroscopic images and H&E staining of tumor‐metastasis lungs from these mice. The number of metastatic nodules and metastatic foci were examined. Scar bar, 2 mm. j) Statistical analysis for lung metastasis events of these mice. Data are shown as mean ± SD of at least three independent experiments. Statistical significance was determined by two‐tailed unpaired Student's t‐test (a‐d) or one‐way analysis of variance (ANOVA) (e‐j). *P < 0.05. Cont, control; Cho, cholesterol; MβCD, methyl‐β‐cyclodextrin.

To delineate whether cholesterol promotes the metastasis of HCC, we directly investigated the effect of cholesterol supplementation on HCC metastasis. The cytotoxicity of water‐soluble cholesterol was first determined by using MTT assay in MHCC97H, HLE, Huh7 and PLC/PRF/5 cells, with CC50 > 300 µM in all tested cell lines (FigureS12a, Supporting Information). In particular, cholesterol treatment resulted in the enhancement of migration and invasion, as well as ECM degradation and lamellipodia formation capabilities in Huh7 cells and PLC/PRF/5 cells (**Figure** **6**a,b; Figure S12b–e, Supporting Information). However, cholesterol depletion by methyl‐β‐cyclodextrin (MβCD), an inhibitor that modifies the cholesterol domain on the cellular surface, impaired the effector function of cholesterol on HCC cells (Figure 6a,b; Figure S12b–e, Supporting Information). Meanwhile, we treated RPL6 overexpression HCC cells with simvastatin, which is well‐known inhibitor for cholesterol biosynthesis. Specifically, our findings revealed that simvastatin significantly attenuated the metastatic and invasive abilities in RPL6‐overexpression cells (Figure S12f,g, Supporting Information).

To determine whether RPL6‐mediated elevation of cholesterol facilitates HCC growth and metastasis in vivo, Huh7 cells were transplanted into orthotopic nude mouse HCC model and fed with high cholesterol diet (HCD) or combined with MβCD (Figure S12h, Supporting Information). Before the formal experiment, we first assessed MβCD toxicity in nude mice. As shown in Supporting Table S2, no significant differences in toxicity were found upon treatment with MβCD at either concentration (TableS2, Supporting Information). Then, 10 mg/kg was selected as the dose of MβCD used for further study. Compared to control mice, HCD‐fed mice showed significantly increased body weight, fasting glucose, and impaired glucose tolerance (Figure S12i‐k, Supporting Information). However, MβCD treatment attenuated these effects. Moreover, increased liver weight, liver/body weight ratio, hepatic triglyceride and cholesterol ester, as well as serum cholesterol level were observed in HCD‐fed mice, which could be diminished by MβCD treatment (Figure S12l‐p, Supporting Information). then, the effects on HCC growth and metastasis were examined. HCD‐fed mice exhibited increased tumor numbers and tumor sizes, and a higher incidence of lung metastasis, while MβCD treatment limited tumor growth and lung metastasis induced by cholesterol (Figure 6c,d; Figure S12q, Supporting Information). These findings demonstrate that dietary cholesterol augments HCC growth and metastasis.

We next examined whether cholesterol mediated the effect of the RPL6‐ HMGCS1 axis on HCC metastasis. We treated RPL6‐knockout cells or HMGCS1‐knockdown cells with cholesterol and found that the addition of cholesterol significantly restored the migration and invasion ability of these cells (Figure 6e‐g; Figure S13a‐c, Supporting Information). RPL6 knockout or HMGCS1 knockdown markedly reduced the tumor growth and invasive and metastatic potential of HCC cells in the liver orthotopic nude mouse HCC model, and these effects were markedly reversed by cholesterol supplementation (Figure 6h‐j; Figure S13d‐f, Supporting Information). Taken together, these results indicated that RPL6 promotes HCC growth and metastasis in a cholesterol‐dependent manner.

### 2.7 Cholesterol Activates the HIF‐1 Signaling Pathway Through Inhibiting the Proteasome‐Mediated Degradation of HIF‐1α

**Figure 7:**
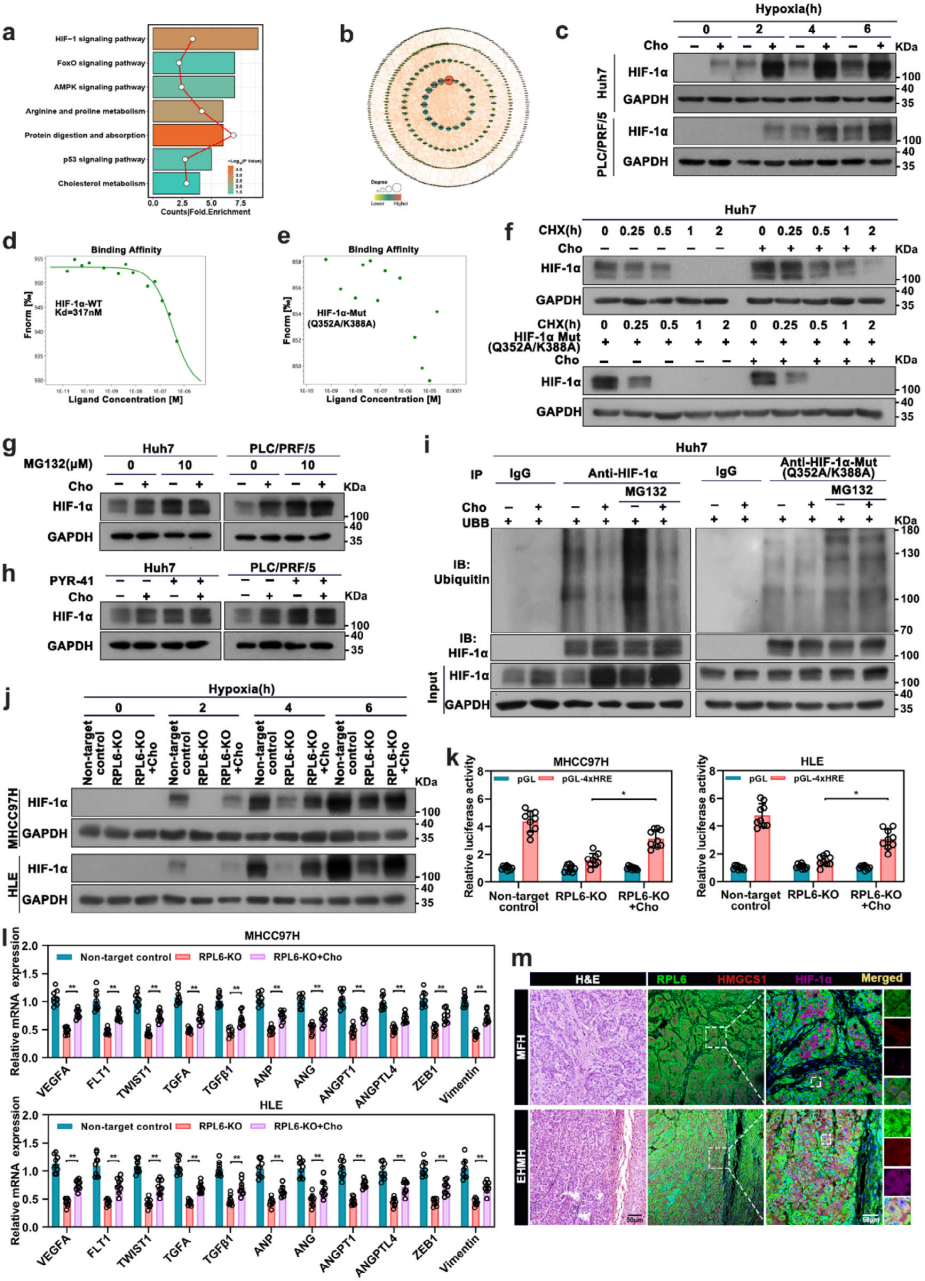
Cholesterol stabilizes HIF‐1α through inhibiting proteasome‐mediated degradation in HCC. a) KEGG analysis of differentially expressed proteins in control and cholesterol‐treated (10 µM) Huh7 cells was performed by proteomic data. b) The network of differentially expressed proteins was visualized using STRING database. The size of circles presented the degree of linkage between proteins. c) Huh7 and PLC/RPF/5 cells treated with cholesterol (10 µM) were exposed to hypoxia for 6 h and harvested at the indicated times. The impact of cholesterol treatment on HIF‐1α protein levels was determined by western blot. d) Microscale thermophoresis (MST) was used to investigate the binding of cholesterol with HIF‐1α. His‐tagged HIF‐1α (20 nM) was mixed with 10 µL of cholesterol (10 µM). Different concentrations of cholesterol were mixed with His‐tagged HIF‐1α to analyze Binding Affinity analysis, and the dissociation constant (Kd) was calculated. e) Microscale thermophoresis (MST) was used to investigate the binding of cholesterol with HIF‐1α‐MUT (Q352A/K388A). f) Cells treated with cholesterol (10 µM) were exposed to hypoxia for 6 h (upper), or cells transfected with HIF‐1α‐MUT plasmids for 24 h (lower) then incubated with 10 µg/mL cycloheximide at the indicated times. g) Cells treated with cholesterol (10 µM) and subjected to hypoxia for 6 h, were exposed to the proteasome inhibitor MG132 (10 µM) for 6 h. Subsequently, HIF‐1α protein levels were analyzed by western blotting. h) Following 6‐hour hypoxia treatment with 10 µM cholesterol, cells were treated with PYR‐41 (50 µM) for another 6 hours. HIF‐1α protein levels were analyzed by western blotting. i) Huh7 cells transfected with HA‐Ub plasmid for 24 h and treated with cholesterol (10 µM) under hypoxia for 6 h (left) or transfected with HIF‐1α‐MUT plasmid for 24 h (right), then exposed to MG132 (10 µM) for 6 h. Whole‐cell extracts were immunoprecipitated with anti‐HIF‐1α antibody, and ubiquitinated‐HIF‐1α was detected using an anti‐ubiquitin antibody. j) The impact of cholesterol addition on HIF‐1α protein levels in RPL6‐knockout cells was determined by western blot. RPL6‐knockout cells treated with cholesterol (10 µM) were exposed to hypoxia for 6 h and harvested at the indicated times. k) The transcriptional activity of HIF‐1α in RPL6‐knockout cells with or without cholesterol was assessed using dual‐luciferase reporter assays. Cells were co‐transfected with 4 × HRE‐luc and Renilla plasmids, incubated with cholesterol for 24 h, and then exposed to hypoxia for 6 h. Luciferase activity was detected and normalized to Renilla activity. l) The mRNA levels of HIF‐1α targeted genes in RPL6‐knockout cells with or without cholesterol treatment were evaluated. HCC cells were exposed to hypoxia for 6 h. m H&E staining and representative images of RPL6 (green), HMGCS1 (red), and HIF‐1α (purple) protein expression in MFH and EHMH tissues by multiple immunofluorescence (mIFC) staining. Scale bar, 50 µm. Data are shown as mean ± SD of at least three independent experiments (c‐l). Statistical significance was determined by two‐tailed unpaired Student's t‐test (k,l). *P <0.05; **P <0.01. CHX, cycloheximide.

To elucidate the intricate molecular mechanism underlying the oncogenic role of cholesterol in HCC metastasis, we performed proteomic analysis to discern downstream targets in cholesterol‐treated cells. A total of 505 proteins were found to be differentially expressed, including 296 upregulated proteins and 209 downregulated proteins. We then performed Kyoto Encyclopedia of Genes and Genomes (KEGG) pathway analysis and found that these differently expressed genes effectively participated in many cancer‐related pathways, among which the top‐ranked pathways were the HIF‐1 signaling pathway, FOXO signaling pathway, and AMPK signaling pathway (**Figure** **7**a). PPI network analysis further revealed that HIF‐1α interacted with the most differentially expressed proteins and served as the central regulatory node in the cholesterol perturbation network (Figure 7b). Consistently, we employed proteomics assays on cells with RPL6 knockout and cholesterol replenishment. A total of 1500 proteins were identified, which exhibited four distinct non‐linear expression patterns (Figure S14a, Supporting Information). We focused on the proteins enriched in Cluster 1, which downregulated by RPL6 depletion, while rescued by cholesterol replenishment. Then, KEGG enrichment analysis found that the HIF‐1 pathway was also significantly enriched in Cluster 1 (Figure S14b, Supporting Information). More importantly, HIF‐1 signaling pathway is one of the metastasis initiation associated pathways which directly related to RPL6 (Figure 1f), suggesting the potential role of HIF‐1α in cholesterol‐mediated HCC metastasis.

We next investigated the mechanism underlying how cholesterol regulates the HIF‐1 signaling pathway in HCC cells. The addition of exogenous cholesterol induced elevated HIF‐1α protein levels in HCC cells under hypoxic conditions (Figure 7c). To investigate the relationship between cholesterol and HIF‐1α, we first used microscale thermophoresis (MST) to investigate whether there is a direct binding between cholesterol with HIF‐1α. The data revealed that cholesterol can directly interact with HIF‐1α (Figure 7d). Subsequently, molecular docking simulations suggested that the 352nd glutamine and 388th lysine residues in the ODD domain of HIF‐1α are the binding sites for cholesterol (Figure S14c, Supporting Information). To validate the above results, we introduced double mutant of HIF‐1α by replacing the glutamine and lysine residues with the alanine residue (HIF‐1α Q352A/K388A). The Q352A/K388A mutation almost completely abolished the binding between HIF‐1α and cholesterol (Figure 7e), suggesting that Gln352/Lys388 might play an essential role in the binding of cholesterol to HIF‐1α. We then sought to determine whether cholesterol affects HIF‐1α protein stability in HCC cells treated with cycloheximide to block *de novo* protein synthesis. Double mutant of HIF‐1α (HIF‐1α Q352A/K388A) was constructed and the effect of cholesterol on its stability was evaluated. Expectedly, cholesterol significantly enhanced the stability of HIF‐1α, but had no significant impact on the stability of the mutant HIF‐1α (HIF‐1α Q352A/K388A) (Figure 7f; Figure S14d‐f, Supporting Information), indicating that the regulatory effect of cholesterol on HIF‐1α stability relies on their direct interaction. The enhancement of cholesterol on HIF‐1α protein expression was blocked in the presence of proteasome inhibitor MG132 (Figure 7g), suggesting that cholesterol increases HIF‐1α protein stability via inhibiting proteasome‐mediated degradation. Moreover, cholesterol‐treated cells were treated with PYR‐41 (50 µM), a specific inhibitor of the ubiquitin‐activating enzyme E1, thereby inhibiting whole‐cell ubiquitination. PYR‐41 treatment blocked the enhancement of HIF‐1α protein expression induced by cholesterol (Figure 7h), suggesting that the ubiquitination machinery is involved in cholesterol‐mediated stabilization of HIF‐1α. Previous data showed that E3 ubiquitin ligases such as VHL, MDM2, FBXW7, and RACK1 are involved in the regulation of HIF‐1α stability.^[^ ^28^, ^29^ ^]^ We also screened the HIF‐1α‐binding E3 ubiquitin ligases by using co‐immunoprecipitation in HCC cells. Our data revealed that VHL and MDM2 could bind to HIF‐1α, indicating that those two E3 ubiquitin ligases were responsible for HIF‐1α protein stabilization in HCC (Figure S14g, Supporting Information). Expectedly, the binding of VHL and MDM2 to the HIF‐1α was incrementally decreased in the presence of cholesterol, while the binding of VHL and MDM2 to the mutant HIF‐1α remained unchanged (Figure S14h, Supporting Information). Consistently, the level of ubiquitinated HIF‐1α decreased significantly in response to cholesterol under hypoxia, and that was largely unchanged in mutant HIF‐1α (Figure 7i; Figure S14i, Supporting Information). These results suggested that cholesterol could stabilize the HIF‐1α protein by inhibiting its ubiquitin‐dependent degradation.

Importantly, the expression of HIF‐1α was significantly decreased in RPL6‐knockout cells, whereas it was restored by cholesterol treatment (Figure 7j). Given that HIF‐1α activates the expression of downstream target genes by binding to the highly conserved hypoxia‐responsive element (HRE) in target genes. Therefore, we first performed the HRE‐luciferase reporter assay to examine the whole transcriptional activity of downstream genes in RPL6‐knockout cells with or without cholesterol. As expected, RPL6 knockout led to obvious downregulated of luciferase activity, which could be rescued by cholesterol administration (Figure 7k). Furthermore, the expression of downstream tumor metastasis‐related target genes of HIF‐1α was detected. Consistently, the expression of these downstream target genes was inhibited in RPL6‐knockout cells, while it was restored by cholesterol treatment (Figure 7l). Importantly, the co‐expression of RPL6, HMGCS1, and HIF‐1α was examined by multicolor immunofluorescence staining of primary HCC tissues with extrahepatic metastasis and metastasis‐free HCC tissues. The EHMH group had higher protein expression of RPL6, accompanied with high HMGCS1 and HIF‐1α expression (Figure 7m; Figure S14j, Supporting Information). Furthermore, correlation analysis in pulmonary metastasis HCC mouse model demonstrated that the protein expression levels of RPL6 were positively correlated with those of HMGCS1 and HIF‐1α (R = 0.91 and 0.87, respectively), as well as HMGCS1 and HIF‐1α (R = 0.93) (Figure S14k,l, Supporting Information). Collectively, we found that RPL6/cholesterol axis promotes HCC metastasis by activating the HIF‐1 signaling pathway.

### 2.8 Antitumor Effects and Targeting Potential of PLGA‐siRPL6/NPs in HCC Models

**Figure 8:**
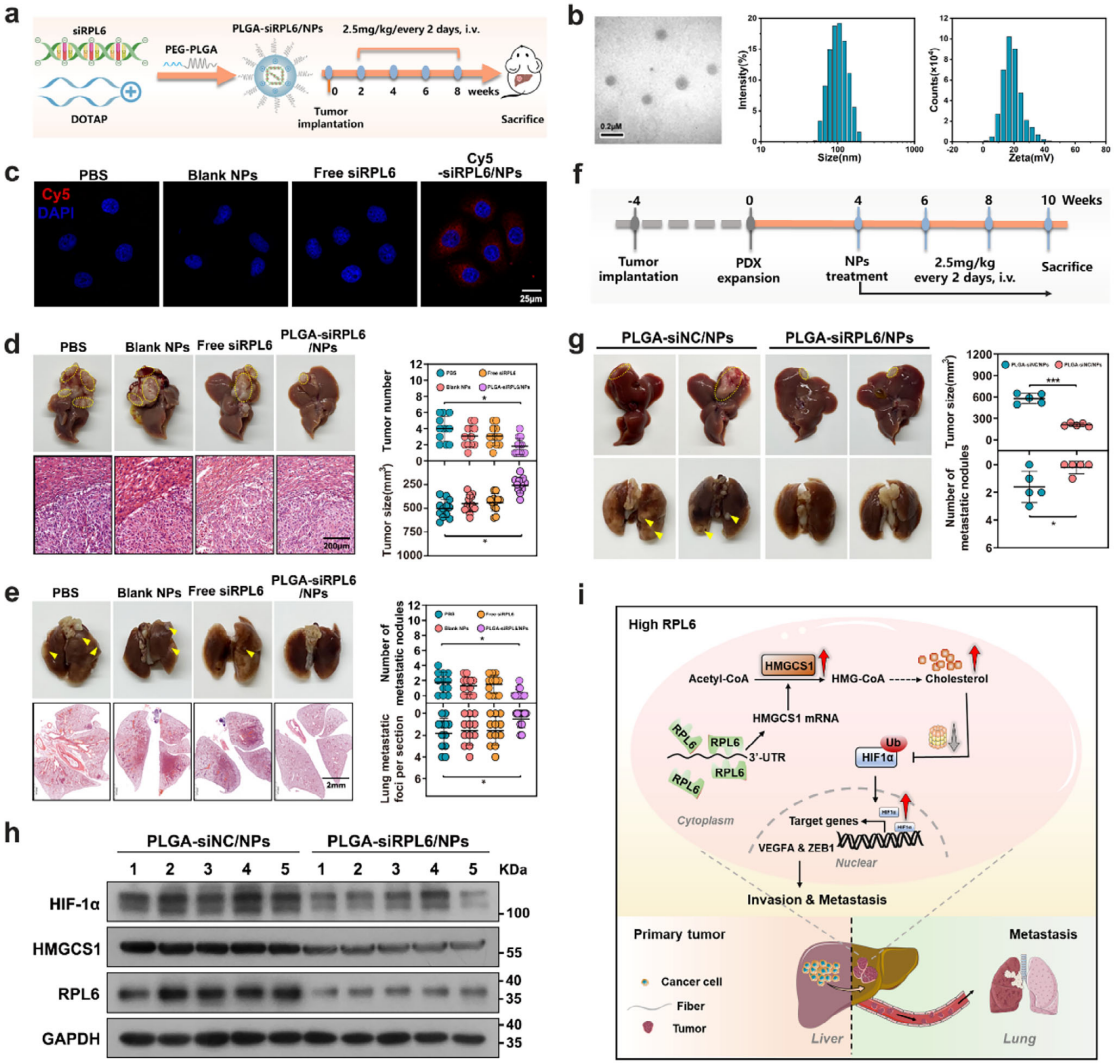
The anti‐tumor effect of PLGA‐siRPL6/NPs in HCC. a) Scheme indicating the step‐by‐step synthesis of PLGA‐siRPL6/NPs and the subsequent experiment design for treatments in vivo. b) Representative TEM images of PLGA‐siRPL6/NPs. Scale bar, 0.2 µm (left). Particle size distribution (middle) and Zeta potential values (right) of PLGA‐siRPL6/NPs were determined by Dynamic Light Scattering. c) The cellular uptake of HCC cells incubated with Cy5‐siRNA/NPs, blank NPs, free siRNA, or PBS control at 37 °C for 2 h was evaluated by fluorescence assay. The siRNA was labeled with Cy5 (red), and cell nuclei were stained with DAPI (blue). Scar bars, 25 µm. d,e, MHCC97H cells were orthotopically injected into the left lobe liver of nude mice to establish a lung metastasis model, followed by the indicated treatments. Liver and lung tissues collected at 8 weeks post‐injection were used for detection. n = 12 per group. d) Representative macroscopic pictures and H&E staining of tumor‐bearing liver in these mice are shown. The tumor number and tumor size were examined. Scar bar, 200 µm. e) Representative macroscopic images and H&E staining of the lungs with tumor metastasis in these mice are shown. The number of metastatic nodules and metastatic foci was examined. Scar bar, 2 mm. f) Illustration of the therapeutic study based on the HCC patient‐derived xenograft (PDX) model. Tumor tissues from patients were implanted into the liver of mice, which then injected with PLGA‐siNC/NPs or PLGA‐siRPL6/NPs every 2 days for 6 weeks. n = 5 per group. g) The effect of siRPL6 on the PDX model (left), and the tumor size and metastatic nodules were examined (right). h) Western blot analysis of HIF‐1α, HMGCS1 and RPL6 expression in PDX tumors. i) Mechanistic model of RPL6‐mediated HMGCS1 mRNA stability to promote HCC migration. In HCC cells, RPL6 expression level was significantly upregulated, and RPL6 enhanced HMGCS1 mRNA stability by binding to HMGCS1 3′UTR that increases cholesterol level. Elevated cholesterol protects HIF‐1α from degradation by ubiquitin proteasome, then stable HIF‐1α further enhances the expression of downstream metastasis‐associated genes, ultimately promoting HCC metastasis. Data are shown as mean ± SD (d,e,g). Statistical significance was determined by two‐tailed unpaired Student's t‐test (g) or one‐way analysis of variance (ANOVA) (d,e). *P < 0.05; ***P < 0.001. PDX, patient‐derived xenograft; NPs, nanoparticles.

To investigate the potential utility of RPL6 as a therapeutic target for HCC, we established a cationic liposome‐modified PLGA‐PEG nanoparticle system based on encapsulated siRNA targeting RPL6 (PLGA‐siRPL6/NPs) (**Figure** **8**a). Transmission electron microscope (TEM) displayed a consistent spherical morphology with a particle size of approximately 100 nm, and dynamic light scattering (DLS) analysis further confirmed a hydrodynamic size of 105.51±1.12 nm for these nanoparticles (Figure 8b). Moreover, surface zeta potential analysis exhibited a zeta potential of 10.2±2.31 mV for the PLGA‐siRPL6/NPs (Figure 8b). The assessment of cellular uptake of NPs in MHCC97H cells using Cy5‐siRNA as an indicator revealed that the PLGA‐siRPL6 complex promoted the cellular uptake and internalization of siRNAs better than did free siRNA (Figure 8c). PLGA‐siRPL6/NPs could effectively reduce the expression of RPL6 and subsequently inhibit proliferative capacity of HCC cells (Figure S15a‐c, Supporting Information). In vivo fluorescence imaging data showed that the PLGA‐Cy5‐siRPL6/NPs complex accumulated predominantly in the liver compared with control group (Figure S15d, Supporting Information), suggesting that PLGA‐Cy5‐siRPL6/NPs can specifically target liver tumors in vivo. To evaluate the antitumor effects of PLGA‐siRPL6/NPs in vivo, the human HCC orthotopic nude mouse model established using MHCC97H cells was treated via the tail vein injection of PBS, free siRPL6, blank NPs or PLGA‐siRPL6/NPs. We first confirmed the absence of significant hepatotoxicity and nephrotoxicity of PLGA‐siRPL6/NPs by assessing blood indices such as aspartate transaminase (AST), alanine transaminase (ALT), creatinine (Cr), and blood urea nitrogen (BUN) levels (Figure S15e,f, Supporting Information). Compared with control mice, mice receiving the PLGA‐siRPL6/NPs treatment exhibited a pronounced reduction in liver tumor size and volume (Figure 8d). The number of lung metastases in the PLGA‐siRPL6/NPs‐treated mice was significantly lower than that of the corresponding control mice (Figure 8e; Figure S15g, Supporting Information). In addition, the PLGA‐siRPL6/NPs complex significantly inhibited the expression of RPL6, HMGCS1, and HIF‐1α in liver tumor tissues (Figure S15h, Supporting Information).

In addition, we determined the therapeutic effect of targeting RPL6 in patient‐derived xenograft (PDX) model with RPL6 upregulation (Figure 8f). Four weeks after implantation, the NOD/SCID IL2rg^‐/‐^ (NSG) mice were injected with PLGA‐siRPL6/NPs or PLGA‐siNC/NPs via the tail vein. No significant differences in toxicity were found upon treatment with PLGA‐siRPL6/NPs at either concentration (Table S3, Supporting Information). The results showed that treatment with siRPL6 nanoparticles markedly reduced liver tumor volume and lung metastasis compared with those in the control group (Figure 8g). Moreover, PLGA‐siRPL6/NPs treatment decreased the protein levels of RPL6, HMGCS1, and HIF‐1α in the liver tumors of the PDX model mice (Figure 8h). Taken together, these results suggest that targeting RPL6 could offer an effective and potential therapeutic for HCC.

## 3 Discussion

The high propensity for metastasis in HCC significantly contributes to its high mortality rate and poor prognosis.^[^ ^30^ ^]^ Therefore, the identification of predictive biomarkers for the premetastatic phase, along with a comprehensive understanding of the molecular mechanisms initiating metastasis, is crucial for developing effective strategies for early detection and prevention. To this end, we developed a pulmonary metastasis HCC mouse model and employed the DNB approach to investigate the dynamic transcriptomic profiles of tumor samples and identify the tipping point in the HCC metastasis process. In contrast to traditional molecular biomarkers, which detect disease transition states through the differential expression of molecules, the DNB methodology excels in detecting early‐warning signals of phase shifts through the dynamic analysis of robust collective fluctuations within gene networks.^[^ ^31^, ^32^ ^]^ In this study, we analyzed time‐series transcriptomic data by using the DNB method. Accordingly, we identified 142 DNB members and corresponding DNB‐neighboring genes involved in metastasis initiation, which were associated with ECM‐receptor interaction, focal adhesion, regulation of actin cytoskeleton, and tight junction. Consistent with these previous studies,^[^ ^7^, ^31^ ^]^ the expression level of DNB‐neighboring genes reversed before and after the fourth week during metastasis. Notably, RPL6, a component of the large ribosomal subunit (RPLs), was identified to play a key role in metastasis initiation based on the DNB ranking and network rewiring.

RPL6, which is traditionally recognized as a ribosomal protein, plays a crucial role in protein synthesis by participating in the assembly and functional dynamics of ribosomes.^[^ ^33^ ^]^ Recent studies have revealed the dysregulated expression and significant roles of RPL6 in various diseases, including cancer, Parkinson's disease, and Plasmodium infection.^[^ ^22^, ^34^, ^35^ ^]^ These studies have also shed light on the extraribosomal functions of RPL6, delineating its participation in disease regulation independent of its conventional role in protein translation. It has been reported that RPL6 could bind to and inhibit the E3 ubiquitin ligase activity of HDM2, which in turn stabilizes and activates p53 to decelerate cell cycle progression.^[^ ^23^ ^]^ In gastric cancer cells, RPL6 promotes cell growth and cell cycle progression via the upregulation of cyclin E.^[^ ^25^ ^]^ Interestingly, RPL6 can be recruited to DNA damage sites in a poly (ADP‐ribose) polymerase (PARP)‐dependent manner and directly interacts with histone H2A, underscoring its critical involvement in the DNA damage response (DDR).^[^ ^21^ ^]^ In this study, RPL6 expression was significantly upregulated in HCC tissues with extrahepatic metastasis and strongly correlated with poorer prognosis in HCC patients. RPL6 knockout diminished the invasive ability of HCC cells in vitro and in vivo, confirming that RPL6 is a key driver of HCC metastasis. Given the recent classification of RPL6 as an RBP that orchestrates cellular functions through interactions with target molecules, we combined RNA‐seq and RIP‐seq data to examine the direct targets of RPL6 in HCC cells and ultimately focused on HMGCS1, a key enzyme in the mevalonate pathway of cholesterol synthesis.^[^ ^36^, ^37^ ^]^ We further revealed that RPL6 directly bound to HMGCS1 mRNA and increased its stability, revealing a novel extraribosomal function of RPL6 in the regulation of HCC metastasis.

The aberrant activation of cholesterol biosynthesis constitutes a distinct hallmark of tumors and plays a pivotal role in tumor development.^[^ ^38^ ^]^ It facilitates the synthesis of lipids necessary for constructing cell membranes in rapidly proliferating tumor cells and conducting essential signaling cascades.^[^ ^39^ ^]^ Several enzymes involved in the biosynthesis of cholesterol have been shown to be dysregulated in various cancers. HMGCR and SQLE, two rate‐limiting enzymes in the pathway, are significantly upregulated in various types of tumors and act as promoters of tumor proliferation and metastasis.^[^ ^40^, ^41^ ^]^ Moreover, the aberrant expression of several enzymes, such as HMGCS1, FDPS, ACAT2, and FDFT1, is significantly correlated with aggressive tumor phenotypes and poor clinical outcomes.^[^ ^42^, ^43^ ^]^ Cholesterol and its metabolic byproducts, including cholesterol esters and oxysterols, have been recognized as crucial metabolites that contribute to tumor cell proliferation and metastasis.^[^ ^44^ ^]^ However, the role of cholesterol in HCC progression remains inconsistent. Numerous studies have revealed that HCC cells typically upregulate cholesterol biosynthesis and uptake, and reduce cholesterol export and excretion, to acquire enough building blocks for proliferation, metastasis, and drug resistance.^[^ ^39^ ^]^ However, another study revealed that a cholesterol‐rich diet increased the relocation of CD44 in membrane lipid rafts, thereby limiting HCC metastasis.^[^ ^45^ ^]^ Our study here unveils a previously unappreciated upregulation of cholesterol biosynthetic pathway driven by high RPL6 expression, augmenting HMGCS1 in HCC cells, leading to increase intracellular cholesterol levels. Indeed, Cholesterol treatment markedly enhanced the tumor growth and invasive ability of HCC cells with low‐metastatic potential both in vitro and in vivo, while either cholesterol depletion by MβCD or de novo cholesterol synthesis blockade by simvastatin impaired the effect of cholesterol on HCC cells. These findings revealed that cholesterol acts as an important oncometabolite in HCC tumorigenesis and metastasis. Moreover, the addition of cholesterol significantly restored the migration and invasion ability of RPL6‐knockout or HMGCS1‐knockdown cells. These results demonstrated that intracellular cholesterol level mediated by HMGCS1, at least in part, is responsible for the prometastatic function of RPL6. We could not rule out the possibility that other metabolic pathways or metabolites may participate in the protumorigenic function of RPL6, warranting further investigations.

Cholesterol is an essential biomolecule involved not only in the maintenance of membrane permeability and fluidity, but also acts as a signaling molecule in the modulation of many signaling pathways.^[^ ^46^ ^]^ Cholesterol can bind to and induce a conformational alteration in FAF2, promoting the assembly of the SNHG6–FAF2–mTOR complex and thereby activating the mTORC1 kinase cascade to accelerate HCC proliferation.^[^ ^47^ ^]^ Additionally, cholesterol directly interacts with Smoothened (SMO), facilitating SMO cholesterylation, which in turn triggers the allosteric activation of SMO and the activation of the Hedgehog signaling pathway.^[^ ^48^, ^49^ ^]^ Furthermore, cholesterol directly binds to the transmembrane domain of PD‐L1 and stabilizes the PD‐L1 protein, which aids in immune evasion in cancer cells.^[^ ^50^ ^]^ Moreover, the interaction of cholesterol with Frizzled5 (Fzd5), which leads to Fzd5 palmitoylation and plasma membrane localization, induced the activation of the Wnt/β‐catenin signaling pathway to promote tumor growth.^[^ ^51^ ^]^ These findings indicate that cholesterol serves as a signaling molecule, binding to proteins and modulating their conformation, stability, enzymatic activity, and posttranslational modifications. In this study, a direct interaction between cholesterol and HIF‐1α was evidenced by MST and molecular docking analyses. And our study has revealed that cholesterol plays a pivotal role in the modulation of multiple critical pathways, especially in regulating the stability of HIF‐1α protein, which subsequently influences multiple steps within the metastatic cascade of HCC cells, including affecting cytoskeleton remodeling, epithelial‐mesenchymal transition (EMT), promoting tumor angiogenesis, and facilitating tumor immune evasion.^[^ ^52^, ^53^ ^]^ This enhancement is positively correlated with the metastatic potential of HCC. Overall, our data linked cholesterol metabolism, intratumor hypoxia, and HIF‐1α expression, which could endow HCC cells with increased metastatic ability.

In summary, we used the DNB method based on a pulmonary metastasis HCC mouse model and identified RPL6 as a core DNB member for metastasis initiation. Elevated RPL6 expression was found in HCC tissues with extrahepatic metastasis, and RPL6 overexpression promoted HCC growth and metastasis. Mechanistically, RPL6 interacts with the HMGCS1 mRNA 3′UTR to increase its mRNA stability, leading to increased cholesterol levels. Increased cholesterol promotes cancer metastasis in HCC by stabilizing HIF‐1α protein (Figure 8i). We propose that RPL6 represents an important biomarker of the initiation of metastasis and a potential therapeutic target for the treatment of HCC.

## 4. Experimental Section

### Cell Culture

The Huh7 and HLE human HCC cell lines were purchased from the Health Science Research Resource Bank. MHCC97H cells were obtained from the Liver Cancer Institute of Zhongshan Hospital of Fudan University (Shanghai, China). The PLC/PRF/5 and HEK293 FT cell lines were purchased from American Type Culture Collection (ATCC). All cell lines were cultured in Dulbecco's modified Eagle's medium (DMEM) at 37 °C in a 5% CO_2_ incubator. The medium was supplemented with 10% FBS and 1% penicillin/streptomycin. These cell lines were routinely authenticated and were free of mycoplasma contamination. For hypoxia conditions, cells were cultured in a low oxygen incubator equilibrated with certified gas containing 1% O_2_, 5% CO_2_, and 94% N_2_.

### Antibodies, Plasmids, and Other Reagents

The following antibodies were used in this study: anti‐RPL6 (NBP2‐20216; Novus Biologicals; 1:300), anti‐FLAG‐tag (F1804; Sigma; 1:5000), anti‐HMGCS1 (17643‐1‐AP; Proteintech; 1:3000, sc‐373681; Santa Cruz; 1:400), anti‐SREBP2 (28212‐1‐AP; Proteintech; 1:2000), anti‐VHL (24756‐1‐AP; Proteintech; 1:2000), anti‐MDM2 (27883‐1‐AP; Proteintech; 1:3000), anti‐FBWX7 (28424‐1‐AP; Proteintech; 1:2000), anti‐RACK1 (27592‐1‐AP; Proteintech; 1:3000), anti‐HIF‐1α (36169S; CST; 1:1000), anti‐ubiquitin (3936S; CST; 1:1000), anti‐IgG (3900S; CST; 1:1000), goat anti‐rabbit IgG‐HRP (NA934v; GE Healthcare; 1:3000), goat anti‐mouse IgG‐HRP (NA931v; GE Healthcare; 1:5000), and anti‐GAPDH (51332S; CST; 1:10000).

All lentivirus‐driven short hairpin RNAs (shRNAs) against target genes were purchased from GeneChem Co., Ltd. (Shanghai, China). The coding sequence (CDS) of RPL6 was acquired from Youbao Biotechnology (China) and cloned and inserted into the p3 × FLAG‐CMV‐7.1 vector. Lentiviruses overexpressing RPL6 and HMGCS1 were purchased from GeneChem Co., Ltd., and subcloned and inserted into the pCDH‐EF1‐copGFP‐T2A‐Puro vector (72263, Addgene). The RPL6, HMGCS1, and HIF‐1α truncation plasmids and the pGL3‐derived reporter vector were obtained from Youbao Biotechnology (China). The ODD‐luciferase reporter plasmid was purchased from Addgene (18965). All sequences are listed in Table S4 (Supporting Information).

TRNzol (DP405, TIANGEN), MG‐132 (HY‐13259, MCE), T4 DNA ligase (M0202, BioLabs), DyLight 488 phalloidin (12935S, CST), DAPI (D1306, Thermo), puromycin (S7417, Selleck), PYR41 (S7192, Selleck), mitomycin C (S8146, Selleck), actinomycin D (S8964, Selleck), cycloheximide (97064‐722, Amresco), filipin III (HY‐N6718, MCE), cholesterol (C4951, Sigma), MβCD (C4555, Sigma), Simvastatin (HY‐17502, MCE), SYTOX Deep Red (P36990, Invitrogen), SYBR Green (Bio‐Rad), BeyoECL Plus (P0018S, Beyotime), and Lipofectamine 3000 (L3000001, Invitrogen) were obtained from the indicated vendors.

### Orthotopic Liver Xenograft Tumors

To construct an in vivo orthotopic mouse model of HCC, BALB/c nude mice (male, 6–8 weeks of age) were purchased from Cavens Biogle (Suzhou, China). To establish orthotopic transplantation HCC models, 1 × 10^7^ HCC cells with stable expression of the indicated genes in 50% Matrigel (356234, Corning) were orthotopically injected into the left lateral lobe of the liver. Mice were sacrificed at 8 weeks after implantation, and tumor volumes were determined by external measurements and calculated according to the following equation: V = [L × W2] × 0.5 (V = volume, L = length, and W = width). The metastatic lung tumor nodules and foci were counted using H&E staining. The animal studies were performed in strict accordance with the Animal Research: Reporting of In Vivo Experiments (ARRIVE) guidelines, and all the experimental procedures were approved by the Experimental Ethics Committee of Chongqing Medical University (IACUC‐CQMU‐2023‐0382).

For the orthotopic mouse model of MHCC97H‐GFP for lung metastasis of HCC, after orthotopic tumor implantation with GFP‐tagged MHCC97H (MHCC97H‐GFP) cells, the mice were euthanized and dissected at the second, third, fourth and fifth weeks, and liver tumors and lung tissues were analyzed by fluorescence imaging (IVIS Spectrum; PerkinElmer, USA). The liver tumors collected at the indicated time points were immediately frozen in liquid nitrogen for the assessment of genome‐wide expression; mouse serum was used to detect metabolite changes; and H&E staining was used to examine orthotopic tumor formation and lung metastasis.

To investigate the effect of cholesterol on HCC metastasis, orthotopic model mice were fed a normal diet (ND) or 2% high‐cholesterol diet (HCD) (D12492, Research Diets, New Brunswick, NJ). To further confirm whether a cholesterol inhibitor (MβCD) could reverse the metastasis‐promoting effects of cholesterol in vivo, HCD‐fed mice were injected intraperitoneally (i.p.) with MβCD (10 mg/kg) every two days. Tumor volumes and metastatic nodules were monitored after six weeks of treatment. Serum glucose and lipids were measured by using an autoanalyzer (IDEXX Catalyst, USA). For the glucose tolerance test, an intraperitoneal glucose tolerance test (IPGTT) was performed. Mice were fasted overnight without food or feces. Then, the mice were injected intraperitoneally with 20% glucose solution (2 g/kg). Blood from the tail vein was taken before and at 15, 30, 60 and 120 min after glucose injection, and blood glucose was measured by a glucometer.

To elucidate the antitumor effects of PLGA‐siRPL6/NPs in vivo, an orthotopic mouse model was created as described above. Two weeks after tumor implantation, PBS, blank NPs, free siRPL6 or PLGA‐siRPL6/NPs at a dose of 2.5 mg/kg were injected into the mice via the tail vein every two days. Then, the weight and volume of the tumors and the number of pulmonary tumors were measured until six weeks after injection.

### RNA‐Seq Analysis for Identifying Critical Tipping Points and Dynamic Network Biomarkers of the Initiation of Metastasis

In this study, the DNB method was applied to identify the critical state and key DNB molecules involved in the initiation of HCC metastasis. DNBs satisfied the following three criteria: 1) the standard deviation (SD_in_) of the dominant group of molecules significantly increased; SD_in_ represents the average standard deviation of all genes in the DNB group; 2) the Pearson's correlation coefficient (PCC_in_) of this dominant group of molecules (expression level) significantly increased; PCC_in_ represents the average Pearson's correlation coefficient of all gene pairs in the DNB group (absolute value); and 3) the Pearson correlation coefficient between this dominant group of molecules and other groups (PCC_out_) rapidly decreased; and PCC_out_ represents the average Pearson correlation coefficient between this dominant group of molecules and other groups (absolute value). The quantitative indicator CI (criticality index) is defined by the combination of std, Inpcc, and Outpcc, which can be used as a numerical signal for the DNB method. When the CI peaks at each period, the gene network is at a critical period or tipping point (CI = $\sqrt{\mathrm{size}}\frac{\mathrm{PCCin}}{\mathrm{PCCout}}$ *SD_in_*, size is the number of DNB genes).

Based on the above model, we collected HCC tissues from a pulmonary metastasis HCC mouse model at a series of time points (W2, W3, W4, and W5; n = 3) for RNA sequencing (RNA‐seq). To identify the DEGs, we compared the intensity of gene expression among the samples at various time points (W3/W2, W4/W2, and W5/W2) using the Limma R package. Subsequently, we used the Tcseq package to perform gene expression pattern clustering analysis of DEGs combined with the R package BioTip (which encapsulates the DNB algorithm) to identify 142 key DNB genes at the critical tipping point (W4) and performed PPI network interoperability analysis through the STRING database to obtain 1895 interoperating DEGs with DNB genes; this group of DEGs was referred to as the DNB‐neighbor DEGs. Furthermore, we aggregated the DNB‐neighbor DEGs before the biological tipping point (W3/W2, W4/W2) and the metastasis‐associated genes in the HCMDB database, obtaining 320 metastasis‐specific DNB‐neighbor DEGs. Then, the DNB genes were analyzed for correlations with metastasis‐specific DNB‐neighbor DEGs, and the numbers of gene pairs with high correlations were counted (abs(cor)>0.8, and *P* < 0.05). For further functional analysis of the core DNB genes, we combined 163 KEGG‐annotated metastasis‐associated signaling pathways reported in previous research,^[^ ^7^ ^]^ and the correlation between the DNB genes and KEGG pathways was analyzed by GSVA. Abs(cor)>0.5, *P* < 0.05 was considered to indicate a significant correlation. To construct a network showing the dysregulation of DNB genes and metastasis‐associated genes, we determined the correlation between the expression levels of target DNB genes and metastasis‐associated genes. |Pearson correlation coefficient|>0.7 and *P* <0.05 were considered to indicate statistical significance.

### Patient Tissues

Human primary HCC tissues and adjacent nontumor (NT) tissues (24 MFH and 24 EHMH samples) were surgically resected from patients at the First Affiliated Hospital of Chongqing Medical University (Chongqing, China). The adjacent NT tissues were at least 2 cm away from the matched HCC tissues. Fresh specimens were immediately frozen in liquid nitrogen and stored at ‐80 °C for analysis. Tumors were homogenized in TRIzol (for quantitative real‐time PCR) or whole‐cell elution buffer (for western blotting) and analyzed for targeted gene mRNA and protein expression. For immunohistochemistry, the samples were fixed in formalin and embedded in paraffin. The use of human samples was approved by the Ethics Committee of Chongqing Medical University (Approval Number: 2 023 046). Informed consent was obtained from each patient involved in the study.

### Quantitative Real‐Time PCR (qPCR)

Total RNA was extracted using TRIzol reagent (Tiangen, China) following the manufacturer's instructions, and cDNA was synthesized using an RT Reagent Kit (Tiangen, China). Then, quantitative real‐time PCR was carried out with SYBR Green with specific primers. The following cycling parameters were used: 95 °C for 3 min, 95 °C for 15 s, 60 °C for 15 s, and 72 °C for 20 s for 40 cycles, with an extension step of 72 °C for 5 min. The 2^‐∆∆Ct^ method was used to determine relative fold changes in target gene expression. β‐Actin was used as an internal control for normalization. All experiments were performed in duplicate. The sequences of primers used are listed in Table S4 (Supporting Information).

### Western Blot

Total protein was extracted from cells or tissues using RIPA buffer supplemented with protease inhibitor cocktail (0 469 313 2001, Roche). Protein concentrations were determined with a BCA protein assay kit (23 227, Thermo, USA). The samples were separated by SDS‐PAGE and transferred onto PVDF membranes (Merck Millipore, USA). The membranes were incubated in 5% nonfat milk in TBST for 2 h at room temperature to block nonspecific binding sites, after which the membranes were incubated with the indicated primary antibodies at 4 °C overnight. After being washed with TBST 3 times, the membranes were incubated with species‐specific HRP‐conjugated secondary antibodies followed by incubation in ECL reagent (WBKLS0500, Millipore, Massachusetts, USA). The protein levels were first normalized to those of GAPDH and subsequently to those of the experimental controls.

### Immunohistochemistry

IHC was performed on 4‐µm‐thick paraffin‐embedded sections. Tissue specimens were deparaffinized in xylene and rehydrated in decreasing concentrations of ethanol, followed by antigen retrieval with sodium citrate buffer. Then, endogenous peroxidase activity was blocked with 3% H_2_O_2_ for 10 min at room temperature. The sections were blocked with 10% normal goat serum for 1 h at room temperature and incubated with specific primary antibodies overnight at 4 °C. The slides were incubated with horseradish peroxidase (HRP)‐conjugated secondary antibody at RT for 30 min, developed in 3,3′‐diaminobenzidine substrate under a microscope, and counterstained with hematoxylin. The immunohistochemical staining scores were calculated by multiplying the staining intensity (0, negative; 1, weak; 2, moderate; 3, strong) by the percentage of positive staining (0, 0%; 1, ≤10%; 2, 10–50%; 3, ≥50%). Images were taken with a Pannoramic MIDI Slide scanner (3D HISTECH, Hungary).

### Lentivirus Transduction and Cell Line Construction

Lentiviruses were produced by transfecting HEK293FT packaging cells with lentiviral transducing plasmid (10 µg), the psPAX2 packaging plasmid (12 260, Addgene, 5 µg), and the pMD2. G envelope plasmid (12 259, Addgene, 7.5 µg) using Lipofectamine 3000 transfection reagent (Invitrogen). Lentiviral supernatants were collected 2 days after transfection and filtered through a 0.45‐µm filter. HCC cells were transduced with viral supernatants supplemented with 8 µg/mL polystyrene and screened with puromycin for 7–14 days.

For the construction of RPL6‐knockout HCC cell lines, gene‐specific sgRNA oligos of RPL6 were cloned and inserted into the lentiCRISPR v2 vector (52 961, Addgene) using the CRISPR/Cas9 system. RPL6 sgRNA was designed by using the CRISPR Design Tool (http://crispr.mit.edu/) and ligated into the lentiCRISPR V2 vector at the BSMBI digestion site. RPL6 protein expression in CRISPR‐knockout experiments was evaluated by western blot after 14 days of selection with puromycin, and TA cloning was used to confirm the knockout efficiency.

For the construction of RPL6‐ or HMGCS1‐overexpressing HCC cell lines, RPL6 or HMGCS1 lentiviral expression constructs were prepared using standard molecular cloning techniques with the pCDH‐EF1‐copGFP‐T2A‐Puro vector (72 263, Addgene). Amplified fragments and the empty pCDH‐EF1‐copGFP‐T2A‐Puro vector were digested with EcoRI (R3101S, NEB) and BamHI (R3136S, NEB), respectively. Vectors expressing target genes were transfected into 293FT packaging cells to produce virus‐containing supernatants for HCC cell transduction, followed by screening with puromycin for 14 days.

To establish RPL6‐ or HMGCS1‐knockdown HCC cells, lentivirus‐driven short hairpin RNAs (shRNAs) were purchased from GeneChem Co., Ltd. (Shanghai, China). Gene silencing efficiency was determined by western blot and qRT‒PCR.

### Cell Proliferation and Colony Formation Assay

Cell proliferation was assessed by CCK‐8 assay kits (HY‐K0301, MCE) in different HCC cell lines. Briefly, cells subjected to different treatments suspended in 200 µL of medium were seeded at 2000 cells per well in 96‐well plates. After incubation, 10 µL of CCK8 solution in 100 µL of medium was added to each well, following incubation for 1, 2, 3, or 4 days, after which the cells were incubated at 37 °C for 2 h. The absorbance at 450 nm was measured using a microplate reader (Epoch, BioTek).

For the colony formation assay, treated cells were suspended in growth medium at a density of 1000 cells/mL. Then, 2 mL of cell suspension medium was added to 6‐well plates. After incubation at 37 °C for 2–3 weeks, the cells were washed with PBS twice and fixed with 4% paraformaldehyde before staining with crystal violet. Colonies with diameters greater than 1.0 mm were counted and statistically analyzed.

### Invasion and Migration Assay

For the transwell assay, after incubation in 5 µg/mL mitomycin‐C for 2 h, 1 × 10^6^ cells resuspended in DMEM were seeded in the upper transwell chamber, which had an 8 µm diameter pore membrane coated with (migration assay) or without (invasion assay) Matrigel (356 234, Corning), and the lower chamber was filled with 0.7 mLof complete DMEM (10% FBS, 1% penicillin‒streptomycin solution). After incubation for 24 h at 37 °C, the cells were fixed in 4% paraformaldehyde and stained with 0.1% crystal violet. Then, the membranes were fixed to slides with neutral resin, and the migrated cells were counted under a microscope.

For the wound‐healing assay, 1 × 10^6^ cells were seeded in 6‐well plates and incubated until they reached 80% confluence. Then, the cells were treated with 5 µg/mL mitomycin‐C for 2 h, and the monolayer was disrupted with a pipette. The suspended cells were washed with PBS, and 2 mL of complete growth medium was added to the wells. Images were taken at 0 and 48 h under a phase‐contrast microscope. The migration rate was calculated as the wound width.

To assess the effect of cholesterol on the migratory and invasion of HCC cells, the cells were treated with cholesterol (10 µM) (C4951, Sigma) for 24 h, and for methyl‐β‐cyclodextrin (MβCD) treatment, the cells were incubated with complete medium supplemented with MβCD (5 mM) (C4555, Sigma) for 2 h and then washed with PBS twice, followed by the indicated experiments.

### Gelatin Invadopodia Assay

The QCM gelatin invadopodia assay (ECM671, Sigma) was performed according to the manufacturer's protocol to assess the ability of HCC cells to form invadopodia and degrade the matrix. Briefly, slides were treated with polylysine (room temperature, 20 min) and then fixed with glutaraldehyde solution (room temperature, 15 min). After incubation with a prewarmed fluorescent gelatin mixture (Cy3‐gelatin/unlabeled gelatin 1:5, incubated at room temperature for 15 min), the slides were then sterilized with 70% ethanol (room temperature, 30 min), and cell cultures were prepared in DMEM (room temperature, 30 min) to quench free aldehydes. HCC cells subjected to the indicated treatments were seeded on the gelatin‐coated slides and incubated in the dark for 48 h. After the cells were fixed with 4% paraformaldehyde, they were stained with FITC‐phalloidin and DAPI to visualize the colocalization of gelatin degradation and cellular features. The samples were observed using confocal microscopy (Leica, Wetzlar, Germany), and images were captured. The localized degradation of the area colocalized with invadopodia and gelatin was used to assess the matrix degradation ability of the invadopodia, and the degradation of the gelatin area was determined by using ImageJ.

### F‐Actin Staining

Cells were seeded on slides in 12‐well plates, and after the indicated treatments, the cells were washed 3 times with PBS, fixed with 4% paraformaldehyde for 15 min and permeabilized with 0.5% Triton‐100 for 10 min. Then, the cells were stained with DyLight 488 phalloidin (1:40) for 10 min at room temperature to visualize F‐actin, and the nuclei were stained with DAPI for 20 min in the dark. Then, the sections were sealed with an anti‐fluorescence quenching agent, and images were captured by confocal microscopy (Leica, Wetzlar, Germany). The number of cells with lamellipodia was counted by ImageJ software.

### Hepatocyte‐Specific RPL6 Knockout Mice

Hepatocyte‐specific RPL6 knockout mice were used. RPL6^flox/flox^ (Flox) and Alb‐Cre mice were purchased from Shanghai Biomodel Organism Science & Technology Development Co., Ltd. (Shanghai, China). Hepatocyte‐specific RPL6 knockout (RPL6^HKO^) mice were generated by crossing RPL6^flox/flox^ (Flox) mice with Alb‐Cre mice.

For the DEN‐induced hepatocarcinogenesis model, 4‐ to 6‐week‐old RPL6^HKO^ mice and RPL6^flox/flox^ control littermates were injected intraperitoneally with DEN (25 mg/kg body weight, N0756, Sigma‒Aldrich,), and then, the mice were injected weekly with CCl_4_ (1 mL/kg body weight, mixed in olive oil at a ratio of 1:9, 488 488, Sigma‒Aldrich) starting 4 weeks after the initial DEN injection. Ten months later, the mice were sacrificed, and liver and lung tissues were separated into individual lobes to count tumor nodules on the tissue surface. The tissues were histopathologically analyzed via histological hematoxylin‐eosin (H&E) staining after fixation with 4% paraformaldehyde.

To study the effect of HMGCS1 on HCC metastasis, RPL6^HKO^ mice treated with DEN for 10 months were injected with AAV8‐GFP or AAV8‐HMGCS1 (1 × 10^11^ viral genomes in 200 µL of saline) via the tail vein. Eight weeks after the injection, the mice were euthanized, and tumor growth in the liver and lungs was collected and quantified. The animal studies were performed in strict accordance with the Animal Research: Reporting of In Vivo Experiments (ARRIVE) guidelines, and all the experimental procedures were approved by the Experimental Ethics Committee of Chongqing Medical University (IACUC‐CQMU‐2023‐0382).

### RNA Immunoprecipitation (RIP)

RNA immunoprecipitation was performed with the Magna RIPTM RNA‐Binding Protein Immunoprecipitation Kit (17‐701, Millipore) according to the manufacturer's instructions. Briefly, 1) HCC cells were transfected with a FLAG‐tagged plasmid expressing the RPL6 gene or an untagged plasmid (vector) for 48 h, collected from a 10 cm dish at 90% confluence via cell scrapers, washed twice with cold PBS, lysed in 200 µL of complete RIP Lysis Buffer on ice for 5 min and centrifuged at 12,000 rpm for 30 min. Then, the supernatants were collected and stored at −80 °C. 2) For the preparation of magnetic beads, 50 µL of magnetic beads was transferred to each tube, the beads were washed with 100 µL of RIP Wash Buffer and then resuspended in 100 µL of RIP Wash Buffer. Then, 5 µg of the indicated antibodies was added and incubated with the resuspended beads for 30 min at room temperature. 3) The cell lysates (from step 1) were quickly thawed and centrifuged at 14 000 rpm for 10 min at 4 °C. Then, 100 µL of RIP lysate was removed and added to the bead‐antibody complex, which was subsequently resuspended in 900 µL of RIP immunoprecipitation buffer (from step 2). Then, 10 µL of the supernatant of the RIP lysates was removed, and the proteins were stored as input samples. Then, the RIP immunoprecipitation mixture was incubated at 4 °C overnight. 4) For the purification and analysis of RNA, associated RNA‒protein complexes were collected and washed 6 times with RIP Wash Buffer, subjected to proteinase K digestion, and isolated with phenol, chloroform, and isoamyl alcohol. The relative interaction between protein and RNA was detected by real‐time PCR and normalized to the input and negative IgG controls.

### Dual‐Luciferase Assay

The 5′UTR, coding sequence (CDS), 3′UTR and other fragments of HMGCS1 mRNA with the KpnI or SacI restriction enzyme digestion site were amplified, cloned and inserted into a dual‐luciferase pGL3‐derived reporter vector. The 4xHRE‐luciferase reporter plasmid was purchased from Youbao Biotechnology (China), and the ODD‐luciferase reporter plasmid consisting of the hydroxyl‐dependent degradation region of HIF‐1α was purchased from Addgene (18 965). HCC cells were seeded in 12‐well plates and transfected with the luciferase vectors and Renilla vector as loading controls. After transfection for 24 h, the cells were analyzed for luciferase levels with the Dual‐Luciferase Reporter Assay System (E1910, Promega, USA) according to the manufacturer's instructions. The firefly luciferase activity values were determined by a GloMax microplate luminometer, and the transfection efficiencies were normalized to the Renilla luciferase activity.

### Nascent RNA Synthesis Assay

RPL6‐knockdown or RPL6‐overexpressing HCC cells were incubated with 0.5 mM 5‐ethynyl uridine (5‐EU) for 2 h before harvesting. The newly synthesized EU‐labeled RNA was purified from total RNA, and the EU‐labeled HMGCS1 RNAs were quantified by RT‒qPCR following the protocols of the Click‐iT Nascent RNA Capture Kit (MP10365, Thermo).

### RNA Stability

To measure RNA stability in HCC cell lines, 5 µg/mL actinomycin D (Act. D, S8964, Selleck) was added to the HCC cells. The cells were incubated in 6‐well plates and collected after treatment with Act. D at the indicated times. Then, RNA was isolated for qRT‒PCR, and β‐actin was used as an internal control for normalization.

### RNA Pull‐Down Assay

The HMGCS1 RNA probes (50 pmol) were transcribed in vitro using the T7 In Vitro Transcription Kit, and then, biotin RNA was used to label HMGCS1 mRNA and its truncations according to the manufacturer's protocol (20 163, Thermo). Then, 100 pmol of biotinylated RNA was chemically coupled to streptavidin‐conjugated magnetic beads (50 µL) for 2 h at room temperature (20 164, Thermo). Then, 500 µg of total protein extracted from Huh7 cells was added to the RNA‐loaded beads, and the mixture was incubated at 4 °C overnight. The beads were briefly washed three times with binding wash buffer. Precipitated proteins were eluted with 40 µl of protein lysis buffer. Proteins were detected by SDS‒PAGE. The truncated HMGCS1 3′UTR primers used for in vitro transcription are shown in Table S4 (Supporting Information).

### RNA Electrophoretic Mobility Shift Assay (REMSA)

Biotin‐labeled RNA probes for HMGCS1 mRNA were prepared as described for the RNA pull‐down assay. Protein‒mRNA complexes were separated by REMSA on 8% polyacrylamide gels for 45 min and then transferred to nylon membranes for further analysis. The validation of protein‒mRNA interactions was performed using the LightShift Chemiluminescent RNA EMSA Kit (Thermo Fisher Scientific, Inc.) according to the manufacturer's protocol.

### Untargeted Lipidomics Analysis

For samples of cultured cells, 1 × 10^7^ HCC cells subjected to the indicated treatments (n = 4) were harvested using a cell scraper and washed three times with cold PBS. Then, the cells were homogenized in 450 µl of methanol/Milli‐Q H_2_O (v/v = 1:1) and centrifuged at 14000 × g for 10 min at 4 °C. The upper organic phase was transferred to a new tube and dried with nitrogen. For serum samples, 100 µL of mouse serum was collected (n = 5) and subjected to lipid extraction using MBHT extraction solvent. All the samples were dissolved in 200 µl of isopropanol/methanol solution (v/v = 1:1) for mass spectrometry analysis. ESI‐positive and ESI‐negative ion modes were used for the detection of metabolites. The samples were analyzed by using an HPLC system (1260 series, Agilent Technologies) and mass spectrometer (Agilent 6460, Agilent Technologies), and the data analysis was supported by Shanghai Applied Protein Technology Co., Ltd.

### Cholesterol Concentration Assay

Cellular cholesterol levels were measured with an Amplex Red Cholesterol Assay Kit (A12216, Invitrogen) according to the manufacturer's instructions. In brief, HCC cells subjected to the indicated treatments were plated in 6 cm dishes, and cells were harvested, washed in cold PBS, centrifuged, and extracted using chloroform‐methanol (v/v, 2:1) in a microhomogenizer. Then, the cholesterol concentrations were evaluated according to the manufacturer's instructions. A cholesterol standard curve was determined using a cholesterol standard diluted at various concentrations in reaction buffer. The amount of cholesterol ester was calculated by subtracting the amount of free cholesterol from the amount of total cholesterol. The cholesterol concentration in serum or tissues was also measured using a cholesterol assay kit according to the manufacturer's instructions.

The free cholesterol content in cells was analyzed by filipin III staining. Briefly, HCC cells subjected to the indicated treatments were seeded on slides in 12‐well plates at a density ranging from 50 to 60%. Then, the cells were harvested, fixed with 4% paraformaldehyde, and incubated with 0.05 mg/mL filipin III (HY‐N6718, MCE) for 4 h at room temperature. After the cells were washed three times with PBS, the nuclei were stained with SYTOX Deep Red (P36990, Invitrogen), and the cell slides were sealed. Filipin III staining of the cells was observed using a Leica confocal microscope (excitation wavelength of 340–380 nm, emission wavelength of 385–470 nm). Filipin staining was quantified using ImageJ software. For flow cytometry analysis, the treated cells were harvested and fixed with 4% paraformaldehyde for 15 min at room temperature, stained with 0.05 mg/mL filipin III for 30 min at room temperature, and then analyzed by flow cytometry after being washed three times with wash buffer. The fluorescence intensity was analyzed by FlowJo 10 software. For the LC‒MS analysis of cholesterol, serum samples from HCC patients were prepared by extraction, hydrolysis, derivatization, and washing; the methods for the identification and quantification of the substances were published previously.^[^ ^54^ ^]^

### In Vitro Isotopic Tracing of Cholesterol Biosynthesis

To measure cholesterol biosynthesis, HCC cells were cultured for 24 h in growth medium (tracer media) with 10% LPDS, 25 mM [U^13^C]‐glucose, and 4 mM glutamine. After washing with cold PBS three times, cells were then lysed with EDTA‐trypsin, and cell lysates were collected. The samples were adjusted to aliquots according to total protein level. Sterols were extracted from cells using a modified version of Bligh and Dyer's protocol. Lipid extract was resuspended in 500 µL of ethanol containing 5 µg of butylated hydroxytoluene (BHT). An internal standard cocktail (50 µL) comprising ergosterol (Macklin) was added to the samples. The samples were incubated at 1200 rpm for 15 min at 4 oC. At the end of incubation, 250 µL of MilliQ water and 1 mL of n‐hexane were added. The samples were mixed thoroughly by vortexing and centrifuged at 12 000 rpm for 5 min 4 °C. The Clear upper phase containing oxysterols and sterols in hexane was transferred to a new tube. The extraction was repeated once with another 1 ml of n‐hexane. The pooled extract was dried in a SpeedVac under organic mode. Oxysterols were derivatised to obtain their picolinic acid esters prior to LC/MS analysis on a Shimadzu 40 × 3B‐UPLC coupled to Sciex QTRAP 6500 Plus, and quantitated by referencing to the spiked internal standards as previously described.^[^ ^55^ ^]^ Labeling on cholesterol is depicted as ^13^C mole percent enrichment (MPE) from [^13^C]‐glucose relative to the control condition. Cholesterol analysis was conducted at LipidALL Technologies. Source data were provided in Table S5 (Supporting Information).

### Proteomics Analysis

Huh7 cells or RPL6‐knockout MHCC97H were treated with cholesterol (10 µM) for 24 h at 37 °C, harvested, and sonicated 3 times on ice in lysis buffer. Then, the protein concentration was determined with a BCA kit. The protein solution was reduced with 5 mM dithiothreitol for 30 min at 56 °C and alkylated with 11 mM iodoacetamide for 15 min at room temperature in the dark. Then, the protein sample was diluted by adding 100 mM TEAB to a urea concentration less than 2 M and processed using a TMT kit according to the manufacturer's protocol. The released peptides were subjected to LC‒MS/MS identification and quantification on a mass spectrometer (Thermo Fisher Scientific) with a Finnigan Nanospray II electrospray ionization source. The resulting MS/MS data were processed using the MaxQuant search engine v.1.5.2.8 (www.maxquant.org), and tandem mass spectra were searched against the human UniProt database (http://www.ebi.ac.uk/UniProt/), which was concatenated with a reverse decoy database. Based on the differentially expressed proteins that were identified (those with changes in the protein ratio >1.5, *P* value <0.05), KEGG pathway enrichment analysis was conducted with the KEGG database, and *P* value <0.05 (Fisher's exact test) was considered to indicate statistical significance. Protein‒protein interactions were analyzed using the STRING database, and PPI networks from STRING were visualized using the Cytoscape software. The quantitative proteomic analysis of Huh7 cells was carried out by Jingjie PTM Biolabs Inc. (Hangzhou, China).

### Microscale Thermophoresis Analysis

The interaction between HIF‐1α and cholesterol was measured by using a Monolith NT.115 MST (NanoTemper, Germany) at room temperature. The purified His‐tagged HIF‐1α protein or His‐tagged HIF‐1α^Q352A/K388A^ mutation was diluted with MST buffer to a concentration of 20 nM and labeled with the Monolith His‐Tag Labeling Kit RED‐tris‐NTA 2nd Generation (MO‐L018, NanoTemper). Then, the labeled HIF‐1α (10 µL) was mixed and incubated with cholesterol (10 µL) at different concentrations in the dark for 30 min at room temperature. Subsequently, the mixture was loaded into premium capillaries (NanoTemper Technologies), and the MST measurements were performed at 25 °C and at medium MST power. The Kd values were calculated using the mass action equation available in NanoTemper software (MO. affinity Analysis).

### Coimmunoprecipitation Assay

HCC cells were lysed with RIPA buffer for 20 min and then centrifuged at 4 °C for 10 min. After quantifying the protein concentration, the proteins were removed with protein A/G magnetic beads (88 803, Thermo) for 60 min at 4 °C, and 500 µg of total protein was immunoprecipitated with HIF‐1α (36169S, CST) and precipitated overnight at 4 °C. The negative control rabbit IgG (3900S, CST) was coupled to protein A/G magnetic beads overnight at 4 °C. The immunoprecipitates were washed three times with RIPA lysis buffer and eluted in SDS‒PAGE loading buffer. Then, the supernatants were boiled at 95 °C for 10 min for collection for western blotting.

### Preparation of siRPL6‐Encapsulated Nanoparticles

siRPL6‐encapsulated nanoparticles were prepared using a double emulsion solvent evaporation method as previously described.^[^ ^56^ ^]^ Briefly, 0.2 mg of siRNA was dissolved in 25 µL of ribonuclease‐free H_2_O, and the resulting solution was mixed with 0.5 mL of chloroform containing 25 mg of PEG‐PLGA and 3 mg of DOTAP. The mixture was homogenized using an ultrasonic probe (60% amplitude) on ice for 1 min. The resulting solution was mixed with 5 mL of ribonuclease‐free H_2_O and sonicated (60% amplitude) for 1 min. Finally, the chloroform was removed via rotary evaporation at 37 °C using a rotary evaporator. The NPs were collected by centrifugation at 14 000 rpm for 30 min at 4 °C. Instead of siRNA, cy5‐siRNA was used for preparing fluorescence‐labeled nanoparticles using a previously described method. The particle size and zeta potential were determined using a Malvern Zetasizer Nano ZS unit (Nano ZS90, Malvern, UK). The NP morphologies were characterized using TEM at an accelerating voltage of 200 kV.

### Cellular Uptake of PLGA‐siRNA/NPs

To verify the cellular uptake of siRNA, MHCC97H cells were seeded on coverslips in 12‐well plates and cultured for 24 h until they reached 50–60% confluency. Then, the cells were incubated with PLGA‐Cy5‐siRNA/NPs in DMEM at 37 °C for 2 h. PBS, blank NPs, and free siRNA were used as controls. HCC cells were washed with precooled PBS and fixed in 4% paraformaldehyde solution for 15 min. The nuclei were stained with DAPI, and the cellular uptake of PLGA‐siRNA/NPs was visualized by confocal microscopy.

### Patient‐Derived Xenograft (PDX) Model

PDX mouse model was obtained from the BEIJING IDMO Co., Ltd. To construct a patient‐derived xenograft (PDX) model, fresh HCC tissues obtained from patients were cut into small pieces (1–3 mm^3^) immediately and directly implanted subcutaneously into 6‐week‐old male NOD/SCID IL2rg^‐/‐^ (NSG) mice. When the tumors reached an appropriate volume (400‐600 mm^3^), they were divided into 3 mm^3^ pieces, which were orthotopically implanted into the left lateral lobe of the liver in NSG mice. The orthotopically implanted mice were randomized into groups (n = 5 per group). When the tumors had grown to an appropriate volume at the fourth week, the cells were treated with PLGA‐siRPL6/NPs or PLGA‐siNC/NPs (2.5 mg/kg every 2 days, i.v.) for six weeks. Then, liver tumor volume was measured, and lung metastasized tumor nodules were counted.

### Biodistribution of PLGA‐NPs In Vivo

To evaluate the distribution of PLGA‐NPs, PLGA‐Cy5‐siRNA/NPs were used to reduce the interference of autofluorescence. Orthotopic model mice (6 weeks old) were injected with PBS, free Cy5‐siRNA, or PLGA‐Cy5‐siRNA (200 µL injection of equivalent 40 µg of Cy5‐siRNA per mouse) via the tail vein. One hour post injection, the mice were anesthetized with isoflurane, and fluorescence imaging was performed using an in vivo imaging system (IVIS Spectrum; PerkinElmer, USA).

### Immunofluorescence

HCC cells were seeded on glass coverslips in 12‐well plates and grown to a density of 50–60%. After the indicated treatments, the cells were rinsed in PBS twice, fixed for 15 min with 4% paraformaldehyde, permeabilized with cold PBS supplemented with 0.5% Triton X‐100, and blocked with 5% BSA. The cells were then incubated with the indicated antibodies (1:200 dilution) overnight at 4 °C and with Alexa Fluor 555‐coupled goat anti‐mouse IgG (1:500 dilution) for 1 h at room temperature. Cell nuclei were stained with DAPI solution (1:1000 dilution, Sigma) for 20 min at room temperature, and images were captured with a confocal microscope (Leica, Wetzlar, Germany).

### Multiplex Immunofluorescence Staining

Formalin‐fixed paraffin‐embedded (FFPE) HCC tissue sections were subjected to multiplexed immunofluorescence using a 4‐plex IHC kit (10 079 100 020, Panovue, Beijing, China) according to the manufacturer's instructions, following deparaffinization, rehydration, and epitope retrieval. Primary antibodies against RPL6 (NBP2‐20216, Novus Biologicals, 1:400), HMGCS1 (53‐9003‐82, Invitrogen, 1:400), and HIF‐1α (53‐9003‐82, Invitrogen, 1:400) were used in this experiment. All sections were incubated with horseradish peroxidase‐conjugated secondary antibodies and subjected to tyramide signal amplification. Then, the sections were counterstained with DAPI (D1306, Thermo, 1:1000) for 20 min. The stained sections were imaged with a confocal microscope (Leica, Wetzlar, Germany). Images were quantified using HALO image analysis software (Indica Labs). Six random high‐power fields were used to quantify the ratio of certain cells to the total number of cells.

### Statistical Analysis

The data are presented as means ± SD of at least three independent experiments. All the statistical analyses were conducted using GraphPad Prism 8 (GraphPad) or SPSS 19.0 (SPSS). Statistical analyses and the number of samples (n) were described in detail in each figure. Significance is noted in the figures or figure legends. The data distribution was assumed to be normal, but normality was not formally tested. No statistical methods were used to predetermine sample sizes, but our sample sizes are similar to those reported in previous publications. Comparisons between two groups were performed using two‐tailed unpaired Student's t tests, and when the number of groups was >2, parametric ANOVA was used. Kaplan–Meier curves were used to evaluate the survival of human patients (log‐rank test). The correlation between RPL6 expression and clinicopathological parameters was assessed using the chi‐squared test. *P* <0.05 was considered to indicate statistical significance (*P <0.05; **P <0.01; ***P <0.001; ns: not significant).

## Conflict of Interest

The authors declare no conflict of interest.

## Author Contributions

M.Y., S.C., H.G., and S.Z. contributed equally to this work. J.C. conceived the project and designed the study. M.Y., S.C., D.Z., and P.C. performed the experiments and acquisition of data. J.C. and M.Y., S.C., D.Z., X.H., and H.D. analyzed and interpreted data. S.C., H.G., D.Z., H.X., S.Z., W.C., Z.Z. J.R., M.T., H.Z., F.L., and Y.H. provided the materials and technical support. J.C. and M.Y. wrote the paper and critically reviewed the manuscript. S.C., H.G., and D.Z. revised the manuscript. J.C. provided supervision of the study. All authors read and approved the article.

## Supporting information



Supporting Information

## Data Availability

All data that support the findings of this study are available in the manuscript and the supplementary data file. All unprocessed images of western blots and stained agarose gels data are available in the original data file. The row RNA‐seq data generated in this study have been deposited in Gene Expression Omnibus (GEO) under the accession numbers GSE274579 and GSE274574, and in NCBI BioProject database under the accession number PRJNA1146575. The mass spectrometry proteomics data have been deposited in the ProteomeXchange Consortium via the PRIDE partner repository with the dataset identifiers PXD054740 and PXD064850. The original metabolic profiles have been deposited in Big Sub with the dataset identifiers OMIX010523 and OMIX010599. All other data supporting the findings of this study are available from the corresponding author upon reasonable request.
