# Erratum to “RPL6 Interacts with HMGCS1 to Stabilize HIF‐1α by Promoting Cholesterol Production in Hepatocellular Carcinoma”

**DOI:** 10.1002/advs.202514955

**Published:** 2025-08-28

Yang M, Cheng S, Gu H, Zhong S, Zhang D, He X, Chen W, Deng H, Ren J, Chen P, Tan M, Zhang H, Li F, Zhang Z, Hu Y, Chen J. RPL6 Interacts with HMGCS1 to Stabilize HIF‐1α by Promoting Cholesterol Production in Hepatocellular Carcinoma. Adv Sci (Weinh). 2025 Jul 12:e01373. doi: 10.1002/advs.202501373. Epub ahead of print. PMID: 40650669.

In the originally published article, there are errors in Figures S12b,f and S13b (Supporting Information). The images for the migratory transwell assay of Huh7 cells in Figure S12b (Supporting Information) (Cho+MβCD group) and Figure S10b (Supporting Information) (Vector group), the gelatin degradation assay of MHCC97H cells in Figure S13b (Supporting Information) (shCont group) and Figure 3c (RPL6 KO2 group) were inadvertently duplicated during images processing. In Figure S12f (Supporting Information), the image of the invasive transwell assay (RPL6 group) was mistakenly distorted during image exporting and selection. The correct Figures S12 and S13 (Supporting Information) are reproduced below. As the quantification analysis was based on the correct data, these errors do not affect the results or conclusions of this article. The authors apologize for any inconvenience this may have caused.


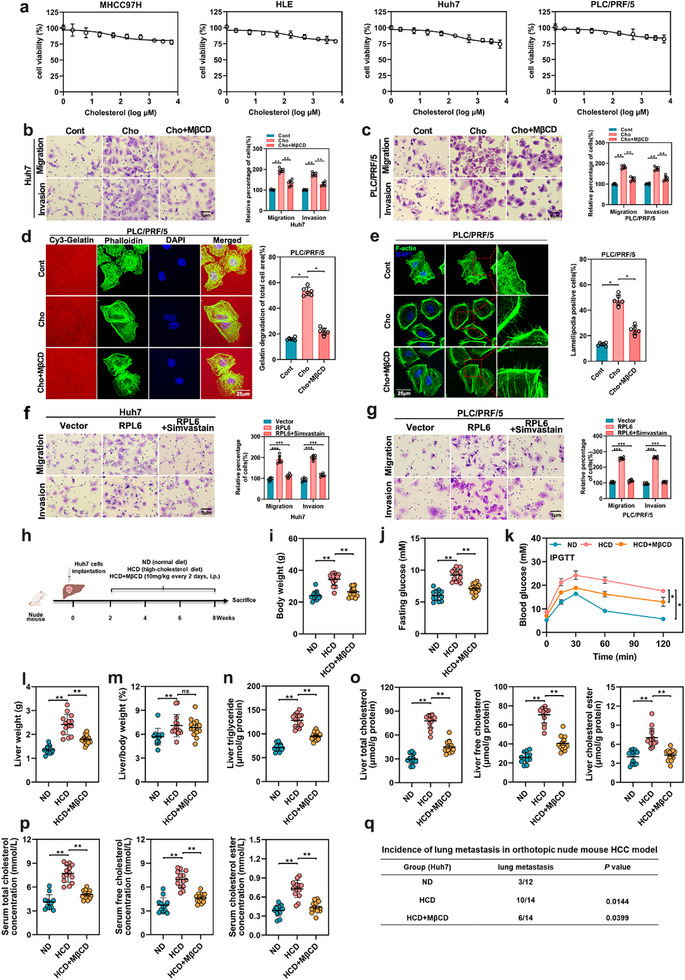


Figure S12. The effect of cholesterol on HCC cell migration and invasion in vitro and metastasis in vivo. a) MHCC97H, HLE, Huh7 and PLC/PRF/5 cells were treated with various concentrations of cholesterol for 72 h. The effects of cholesterol on cell viability were determined by the MTT assay. b,c) The effect of cholesterol (10 µM) and the addition of methyl‐b‐cyclodextrin (MβCD) on migration and invasion function in Huh7 (b) and PLC/PRF/5 (c) cells was tested by transwell assays. The cells were counted from 6 images. d) The effect of cholesterol (10 µM) and the addition of methyl‐b‐cyclodextrin (MβCD) on invadopodia function in Huh7 and PLC/PRF/5 cells was tested by gelatin degradation assay. Images were obtained by confocal, and the degraded areas of at least six fields were quantified using image J software. Scale bar, 25 µm. e) The effect of cholesterol (10 µM) and the addition of methyl‐b‐cyclodextrin (MβCD) on lamellipodia formation in PLC/PRF/5 cells was evaluated through F‐actin staining. Representative images were shown, and the percentage of cells with lamellipodia formation was counted from at least six regions. Scale bar, 25 µm. f,g) The effect of RPL6 and simvastatin (2 µM) treatment on migration and invasion function in Huh7 (f) and PLC/PRF/5 (g) cells was tested by transwell assays. The cells were counted from 6 images. h) Illustration of the HCD‐fed or MβCD therapeutic study. Two weeks after Huh7 cells implantation, the mice were treated with ND or HCD, or MβCD treatment (10 mg kg^−1^ of mouse, i,p) every 2 days for 6 weeks. *n* = 12–14 per group. i–p), Body wight (i), fasting glucose j), glucose tolerance test k), liver weight l), liver‐body weight ratios m), liver triglyceride n), total, free and esterified cholesterol levels of liver tissues o) and serum p) in mice fed with ND, HCD and HCD with MβCD treatment. q) Statistical analysis of orthotopical injection model for lung metastasis events of these mice. Data are shown as mean ± SD of at least three independent experiments (a–g). Statistical significance was determined by two‐tailed unpaired Student's *t*‐test (b–g,i,j,l–q) or one‐way analysis of variance (ANOVA) k). ^*^ *p* <0.05; ^**^ *p* <0.01. ns, not significant.


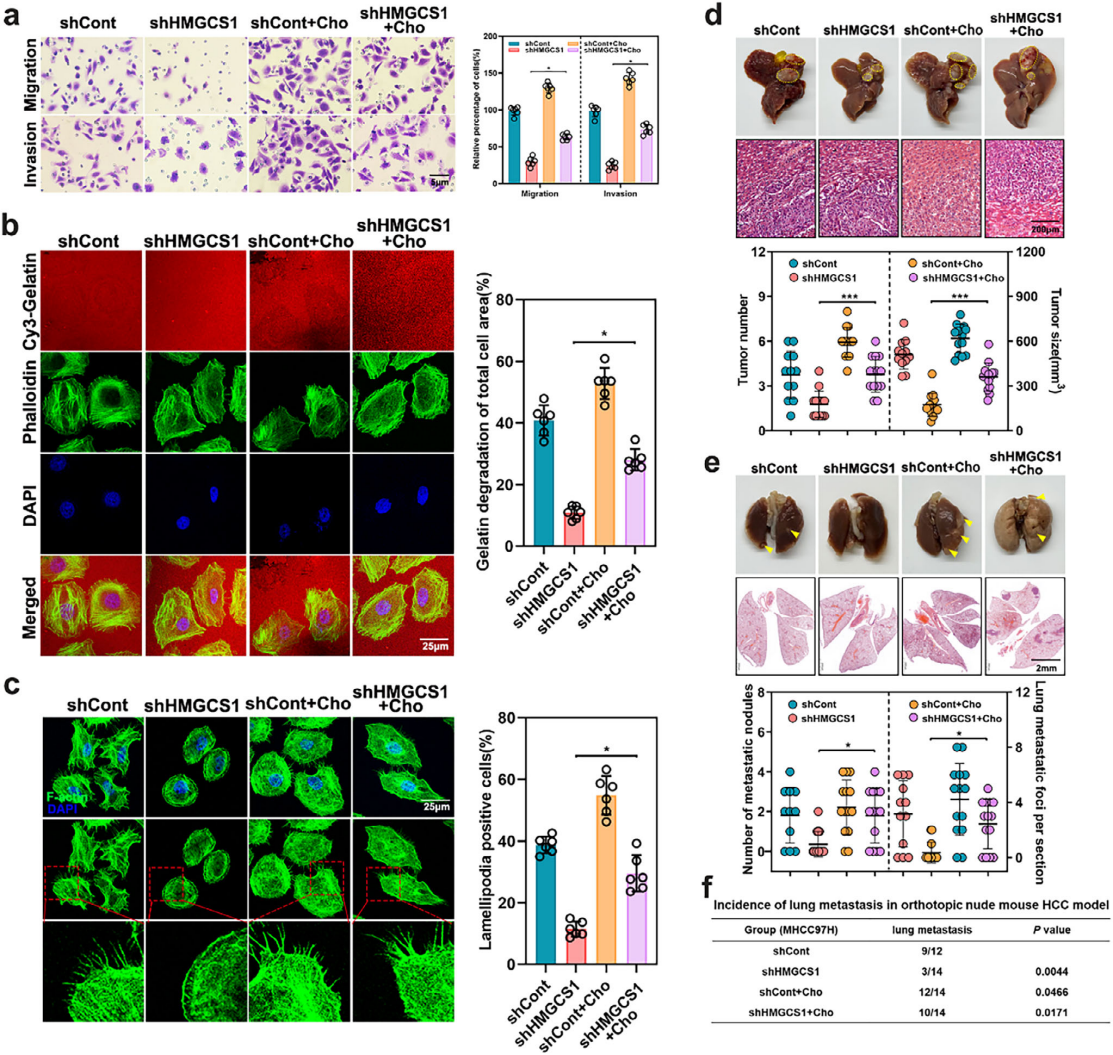


Figure S13. Intracellular cholesterol is involved in the maintenance of HCC migration and invasion in vitro and metastasis in vivo. a) The effect of cholesterol (10 µM) on migration and invasion in HMGCS1 knockdown cells was tested by transwell assays. The cells were counted from 6 images. b) The effect of cholesterol (10 µM) on invadopodia function in HMGCS1‐knockdown cells was determined by gelatin degradation assay. Representative images were captured by confocal, and the degraded areas of at least six fields were quantified using image J software. Scar bar, 25 µm. c) The effect of cholesterol (10 µM) on lamellipodia function in HMGCS1‐knockdown cells was determined by F‐actin staining assay. Representative images were shown, and the percentage of cells with lamellipodia formation was counted. Scar bar, 25 µm. d–f) MHCC97H cells with stable knockdown of HMGCS1 or control cells were orthotopically injected into the left lobe liver of nude mice to establish a lung metastasis model, following the treatments of ND or HCD. Liver and lung tissues collected at 8 weeks post‐injection were used for detection. *n* = 12–14 per group. d) Representative macroscopic images and H&E staining of the tumor‐bearing liver in these mice. The tumor number and tumor size were examined. Scar bar, 200 µm. e) Representative macroscopic images and H&E staining of the tumor‐metastasis lung from these mice. The number of metastatic nodules and metastatic foci was examined. Scar bar, 2 mm. f) Statistical analysis for lung metastasis events of these mice. Data are shown as mean ± SD of at least three independent experiments (a–c). Statistical significance was determined by one‐way analysis of variance (ANOVA). ^*^ *p* <0.05; ^***^ *p* <0.001.

## Supporting information



Supporting Information

