# Supplementary material for: RPL6 Interacts with HMGCS1 to Stabilize HIF‐1α by Promoting Cholesterol Production in Hepatocellular Carcinoma
Source: Adv Sci (Weinh). 2025 Jul 12;12(38):e01373. doi: 10.1002/advs.202501373 (PMC12520468; doi:10.1002/advs.202501373)
Supplement: Supplementary file 1 — Supporting Information [file ADVS-12-e01373-s001.docx]

**Supporting Information**

**RPL6 interacts with HMGCS1 to stabilize HIF-1α by promoting cholesterol production in hepatocellular carcinoma**

Minli Yang^#^, Shengtao Cheng^#^, Huiying Gu^#^, Shan Zhong^#^, Dapeng Zhang, Xin He, Weixian Chen, Haijun Deng, Jihua Ren, Peng Chen, Ming Tan, Hui Zhang, Fan Li, Zhenzhen Zhang^*^, Yuan Hu^*^, Juan Chen^*^

^#^These authors contributed equally to this work.

*Corresponding authors

Correspondence to: chenjuan2014@cqmu.edu.cn; zhangzhenzhen@cqmu.edu.cn; huyuan@cqmu.edu.cn

**Table of contents**

[**Supporting Information** 1](#_Toc201068356)

[**Supporting Fig. 1** 3](#_Toc201068357)

[**Supporting Fig. 2** 4](#_Toc201068358)

[**Supporting Fig. 3** 5](#_Toc201068359)

[**Supporting Fig. 4** 7](#_Toc201068360)

[**Supporting Fig. 5** 9](#_Toc201068361)

[**Supporting Fig. 6** 10](#_Toc201068362)

[**Supporting Fig. 7** 11](#_Toc201068363)

[**Supporting Fig. 8** 13](#_Toc201068364)

[**Supporting Fig. 9** 15](#_Toc201068365)

[**Supporting Fig. 11** 18](#_Toc201068366)

[**Supporting Fig. 12** 20](#_Toc201068367)

[**Supporting Fig. 13** 23](#_Toc201068368)

[**Supporting Fig. 14** 25](#_Toc201068369)

[**Supporting Fig. 15** 28](#_Toc201068370)

[**Supporting Table 1.** 30](#_Toc201068371)

[**Supporting Table 2.** 32](#_Toc201068372)

[**Supporting Table 3.** 33](#_Toc201068373)

[**Supporting Table 4.** 34](#_Toc201068374)

[**Supporting Table 5.** 39](#_Toc201068375)


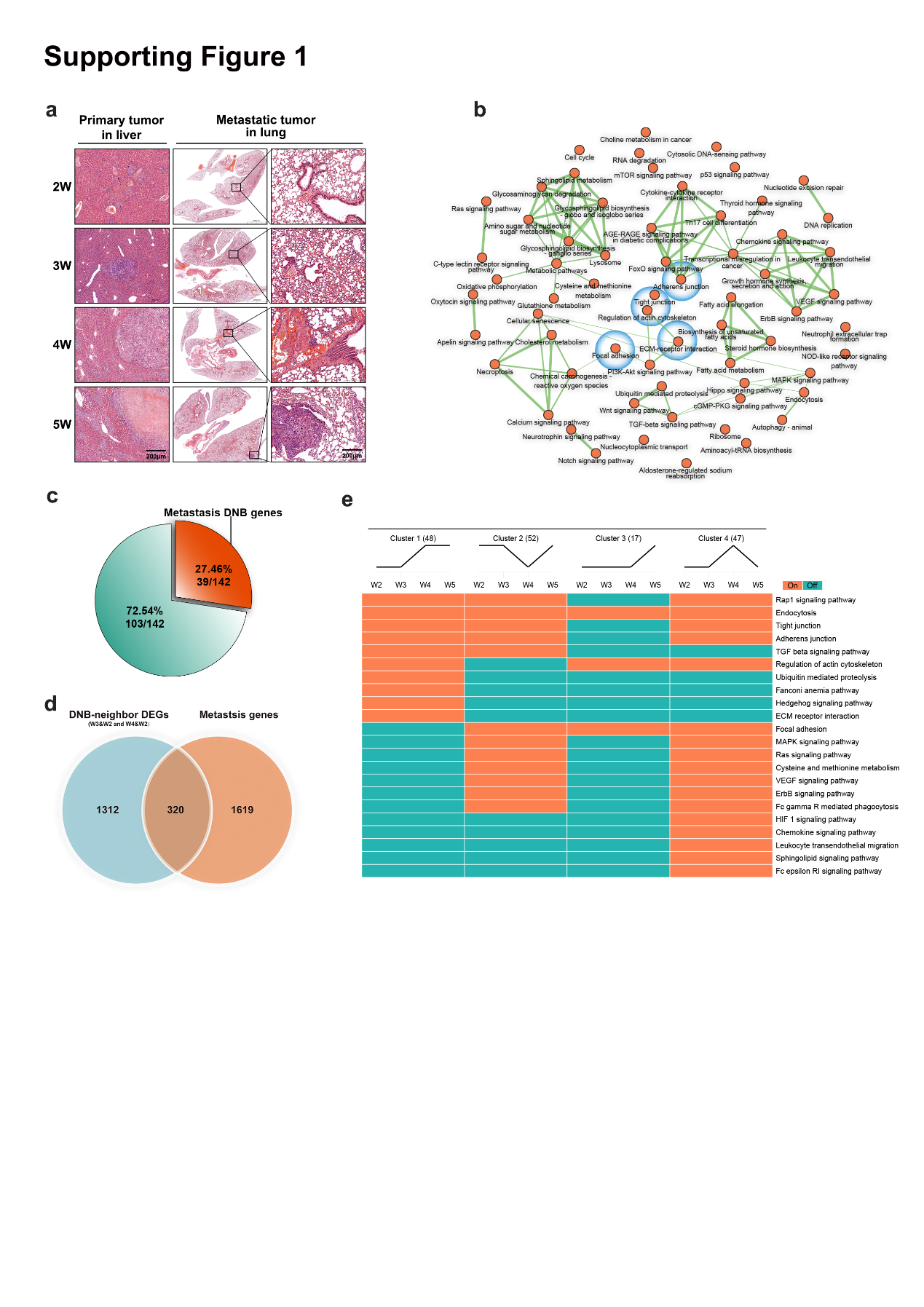


**Supporting Fig. 1 The characteristics of DNBs during the progression of HCC metastasis.**

**a**, Images of orthotopic xenografted mouse model with liver tumors and pulmonary metastasis stained with H&E at different time points. Scar bar, 200 μm. **b**, A network diagram illustrating the pathways associated with DNB genes. **c**, The proportion of DNB genes involved in tumor metastasis-related pathways by Venn diagram. **d**, The intersection between DNB-neighbor DEGs and metastasis genes visualized by using Venn diagram. **e**, The heatmap showing the dynamic activity of the associated KEGG pathway involved in RPL6 and tis neighboring DEGs in different dynamic patterns.


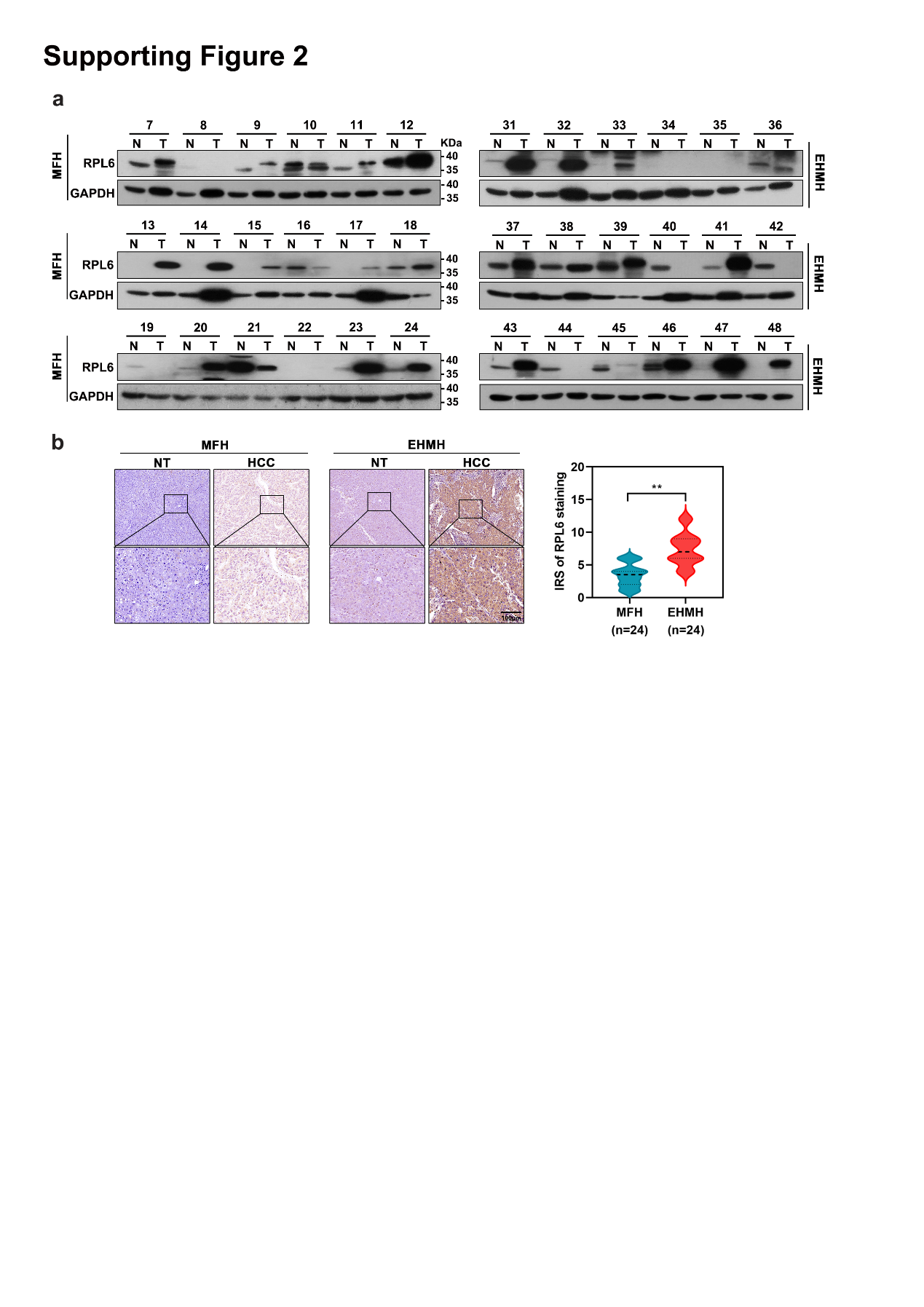


**Supporting Fig. 2 The expression of RPL6 in non-tumor liver tissues and HCC tissues.**

**a**, The protein level of RPL6 in primary HCC tissues with EHMH and MFH were assessed by western blot analysis. Band intensity was quantified using image J. **b**, The representative images showing that RPL6 expression in primary HCC and adjacent non-tumor tissues in EHMH and MFH groups by immunohistochemistry assay, scar bar, 100 μm. Data are shown as mean ± SD. Statistical significance was determined by two-tailed paired Student’s t-test (**b**). ***P* <0.01.


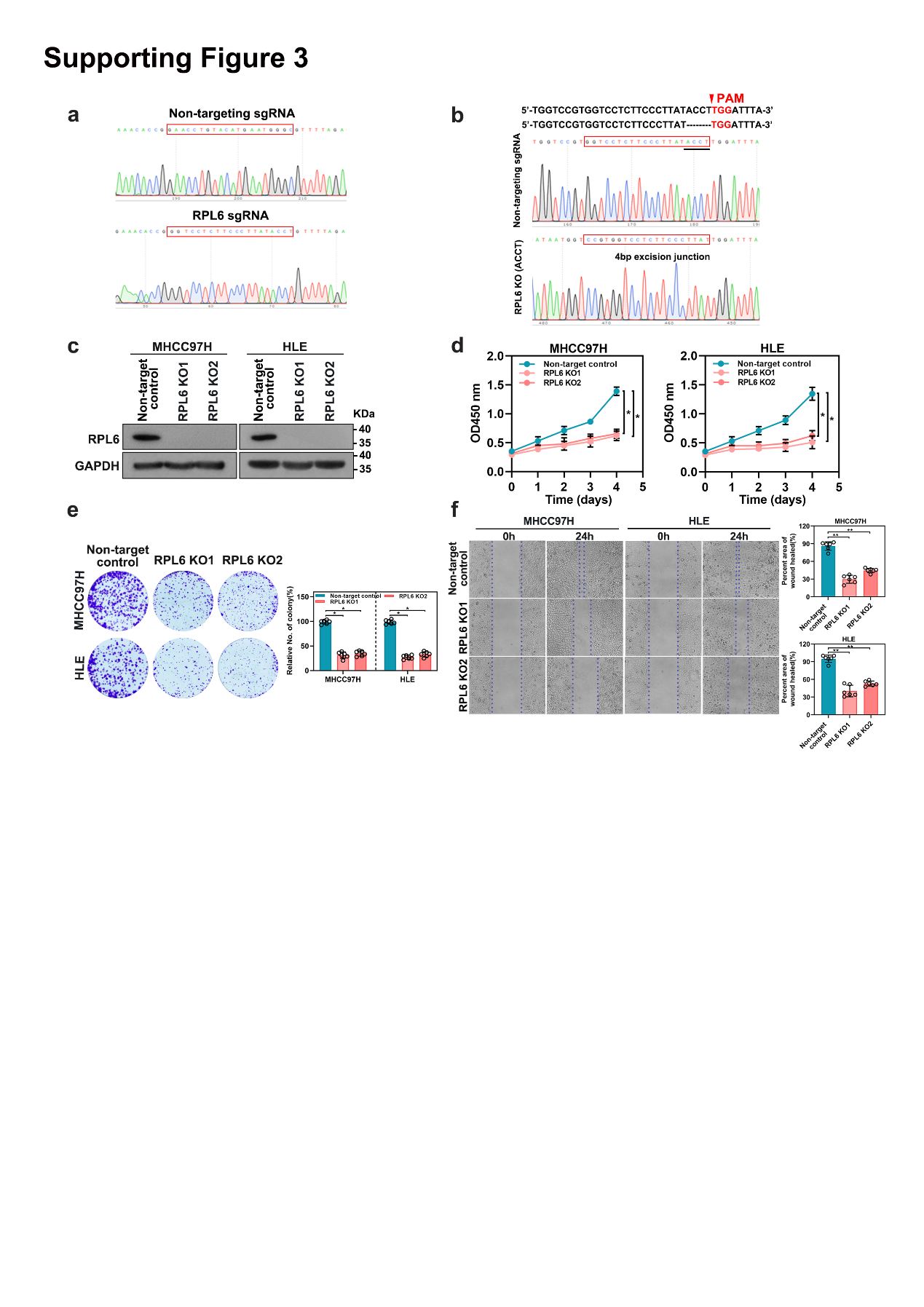


**Supporting Fig. 3 The effect of RPL6 knockout on proliferation and migration in HCC cells.**

**a**, Sequencing results reveal sgRNA sequences inserted at BsmBI cleavage sites, with red boxes indicating the insertion sequences. **b**, Representative sequence comparison shows deletion mutations induced by CRISPR/cas9 system. The PAM sequence (TGG) is highlighted in red, shown above. Sequencing chromatogram of genomic DNA fragments at the RPL6-specific knockout site, with the black solid line marking the mutation site. **c**, Identification of RPL6 knockout in MHCC97H and HLE cells confirmed by western blot assay. **d**,**e**, Assessment of proliferative function in RPL6-knockout MHCC97H and HLE cells using CCK-8 (**d**) and colony formation assays (**e**). **f**, Evaluation of the migratory capacity of RPL6-knockout MHCC97H and HLE cells using scratch wound healing assays. The migration distance was measured in six randomly selected images. Data are shown as mean ± SD of at least three independent experiments (**c**-**f**). Statistical significance was determined by two-tailed unpaired Student’s t-test. **P* <0.05; ***P* <0.01.


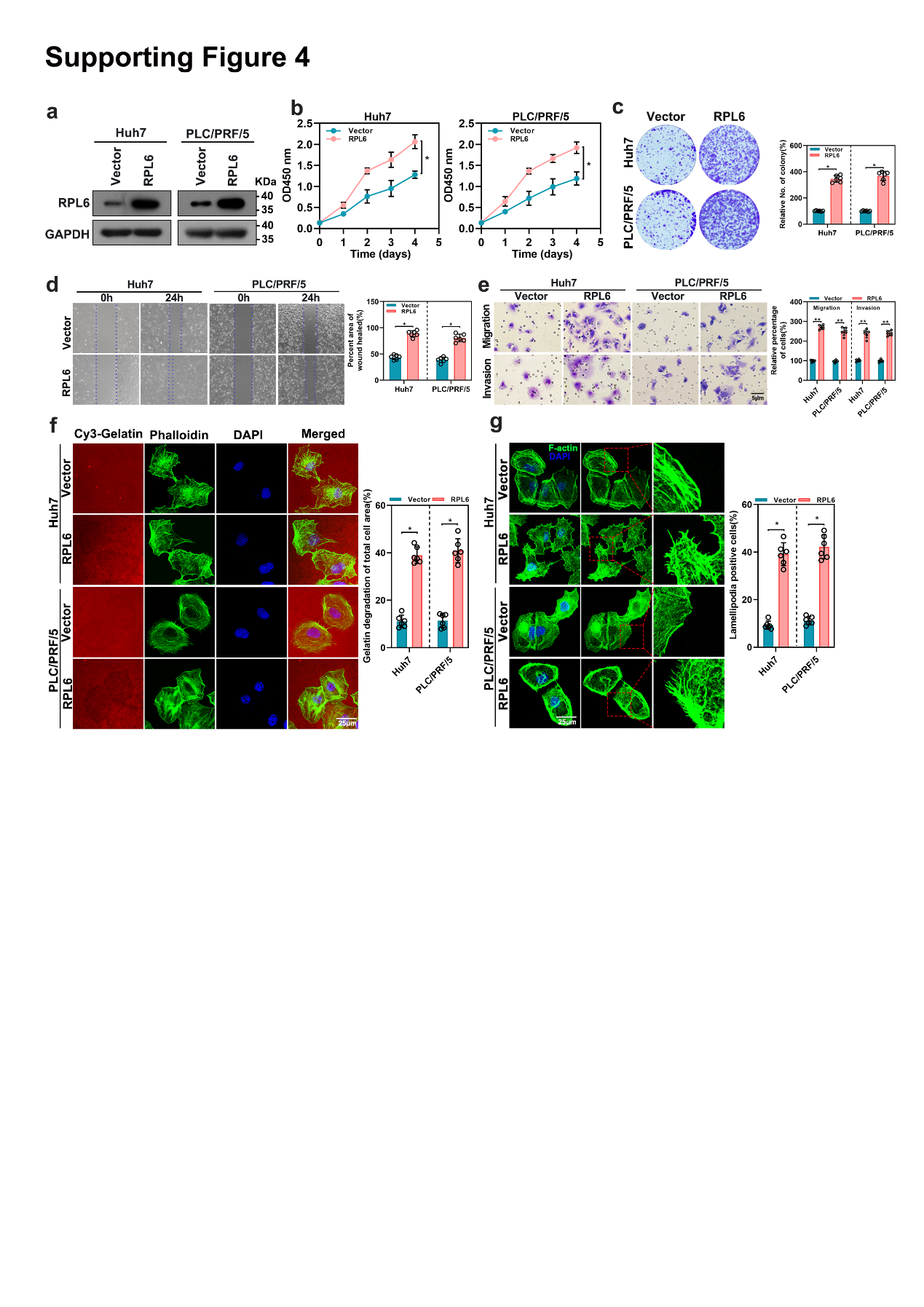


**Supporting Fig. 4 The effect of RPL6 overexpression on HCC cell proliferation and migration in vitro.**

**a**, The overexpression of RPL6 in Huh7 and PLC/PRF/5 cells was confirmed by western blot analysis. **b**,**c**, The effect of RPL6 overexpression on the proliferation of Huh7 and PLC/PRF/5 cells was assessed using CCK-8 assays (**b**) and colony formation assays (**c**). **d**,**e**, The impact of RPL6 overexpression on the migration and invasion of Huh7 and PLC/PRF/5 cells was evaluated using scratch wound healing assays (**d**) and transwell assays (**e**). The distance was measured in six randomly selected images, and the number of cells was counted in 6 images. **f**, The effect of RPL6 overexpression on invadopodia function in Huh7 and PLC/PRF/5 cells was examined by gelatin degradation assay. Representative images were captured with confocal microscopy and were provided. The degraded areas were quantified using Image J software from at least six regions. Scar bar, 25 μm. **g**, The effect of RPL6 overexpression on lamellipodia formation in Huh7 and PLC/PRF/5 cells was examined by F-actin staining. Representative images were shown, and the percentage of cells with lamellipodia formation was counted. Scale bar, 25 μm. Data are shown as mean ± SD of at least three independent experiments. Statistical significance was determined by two-tailed unpaired Student’s t-test. **P* <0.05; ***P* <0.01.


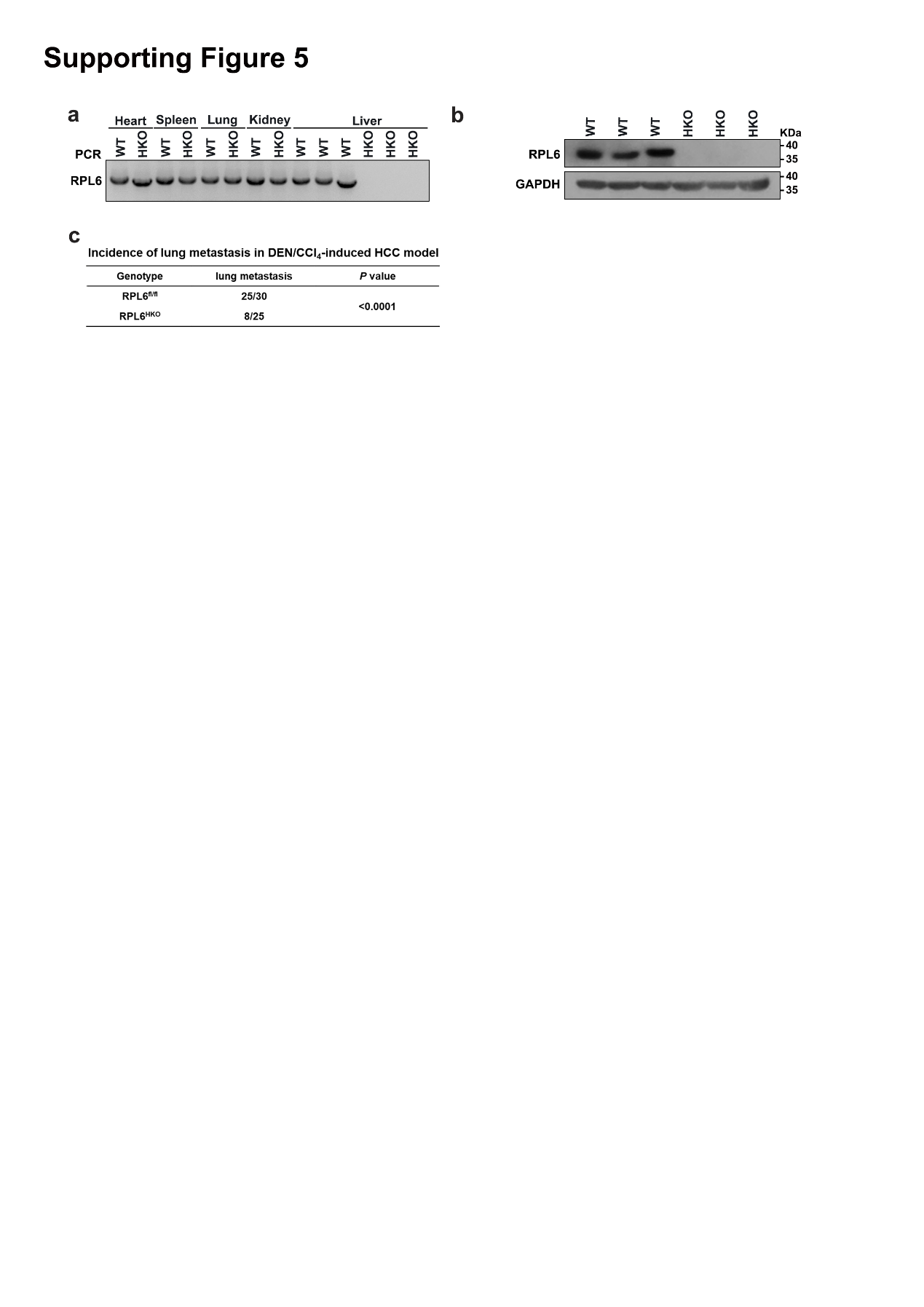


**Supporting Fig. 5 Characterization of RPL6^HKO^ mice.**

**a**, The expression levels of RPL6 in the indicated tissues of RPL6^HKO^ mice were analyzed by qRT-PCR using Cre activity-specific primers. **b**, Specific knockout of hepatic RPL6 was confirmed by western blot analysis, with GAPDH used as a loading control. **c**, Statistical analysis was performed on lung metastasis events in the DEN/CCl_4_-induced hepatocellular carcinoma model for both the RPL6^HKO^ group (n=25) and the RPL6^fl/fl^ group (n=30). Data are shown as mean ± SD of at least three independent experiments (**a,b**). Statistical significance was determined by two-tailed unpaired Student’s t-test.


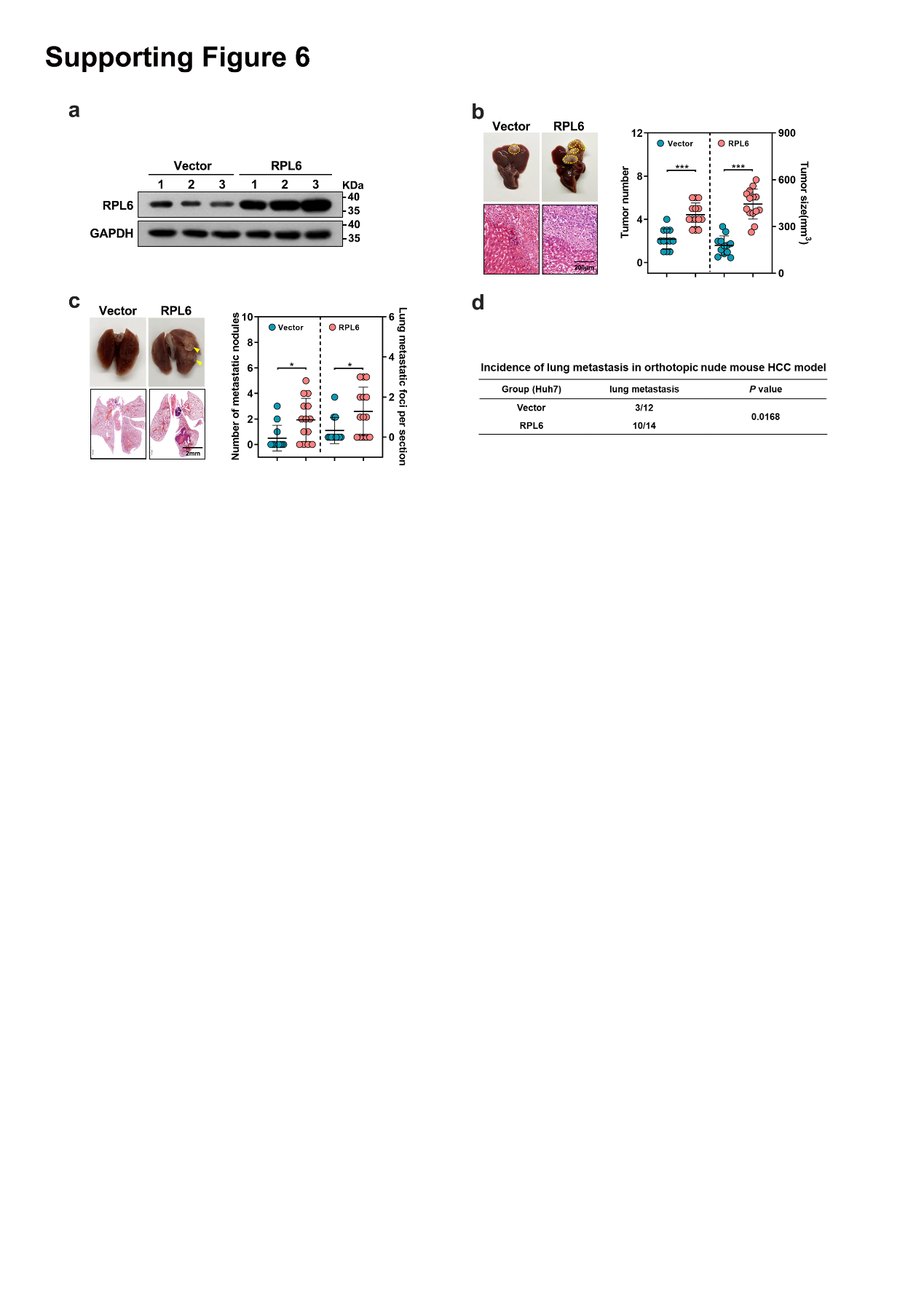


**Supporting Fig. 6 The effect of RPL6 overexpression on HCC metastasis *in vivo*.**

**a-d**, Huh7 cells stably expressing RPL6 and control cells were orthotopically injected into the left lobe liver of nude mice to establish a lung metastasis model. Liver and lung were harvested 8 weeks post-injection for H&E staining. n=12-14 per group. (**a)** Western blot analysis of RPL6 expression in liver tissue. (**b**,**c)** Representative images of tumor-bearing liver and lung tissues were provided. Representative images of H&E staining for liver and lung tissues were provided (**b**,**c**, left). Scale bar, 200 μm, 2 mm. The size and number of liver tumors were counted, and metastatic nodules and foci were calculated (**b**,**c**, right). (**d)** Statistical analysis of the orthotopical injection model for lung metastasis events with or without RPL6 overexpression. Data are shown as mean ± SD. Statistical significance was determined by two-tailed unpaired Student’s t-test. **P* <0.05; ****P* <0.001.


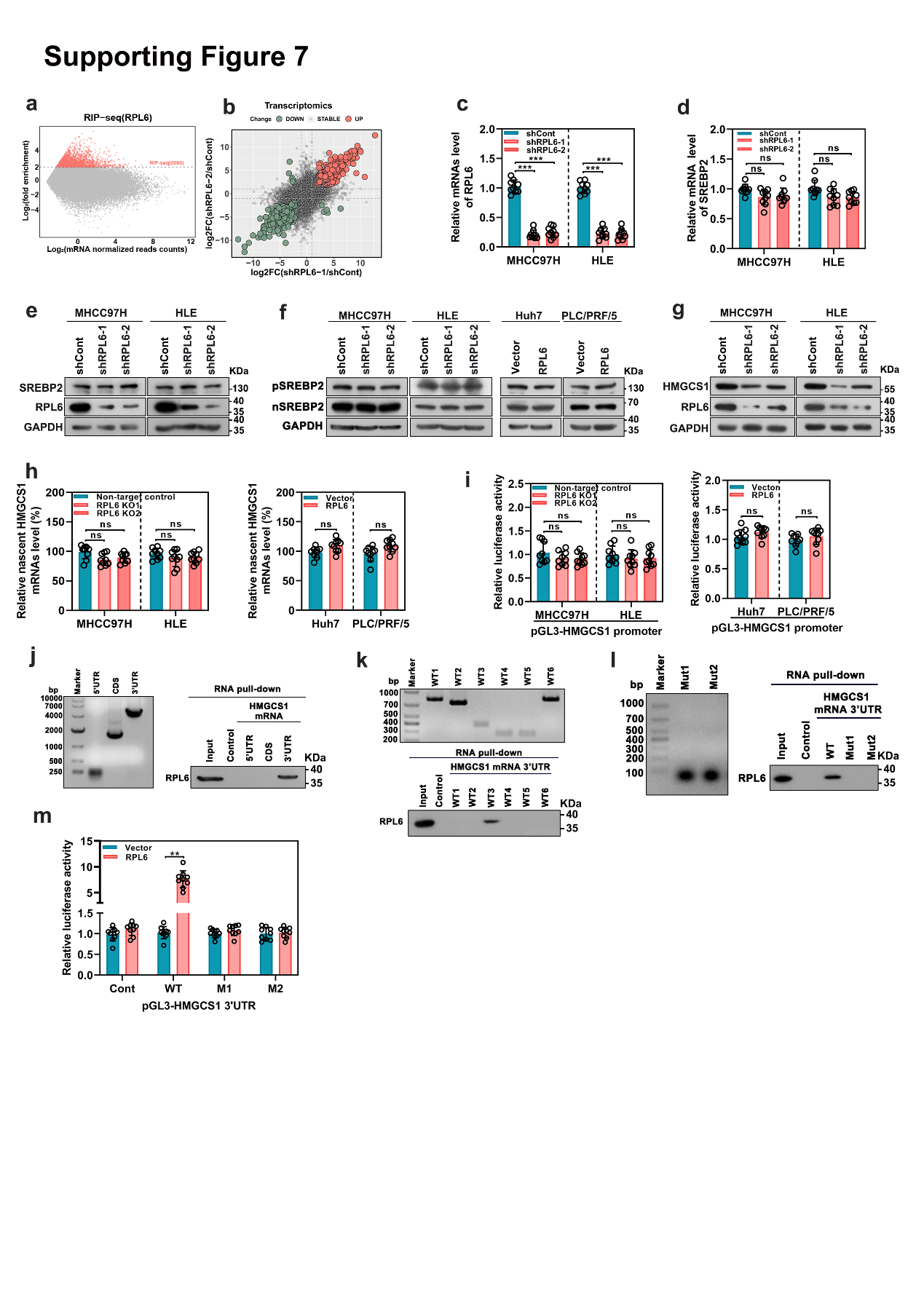


**Supporting Fig. 7 The interaction between RPL6 and HMGCS1 mRNA.**

**a**, A Bland-Altman plot was used to analyze RPL6 RIP-seq in MHCC97H cells. For each transcript, the plot displayed the average signal (measured as log_2_ normalized reads counts) against the RIP-seq log_2_ fold enrichment (RIP versus Input), with significantly enriched targets highlighted in red. **b**, A scatter plot of 9-quadrant associate analysis for RNA-seq data compared negative control and shRPL6 MHCC97H cells. Red dots represent transcripts with log_2_FC＞1 and p value < 0.05, while green dots represent those with log_2_FC＜-1 and p value < 0.05. **c**, The mRNA level of RPL6 in RPL6 silencing HCC cells by qRT-PCR. **d**, The mRNA level of SREBP2 in RPL6-silencing HCC cells by qRT-PCR. **e**, The protein level of SREBP2 in RPL6-silencing HCC cells by western blot analysis. **f**, The protein level of precursor of SREBP2 (pSREBP2) and active form of SREBP2 (nSREBP2) in RPL6-silencing and RPL6-overexpressing cells by western blot analysis. **g**, The protein level of HMGCS1 in RPL6 knockout HCC cells by western blot analysis. **h**, HCC cells with knockout or overexpression of RPL6 were incubated with 0.5 mM EU for 2h. The newly synthesized EU-labeled RNA was purified from the total RNA, and the EU-labelled HMGCS1 RNAs were quantified by qRT-PCR. **i**, The activities of the HMGCS1 promoters in RPL6 knockout or overexpressing HCC cells were determined using a dual-luciferase reporter assay system after transfected with HMGCS1 promoter plasmids for 48h. **j**, PCR confirmed the indicated truncations of HMGCS1 mRNA (left). The binding of RPL6 protein to biotinylated truncations of HMGCS1 mRNA was detected by an RNA pull-down assay (right). **k**,**l**, RNA pull-down assay was performed using the HMGCS1 3’UTR and its indicated truncations. **m**, Dual-luciferase reporter assay determined the luciferase activities of HMGCS1 3’UTR truncations interacting with RPL6. Data are shown as mean ± SD of at least three independent experiments (**c**,**d**,**h**,**i**,**m**). Statistical significance was determined by two-tailed unpaired Student’s t-test. **P <0.01; ***P <0.001. ns, not significant.


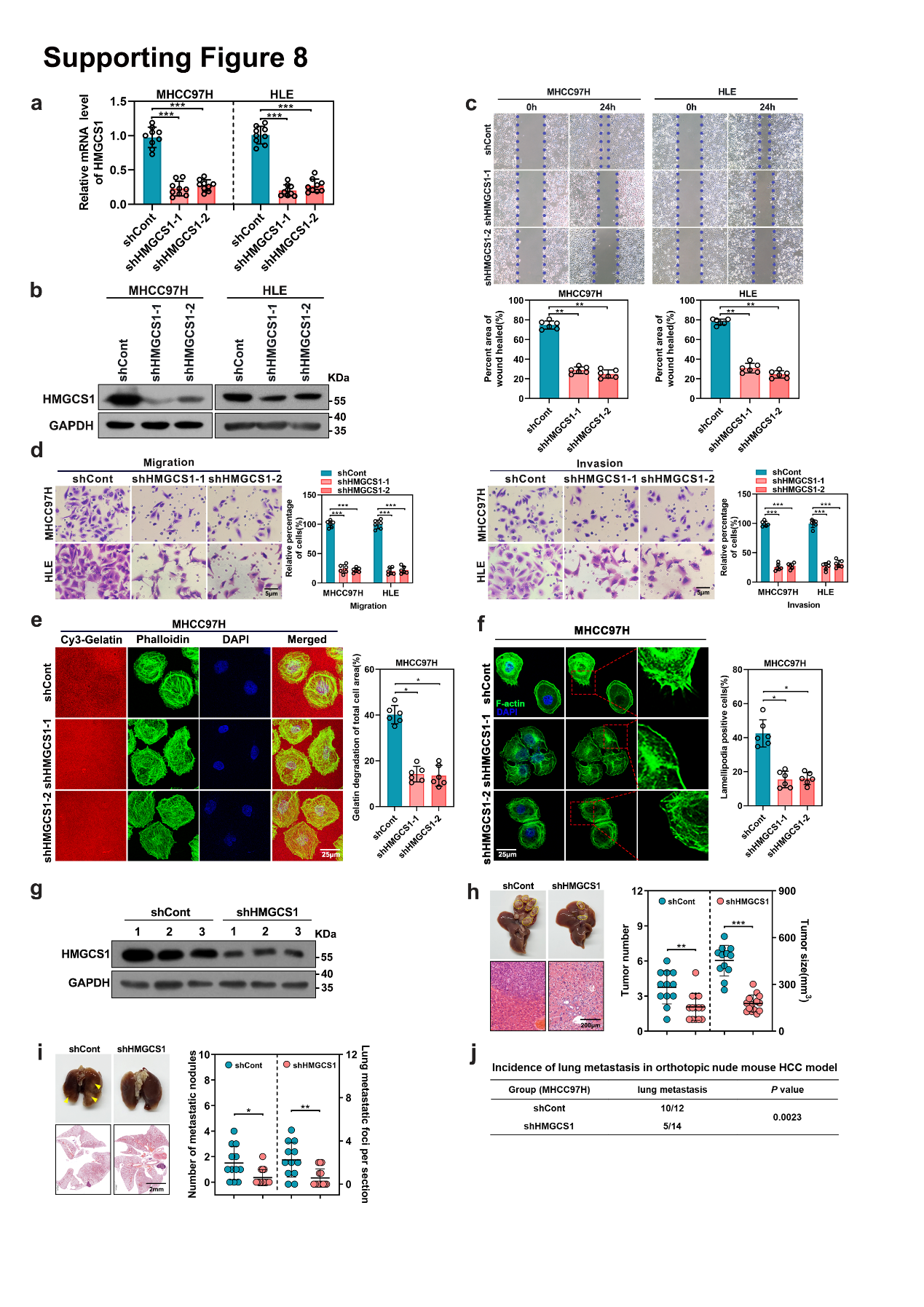


**Supporting Fig. 8 The effect of HMGCS1 knockdown on HCC cell migration and invasion *in vitro* and metastasis *in viv*o.**

**a**,**b**, The efficiency of HMGCS1 knockdown was confirmed by qRT-PCR (**a**) and western blot (**b**). **c**, The effect of HMGCS1 knockdown on the migration of MHCC97H and HLE cells was evaluated by wound healing assays. The distance was measured from 6 images. **d**, The effect of HMGCS1 knockdown on the migration and invasion of MHCC97H and HLE cells was evaluated by transwell assays. The cells were counted from 6 images. **e**, The effect of HMGCS1 knockdown on the invadopodia function of MHCC97H cells was tested by gelatin degradation assay. Representative images were acquired by confocal microscopy and the degraded areas of at least six fields were quantified using image J software. Scar bar, 25 μm. **f**, The effect of HMGCS1 knockdown on lamellipodia formation in MHCC97H cells was determined by F-actin staining. Representative images were shown and the percentage of cells with lamellipodia formation were counted. Scale bar, 25 μm. **g-j**, MHCC97H cells with HMGCS1 stably knockdown were orthotopically injected into the left lobe liver of nude mice to establish a lung metastasis model. Liver and lung were taken at 8 weeks after injection for H&E staining. n=12-14 per group. (**g**) Western blot analysis of HMGCS1 expression in liver tissue. (**h**) Representative macroscopic images and H&E staining of tumor-bearing liver in these mice. The tumor number and tumor size were examined. (**i**) Representative macroscopic images and H&E staining of the lungs with tumor-metastasis from these mice. The metastatic nodules and metastatic foci were calculated. (**j)** Statistical analysis of the orthotopic injection model for lung metastasis events of these mice. Data are shown as mean ± SD of at least three independent experiments (**a**-**f**). Statistical significance was determined by two-tailed unpaired Student’s t-test. **P*< 0.05; ***P* <0.01; ****P* <0.001. ns, not significant.


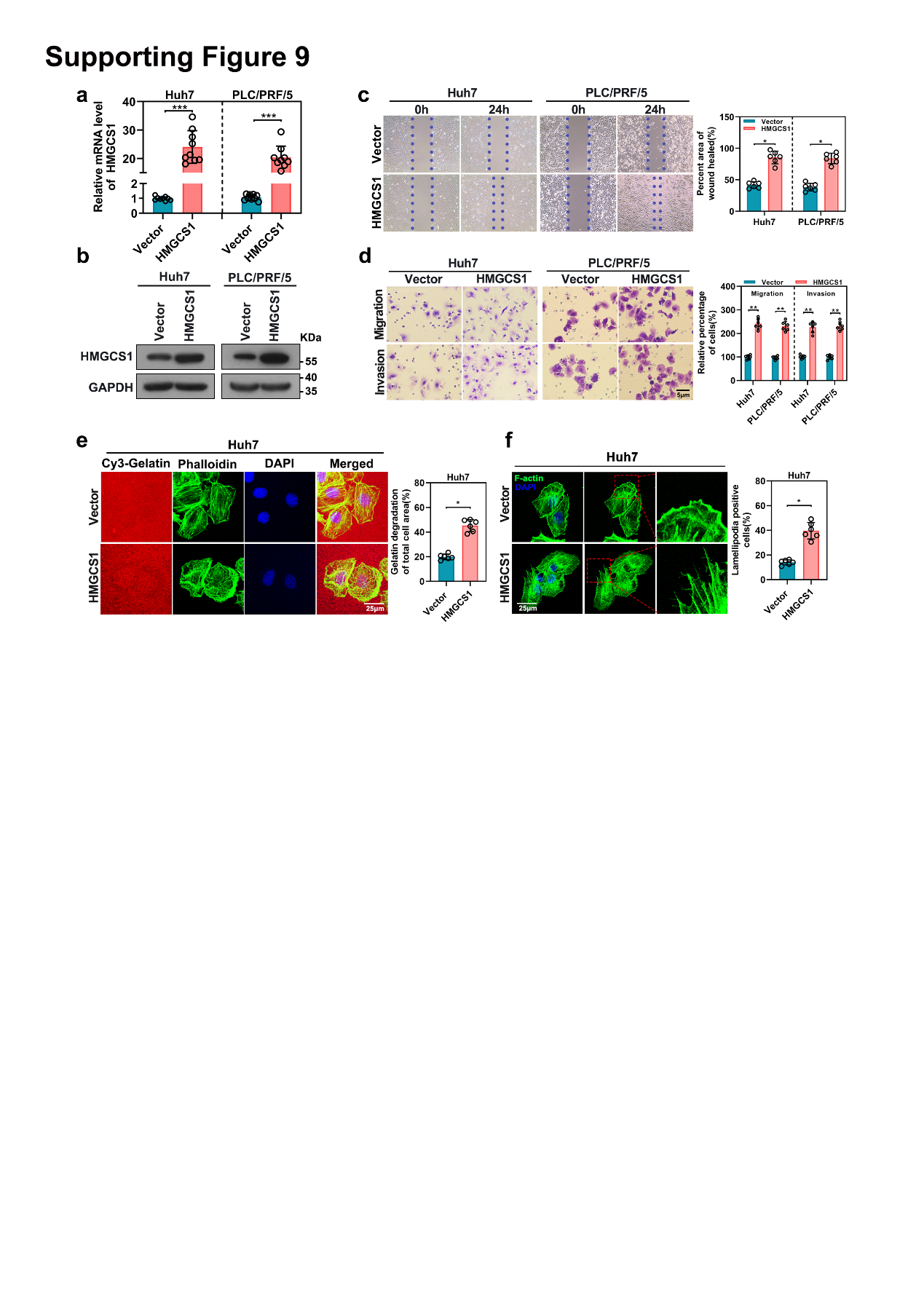


**Supporting Fig. 9 The effect of HMGCS1 overexpression on HCC cell migration and invasion.**

**a**,**b**, The efficiency of HMGCS1 overexpression was confirmed by qRT-PCR (**a**) and western blot (**b**). **c**, The effect of HMGCS1 overexpression on the migration of Huh7 and PLC/PRF/5 cells was evaluated by wound healing assays. The distance was measured from 6 images. **d**, The effect of HMGCS1 overexpression on the migration and invasion of Huh7 and PLC/PRF/5 cells was evaluated by transwell assays. The cells were counted from 6 images. **e**, The effect of HMGCS1 overexpression on the invadopodia function of Huh7 cells was tested by gelatin degradation assay. Representative images were acquired by confocal and the degraded areas of the six fields were quantified using image J software. Scar bar, 25 μm. **f**, The effect of HMGCS1 overexpression on the lamellipodia formation of Huh7 cells was determined by F-actin staining. Representative images were shown and the percentage of cells with lamellipodia formation were counted. Scale bar, 25 μm. Data are shown as mean ± SD of at least three independent experiments. Statistical significance was determined by two-tailed unpaired Student’s t-test. **P* <0.05; ***P* <0.01; ****P* <0.001.


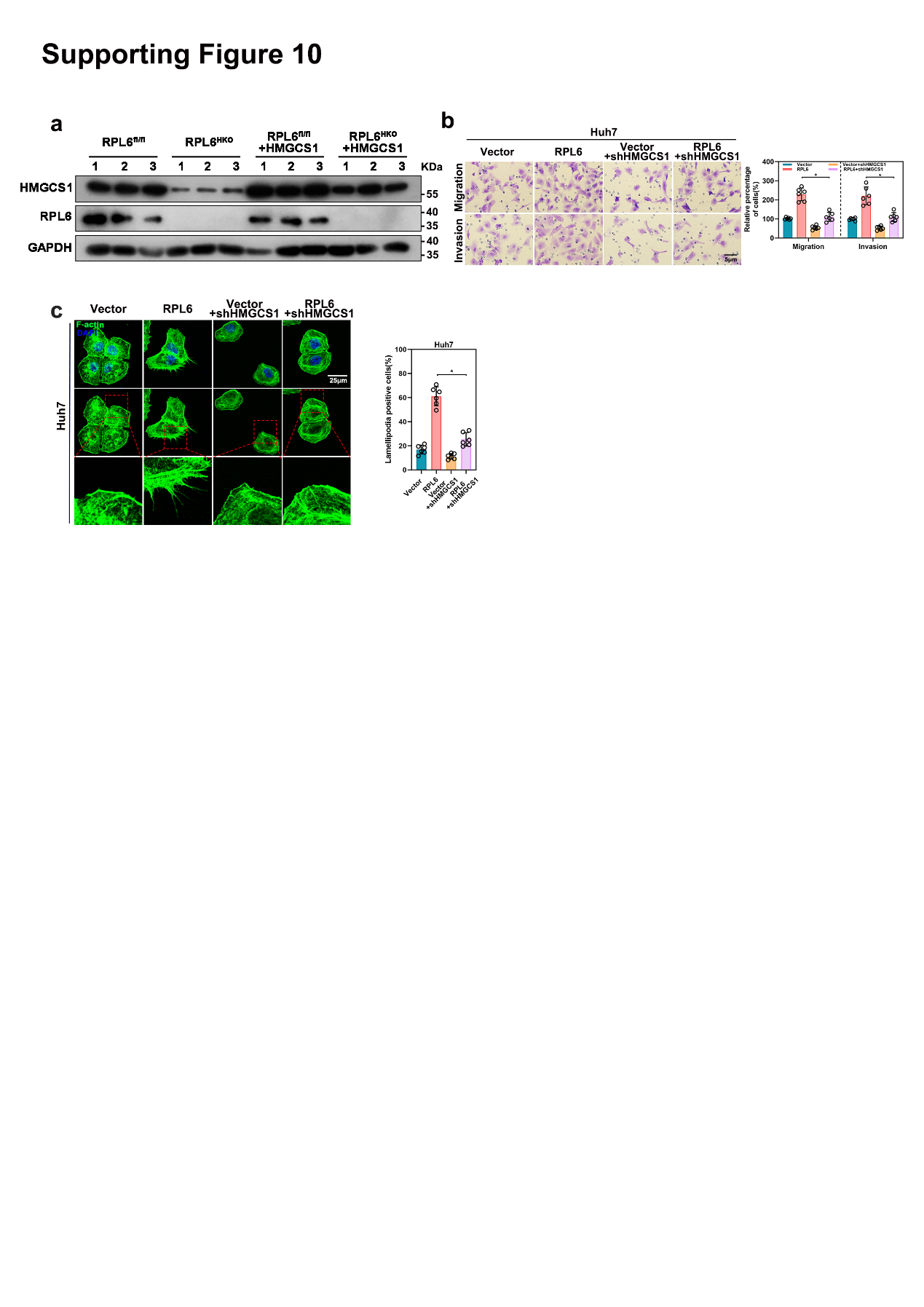


**Supporting Fig. 10 HMGCS1 involved in RPL6-mediated HCC metastasis.**

**a**, Western blot analysis of HMGCS1 and RPL6 expression in liver tissue of RPL6^HKO^ mice. **b**, The effect of HMGCS1 knockdown on the migration and invasion of RPL6 overexpression cells was assessed by transwell assays. The cells were counted from 6 images. **c**, The effect of HMGCS1 knockdown on the lamellipodia formation of RPL6 overexpression was tested by F-actin staining assay. Representative images were acquired by confocal and the percentage of cells with lamellipodia formation were counted from at least six fields. Scar bar, 25 μm. Statistical significance was determined by one-way analysis of variance (ANOVA) (**b**,**c**). **P* <0.05.


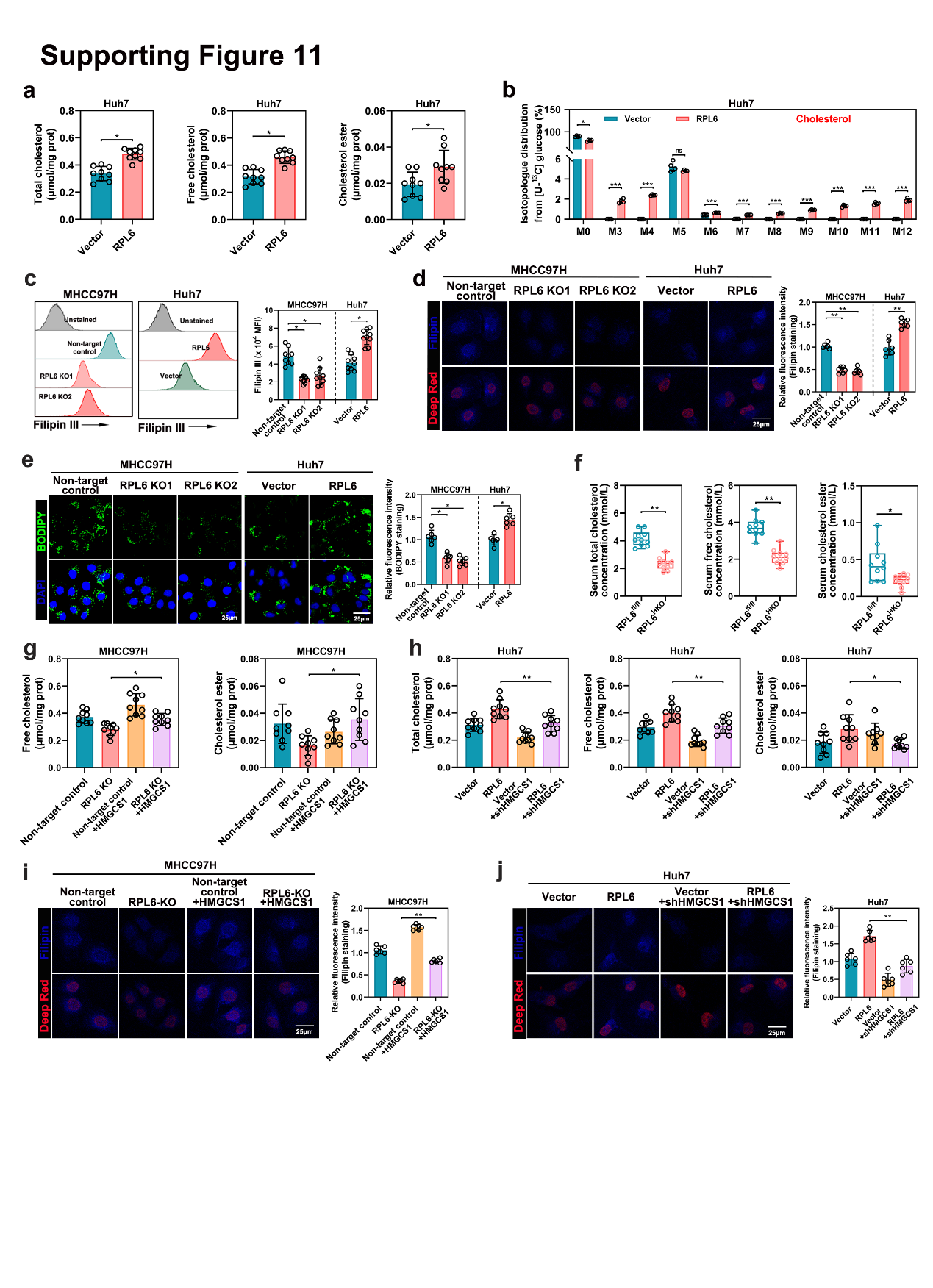


**Supporting Fig. 11 RPL6 upregulated HMGCS1 to elevate cholesterol level in HCC.**

**a**, Contents of total, free and esterified cholesterol in RPL6-overexpressing Huh7 cells. **b**, Measurement of cholesterol synthesis by stable isotope labelling of RPL6 overexpression versus control Huh7 cells with ^13^C-glucose (n=4). **c**, Representative FCM profiles and the MFI of Filipin III staining in RPL6-knockout MHCC97H and RPL6-overexpressing Huh7 cells. **d**, Filipin III staining used to visualize cellular free cholesterol distributions in RPL6-knockout MHCC97H and RPL6-overexpressing Huh7 cells. Scale bar, 25 μm. The fluorescence intensity of Filipin III staining was quantified by using Image J software from at least six fields. **e**, Representative BODIPY staining images (left) and quantitative analysis (right) of RPL6-knockout MHCC97H and RPL6-overexpressing Huh7 cells. Scale bar, 25 μm. **f**, Levels of total, free and esterified cholesterol in serum of RPL6^HKO^ and RPL6^fl/fl^ mice (n=10). **g**, The free and esterified cholesterol contents in HMGCS1 overexpression of RPL6-knockout cells. **h**, Levels of total, free and esterified cholesterol in HMGCS1 knockdown of RPL6-overexpressing cells. **i,j**, Filipin III staining was utilized to determine cellular cholesterol distributions in HMGCS1 overexpression of RPL6-knockout cells (**i**), and HMGCS1 knockdown of RPL6-overexpressing cells (**j**). Scale bar, 25 μm. Data are shown as mean ± SD of at least three independent experiments. Statistical significance was determined by two-tailed unpaired Student’s t-test (**a-f**) or one-way analysis of variance (ANOVA) (**g**-**j**). *P <0.05; **P <0.01, ****P* <0.001.


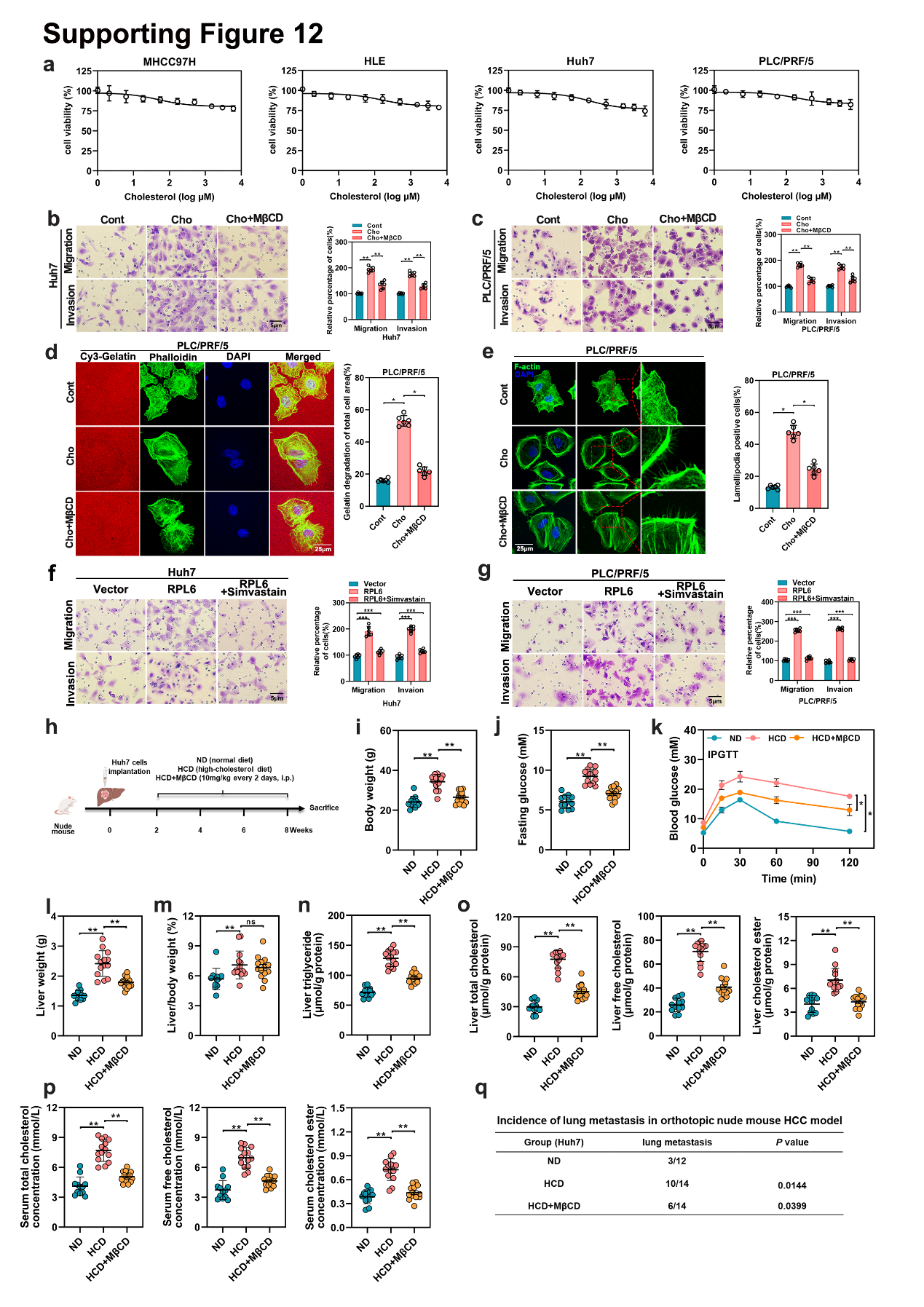


**Supporting Fig. 12 The effect of cholesterol on HCC cell migration and invasion *in vitro* and metastasis *in vivo*.**

**a**, MHCC97H, HLE, Huh7 and PLC/PRF/5 cells were treated with various concentrations of cholesterol for 72 h. The effects of cholesterol on cell viability were determined by the MTT assay. **b**,**c**, The effect of cholesterol (10 μM) and the addition of methyl-b-cyclodextrin (MβCD) on migration and invasion function in Huh7 (**b**) and PLC/PRF/5 (**c**) cells was tested by transwell assays. The cells were counted from 6 images. **d**, The effect of cholesterol (10 μM) and the addition of methyl-b-cyclodextrin (MβCD) on invadopodia function in Huh7 and PLC/PRF/5 cells was tested by gelatin degradation assay. Images were obtained by confocal and the degraded areas of at least six fields were quantified using image J software. Scale bar, 25 μm. **e**, The effect of cholesterol (10 μM) and the addition of methyl-b-cyclodextrin (MβCD) on lamellipodia formation in PLC/PRF/5 cells was evaluated through F-actin staining. Representative images were shown and the percentage of cells with lamellipodia formation were counted from at least six regions. Scale bar, 25 μm. **f**,**g**, The effect of RPL6 and simvastatin (2 μM) treatment on migration and invasion function in Huh7 (**f**) and PLC/PRF/5 (**g**) cells was tested by transwell assays. The cells were counted from 6 images. **h**, Illustration of the HCD-fed or MβCD therapeutic study. Two weeks after Huh7 cells implantation, then mice were treated with ND or HCD, or MβCD treatment (10 mg/kg of mouse, i.p.) every 2 days for 6 weeks. n=12-14 per group. **i**-**p**, Body wight (**i**), fasting glucose (**j**), glucose tolerance test (**k**), liver weight (**l**), liver-body weight ratios (**m**), liver triglyceride (**n**), total, free and esterified cholesterol levels of liver tissues (**o**) and serum (**p**) in mice fed with ND, HCD and HCD with MβCD treatment. **q**, Statistical analysis of orthotopical injection model for lung metastasis events of these mice. Data are shown as mean ± SD of at least three independent experiments (**a**-**g**). Statistical significance was determined by two-tailed unpaired Student’s t-test (**b**-**g**,**i**,**j,l**-**q**) or one-way analysis of variance (ANOVA) (**k**). **P* <0.05; ***P* <0.01. ns, not significant.


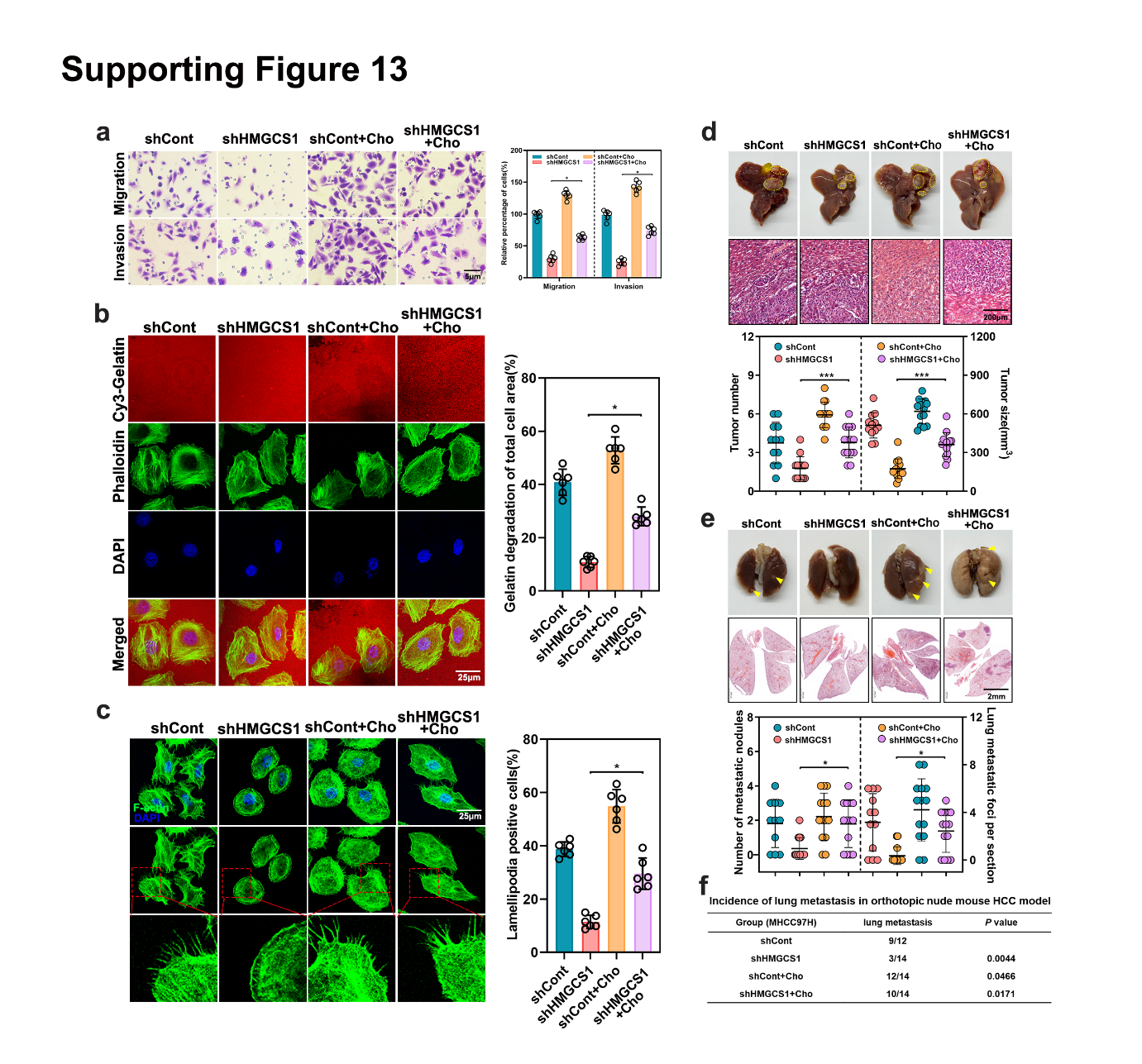


**Supporting Fig. 13 Intracellular cholesterol involves in maintenance of HCC migration and invasion *in vitro* and metastasis *in vivo*.**

**a**, The effect of cholesterol (10 μM) on migration and invasion in HMGCS1 knockdown cells was tested by transwell assays. The cells were counted from 6 images. **b**, The effect of cholesterol (10 μM) on invadopodia function in HMGCS1-knockdown cells was determined by gelatin degradation assay. Representative images were captured by confocal and the degraded areas of at least six fields were quantified using image J software. Scar bar, 25 μm. **c**, The effect of cholesterol (10 μM) on lamellipodia function in HMGCS1-knockdown cells was determined by F-actin staining assay. Representative images were shown and the percentage of cells with lamellipodia formation was counted. Scar bar, 25 μm. **d**-**f**, MHCC97H cells with stable knockdown of HMGCS1 or control cells were orthotopically injected into the left lobe liver of nude mice to establish a lung metastasis model, following the treatments of ND or HCD. Liver and lung tissues collected at 8 weeks post-injection were used for detection. n=12-14 per group. (**d**) Representative macroscopic images and H&E staining of tumor-bearing liver in these mice. The tumor number and tumor size were examined. Scar bar, 200 μm. (**e**) Representative macroscopic images and H&E staining of tumor-metastasis lung from these mice. The number of metastatic nodules and metastatic foci were examined. Scar bar, 2 mm. (**f**) Statistical analysis for lung metastasis events of these mice. Data are shown as mean ± SD of at least three independent experiments (**a**-**c**). Statistical significance was determined by one-way analysis of variance (ANOVA). **P* <0.05; ****P* <0.001.


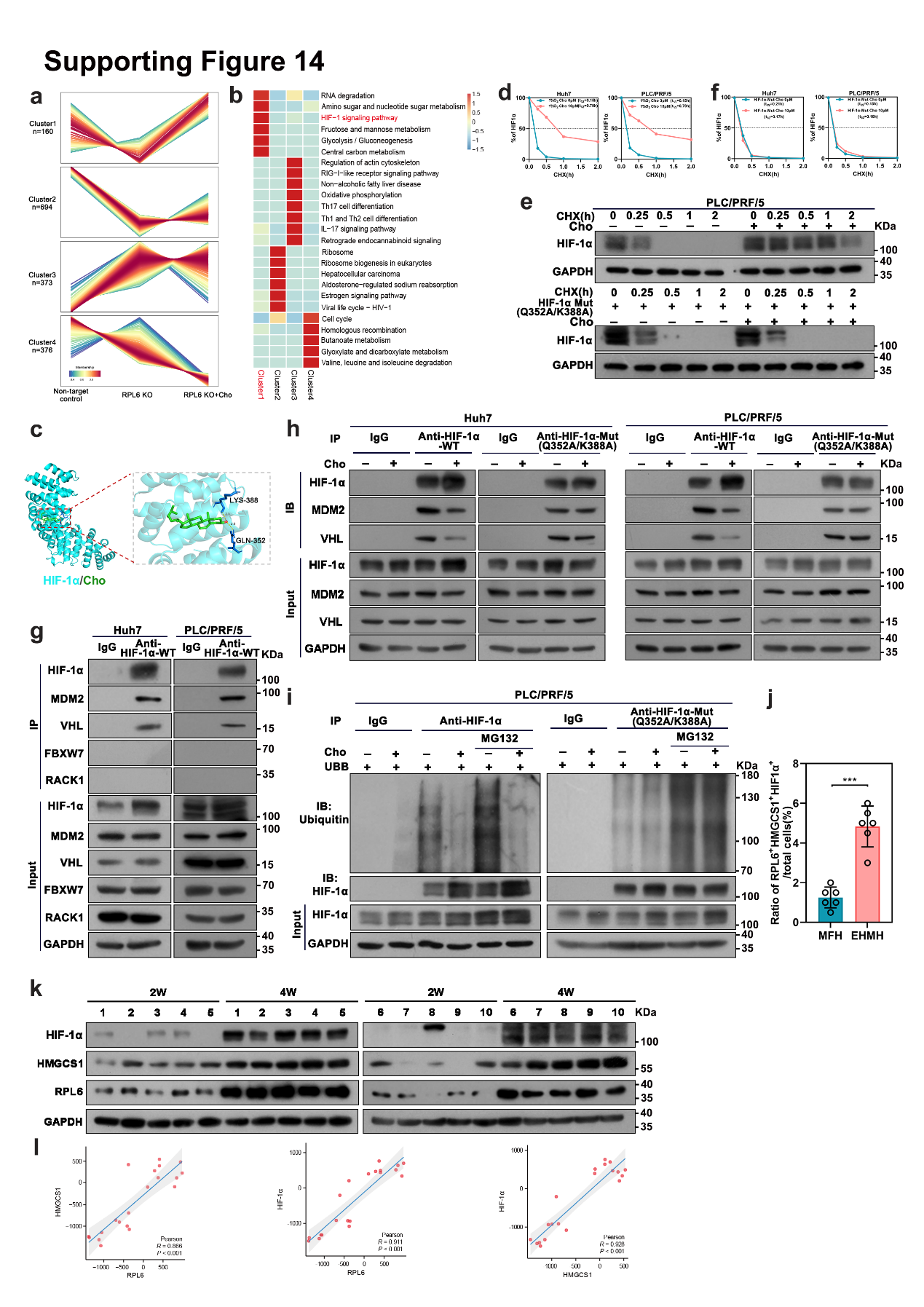


**Supporting Fig. 14 cholesterol stabilizes HIF-1α protein through inhibiting its ubiquitin-dependent degradation**

**a**, Clustering of proteins according to differentially modulated expression patterns. **b**, KEGG analysis of differentially regulated proteins across all 4 clusters as in (**a**). **c**, The interaction structure between cholesterol (green) and HIF-1α (blue) was analyzed through molecular docking. **d**, HIF-1α was examined by western blot and the protein levels were quantified using Image J and normalized to GAPDH intensities in Figure 7f. **e,f**, Cells treated with cholesterol (10 µM) were exposed to hypoxia for 6 h (**e**, upper), or transfected with HIF-1α-MUT plasmid for 24h (**e**, lower) then incubated with 10 μg/mL cycloheximide at the indicated times. HIF-1α was examined by western blot and the protein levels were quantified using Image J and normalized to GAPDH intensities (**f**). **g**, Cells transfected with HIF-1α-WT plasmid for 24h, immunoprecipitated using anti-HIF-1α antibody and immunoblotted by anti-MDM2/VHL/FBXW7/RACK1 antibody. **h**, After transfected with HIF-1α-WT or HIF-1α-MUT plasmids and treated with cholesterol (10μM), cells were immunoprecipitated using anti-HIF-1α antibody and immunoblotted by anti-MDM2/VHL antibody. **i**, PLC/PRF/5 cells transfected with HA-Ub plasmid and treated with cholesterol (10 μM) under hypoxia for 6 h (left) or transfected with HIF-1α-MUT plasmid (right), were then exposed to MG132 (10 μM). Whole-cell extracts were immunoprecipitated with anti-HIF-1α antibody, and ubiquitinated-HIF-1α was detected using an anti-ubiquitin antibody. **j**, Quantification results for RPL6+HMGCS1+HIF-1α+ cells out of the total cells in MFH and EHMH tissues were calculated. **k,l**, The expression of RPL6, HMGCS1, and HIF-1α in the pulmonary metastasis HCC mouse were examined by using immunoblotting (**k**). Correlation analysis of expression level performed using the Pearson test (**l**). Data are shown as mean ± SD of at least three independent experiments. Statistical significance was determined by two-tailed unpaired Student’s t-test (**d**,**f**,**j**). ****P* <0.001.


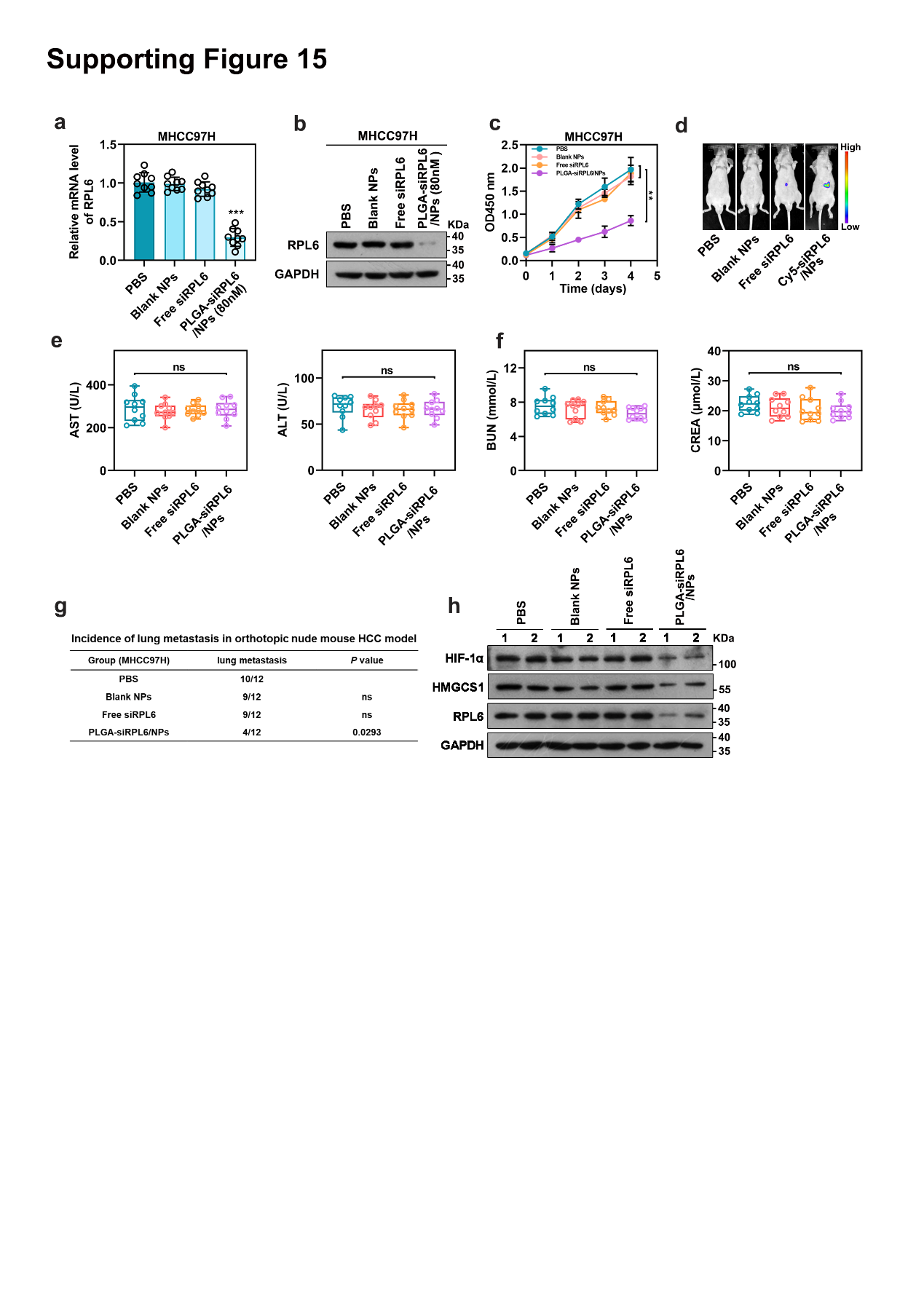


**Supporting Fig. 15 Characterization and therapeutic efficacy of PLGA-siRPL6/NPs in HCC model.**

**a**,**b**, The levels of RPL6 treated with PBS, blank NPs, free siRPL6 or PLGA-siRPL6/NPs were determined by qRT-PCR (**a**) and western blot (**b**). **c**, The effect of PLGA-siRPL6/NPs treatment on the proliferation of MHCC97H cells was tested by CCK-8 assays. **d**, Biodistribution of PLGA-Cy5-siRNA/NPs complex in vivo was studied. **e**, Blood levels of AST and ALT were detected in mice injected with indicated NPs. **f**, Blood levels of BUN and CREA were detected in mice injected with indicated NPs. **g**, Statistical analysis was conducted to evaluate lung metastasis events in mice treated with PLGA-siRPL6/NPs. **h**, Western blot analysis was used to measure the expression levels of HIF-1α, HMGCS1 and RPL6 in the tumor tissues of mice treated with PLGA-siRPL6/NPs. Data are shown as mean ± SD of at least three independent experiments (**a**-**c**). Statistical significance was determined by two-tailed unpaired Student’s t-test (**a**) or one-way analysis of variance (ANOVA) (**c**,**e**-**g**). ***P* <0.01; ****P* <0.001. ns, not significant.

**Supporting Table 1. Correlation between RPL6 expression and clinicopathological characteristics in 48 HCC patients**

| **Features** | **No. of Specimens** | **expression of RPL6** | |  |
| --- | --- | --- | --- | --- |
|  |  | **High(n=37)** | **Low(n=11)** | ***P* Value** |
| Gender |  |  |  |  |
| Males | 39 | 30 | 9 | 0.7003 |
| Females | 9 | 7 | 2 |  |
| Age(years) |  |  |  |  |
| ≤50 | 19 | 17 | 2 | 0.1929 |
| ＞50 | 29 | 20 | 9 |  |
| AFP (ug/L) |  |  |  |  |
| ≤20 | 26 | 21 | 5 | 0.5089 |
| ＞20 | 22 | 16 | 6 |  |
| Liver cirrhosis |  |  |  |  |
| Yes | 34 | 26 | 8 | 0.8256 |
| No | 14 | 11 | 3 |  |
| Tumor size |  |  |  |  |
| ≤3cm | 7 | 5 | 2 | 0.9193 |
| >3cm | 41 | 32 | 9 |  |
| Multiple tumor |  |  |  |  |
| Yes | 4 | 2 | 2 | 0.2213 |
| No | 44 | 35 | 9 |  |
| Tumor encapsulation |  |  |  |  |
| Absent | 30 | 23 | 7 | 0.7902 |
| Present | 18 | 14 | 4 |  |
| PVTT |  |  |  |  |
| Yes | 8 | 6 | 2 | 0.784 |
| No | 40 | 32 | 9 |  |
| Vascular invasion | |  |  |  |
| Yes | 28 | 25 | 3 | **0.0422*** |
| No | 20 | 12 | 8 |  |
| Lung metastasis |  |  |  |  |
| Yes | 17 | 15 | 2 | **0.0308*** |
| No | 31 | 22 | 9 |  |
| TNM stage |  |  |  |  |
| I and II | 29 | 26 | 3 | **0.0105*** |
| III and Ⅳ | 19 | 11 | 8 |  |

*Pearson chi-square test. The bold values mean the clinical variable with statistically significance.

**Supporting Table 2. Mean numbers of blood cells, and parameters for liver and kidney toxicity induced by treatment with MβCD in nude mice.**

|  | MβCD dose | | |
| --- | --- | --- | --- |
|  | 0 mg/kg/ 2 days | 5 mg/kg/2 days | 10 mg/kg/2 days |
| Body weight (g) | 21.52±1.08 | 26.09±0.92 ^ns^ | 23.01±0.85 ^ns^ |
| WBC (×10^3^/μL) | 6.962±1.229 | 5.75±2.004 ^ns^ | 6.046±1.005 ^ns^ |
| RBC (×10^6^/μL) | 9.016±1.989 | 9.706±1.056 ^ns^ | 10.044±0.792 ^ns^ |
| Hemoglobin (g/dL) | 14.96±2.05 | 15.82±2.04 ^ns^ | 15.08±2.33 ^ns^ |
| Platelets (×10^3^/μL) | 714.48±175.23 | 739.46±129.14 ^ns^ | 809.96±147.26 ^ns^ |
| Total protein (g/dL) | 5.29±2.05 | 5.73±1.49 ^ns^ | 6.08±1.19 ^ns^ |
| Albumin (g/dL) | 2.94±0.21 | 2.81±0.31 ^ns^ | 2.72±0.48 ^ns^ |
| ALT (U/L) | 65.3±18.6 | 62.5±20.9 ^ns^ | 58.7±23.5 ^ns^ |
| AST (U/L) | 86±15.6 | 84.7±20.1 ^ns^ | 76.1±11.9 ^ns^ |
| ALP (U/L) | 76.2±11.3 | 79.2±7.0 ^ns^ | 80.4±7.1 ^ns^ |
| GGT (U/L) | 7.3±1.6 | 6.49±1.98 ^ns^ | 7.21±1.29 ^ns^ |
| TBIL (mg/dL) | 1.99±1.81 | 2.37±1.04 ^ns^ | 2.18±1.58 ^ns^ |
| CREA (μmol/L) | 22.12±0.224 | 20.71±2.806 ^ns^ | 21.16±0.301 ^ns^ |
| BUN (mmol/L) | 10.2±2.6 | 9.81±2.49 ^ns^ | 11.2±1.58 ^ns^ |

All values are expressed as the mean ± Standard deviation.

Abbreviations: WBC, white blood cell; RBC, red blood cell; ALT, alanine transaminase; AST, aspartate transaminase; ALP, alkaline phosphatase; GGT, γ-glutamyl transpeptidase; TBIL, total bilirubin; CREA, creatinine; BUN, blood urea nitrogen. ns, no significance (*P* > 0.05) compared to the 0 g/kg/week MβCD group.

**Supporting Table 3. Mean numbers of blood cells, and parameters for liver and kidney toxicity induced by treatment with PLGA-siRPL6/NPs in NSG mice.**

|  | PLGA-siRPL6/NPs dose | | |
| --- | --- | --- | --- |
|  | 0 mg/kg/2 days | 1.25 mg/kg/2 days | 2.5 mg/kg/2 days |
| Body weight (g) | 24.12±1.01 | 24.07±0.97 ^ns^ | 23.48±1.66 ^ns^ |
| WBC (×10^3^/μL) | 7.19±1.34 | 6.69±1.75 ^ns^ | 7.24±0.178 ^ns^ |
| RBC (×10^6^/μL) | 10.271±1.525 | 9.847±1.004 ^ns^ | 9.791±1.068 ^ns^ |
| Hemoglobin (g/dL) | 15.93±1.05 | 15.76±1.15 ^ns^ | 16.08±0.894 ^ns^ |
| Platelets (×10^3^/μL) | 747.25±132.24 | 756.81±130.822 ^ns^ | 805.6±163.205 ^ns^ |
| Total protein (g/dL) | 6.29±2.59 | 5.82±3.07 ^ns^ | 6.12±2.74 ^ns^ |
| Albumin (g/dL) | 2.45±0.14 | 2.52±0.28 ^ns^ | 2.49±0.37 ^ns^ |
| ALT (U/L) | 48.5±11.6 | 39.4±9.4 ^ns^ | 35.8±8.2 ^ns^ |
| AST (U/L) | 51.8±14.4 | 42.6±9.8 ^ns^ | 39.2±7.7 ^ns^ |
| ALP (U/L) | 70.2±18.3 | 77.2±9.14 ^ns^ | 83.4±2.84 ^ns^ |
| GGT (U/L) | 6.8±1.2 | 6.24±1.84 ^ns^ | 7.21±1.59 ^ns^ |
| TBIL (mg/dL) | 1.72±0.81 | 1.94±1.77 ^ns^ | 2.33±1.55 ^ns^ |
| CREA (μmol/L) | 20.82±0.183 | 20.36±0.914 ^ns^ | 19.14±2.09 ^ns^ |
| BUN (mmol/L) | 19.96±1.05 | 17.37±2.48 ^ns^ | 18.84±1.67 ^ns^ |

All values are expressed as the mean ± Standard deviation.

Abbreviations: WBC, white blood cell; RBC, red blood cell; ALT, alanine transaminase; AST, aspartate transaminase; ALP, alkaline phosphatase; GGT, γ-glutamyl transpeptidase; TBIL, total bilirubin; CREA, creatinine; BUN, blood urea nitrogen. ns, no significance (*P* > 0.05) compared to the 0 mg/kg/2 days PLGA-siRPL6/NPs group.

**Supporting Table 4. The cDNA target sequences of shRNAs, primer sequences, and CRISPR gRNA sequences used in this study**

| **Primers** | **Sequences (5’-3’)** |
| --- | --- |
| **Primers sequences used for real-time PCR** | |
| RPL6 F | CCTTGTCAGAGGAATTGGCAGG |
| RPL6 R | GTAACAGTTGCGAGAACCTTCTC |
| β-actin F | CTCTTCCAGCCTTCCTTCCT |
| β-actin R | AGCACTGTGTTGGCGTACAG |
| HMGCS1 F | TCTCTATCCTTCACACAGCTCTT |
| HMGCS1 R | AGCCGCCCAATGCAATCATA |
| HIF1α-F | TATGAGCCAGAAGAACTTTTAGGC |
| HIF1α-R | CACCTCTTTTGGCAAGCATCCTG |
| VEGFA-F | AGGGAAAGGGGCAAAAACGA |
| VEGFA-R | CCTCGGCTTGTCACATCTGC |
| FLT1-F | TCATTCCGAAGCAAGGTGTGA |
| FLT1-R | AGAGTCAGCCACAACCAAGG |
| ANG-F | ATTCTTCCTCCTGGGAGCCTGT |
| ANG-R | GCCCTGTGGTTTGGCATCAT |
| TWIST1-F | GCCGGAGACCTAGATGTCATT |
| TWIST1-R | TTTTAAAAGTGCGCCCCACG |
| TGFA-F | TGCCATTCTGGGTACGTTGG |
| TGFA-R | TGGGCTCTTCAGACCACTGT |
| TGFβ1-F | GGAAATTGAGGGCTTTCGCC |
| TGFβ1-R | CGGTAGTGAACCCGTTGAT |
| ANP-F | CAGACCTGATGGATTTCAAGAATTT |
| ANP -R | CGCCCTCAGCTTGCTTTTTAG |
| ANGPT1-F | TCTTTGCACTAAAGAAGGTGTTTT |
| ANGPT1-R | TTACAGTCCAACCTCCCCCA |
| ANGPTL4-F | CCTCTCCGTACCCTTCTCCA |
| ANGPTL4-R | AAACCACCAGCCTCCAGAGA |
| ZEB1-F | GGCATACACCTACTCAACTACGG |
| ZEB1-R | TGGGCGGTGTAGAATCAGAGTC |
| Vimentin-F | AGGCAAAGCAGGAGTCCACTGA |
| Vimentin -R | ATCTGGCGTTCCAGGGACTCAT |
| SREBP2 F | TGTGTATGTCCTGTGCCTTTTC |
| SREBP2 R | TGGGACACAGTGACTGATTGAT |
| HMGCR-F | TGATTGACCTTTCCAGAGCAAG |
| HMGCR-R | CTAAAATTGCCATTCCACGAGC |
| MVK-F | ATACCTGTCCATCTGCCGGA |
| MVK-R | ATCCTCCTTGGTCCACCTGT |
| MVD-F | GTCAACATCGCGGTCATCAAG |
| MVD-R | TGTGGTGGTTTTTAACTGGTCC |
| FDPS-F | ATGCTATTGCCCGGCTCAAG |
| FDPS-R | GGAAGAAAGCTTGCAGCAGTT |
| FDFT1-F | CGGAAGGTGATGCCCAAGAT |
| FDFT1-R | ACACTGCGTTGCGCATTTC |
| LSS-F | CATGAAAATGCAGGGCACCA |
| LSS-R | ACCTGTGAGAGCCTCAGGAA |
| SC5D-F | GATGGAGAGACTTCAGCGCC |
| SC5D-R | ATCCATCACTTAGCCCCTGC |
| **The cDNA target sequences of shRNAs** | |
| shRPL6-1 sense | GGUGGUUAAACUUCGCAAATT |
| shRPL6-1 antisense | UUUGCGAAGUUUAACCACCTT |
| shRPL6-2 sense | GCUGGCUAGUGGCUUAUUATT |
| shRPL6-2 antisense | UAAUAAGCCACUAGCCAGCTT |
| shHMGCS1-1 sense | GAGACAAUCAUCGACAAAUTT |
| shHMGCS1-1 antisense | AUUUGUCGAUGAUUGUCUCTT |
| shHMGCS1-2 sense | GCAGGAGAUAUUGCUGUAUTT |
| shHMGCS1-2 antisense | AUACAGCAAUAUCUCCUGCTT |
| **Primer sequences used for RIP** | |
| GAPDH F | GCATTGCCCTCAACGACCAC |
| GAPDH R | CCACCACCCTGTTGCTGTAG |
| HMGSC1 F | AAGTCACACAAGATGCTACACCG |
| HMGSC1 R | TCAGCGAAGACATCTGGTGCCA |
| **Primer sequences used for gene/strand-specific PCR** | |
| RPL6(1-95) F | GCGGGTGAAAAAGTTGAGAA |
| RPL6(1-95) R | TGGTTTTGTAACAGTTGCGA |
| RPL6(96-136) F | CCAGTTGGTGGTGACAAGAA |
| RPL6(96-136) R | GTGCTGACTGAAGGGTTTTT |
| RPL6(137-288) F | CACGTGAGAAAACTGCGAGC |
| RPL6(137-288) R | GAACACCAATTTGTGAGGAT |
| RPL6(1-288) F | GCGGGTGAAAAAGTTGAGAA |
| RPL6(1-288) R | GAACACCAATTTGTGAGGAT |
| HMGCS1-CDS F | ATGCCTGGATCACTTCCTTT |
| HMGCS1-CDS R | TTAATGTTCCCCATTACTAA |
| HMGCS1-5'UTR F | GCGGACTGTCCTTTCGTGGC |
| HMGCS1-5'UTR R | GGTGAAAGAGCTGTGTGAAG |
| HMGCS1-3'UTR F | GATACTCTGTGAGGTGCAAG |
| HMGCS1-3'UTR R | TTGAATGAAAGTCATTTATT |
| HMGCS1-3'UTR (WT1) F | TTAAAAATTTAAAATGGAAGGGA |
| HMGCS1-3'UTR (WT1) R | TCCCTTCCATTTTAAATTTTTAA |
| HMGCS1-3'UTR (WT2) F | GACTTCATTTAACATTCACT |
| HMGCS1-3'UTR (WT2) R | GCACCCAAAAGTTAAATTAC |
| HMGCS1-3'UTR (WT3) F | CCATTCATTTATCATTTCAC |
| HMGCS1-3'UTR (WT3) R | TAATTATCAGTGTAAATTCC |
| HMGCS1-3'UTR (WT4) F | ATAAATATTTATGAAATGAG |
| HMGCS1-3'UTR (WT4) R | TACGTTCACCACTAAATTTG |
| HMGCS1-3UTR (WT5) F | GTCATAATTTACTTTCTGGG |
| HMGCS1-3UTR (WT5) R | TTAAAGCCATACTAAATAAT |
| HMGCS1-3UTR (WT6) F | TTAAATAATTTAAAGTTGTCTGAT |
| HMGCS1-3UTR (WT6) R | ATCAGACAACTTTAAATTATTTAA |
| **CRISPR gRNA sequences** | |
| RPL6 sgRNA F | GGTCCTCTTCCCTTATACCT |
| RPL6 sgRNA R | AGGTATAAGGGAAGAGGACC |
| Non-targeting sgRNA F | caccgACGGAGGCTAAGCGTCGCAA |
| Non-targeting sgRNA R | aaacTTGCGACGCTTAGCCTCCGTc |
| **Primers used for identification of RPL6 conditional knockout mice** | |
| flox-RPL6-P1 | TCTGAGCCACCTTCCCCATA |
| flox-RPL6-P2 | CTCTGGCACACTGGAGACAG |
| Flox-RPL6-P5 | GCGCTTTGTACCTTTGCCTC |
| Flox-RPL6-P6 | CATGCTCCTGCAATGCCAAG |
| Cre-WT F | TGCAAACATCACATGCACAC |
| Cre-Common R1 | TTGGCCCCTTACCATAACTG |
| Cre-Mutant R2 | GAAGCAGAAGCTTAGGAAGATGG |

**Supporting Table 5. The source data of isotopologue distribution of cholesterol in RPL6-knockout and overexpressing cells (%) in this study.**

| **Isotopologue distribution in RPL6-knockout cells (%)** | | | | | | | | |
| --- | --- | --- | --- | --- | --- | --- | --- | --- |
|  | Non-target control | | | | RPL6 KO | | | |
| M+0 | 76.7003 | 72.1522 | 77.3923 | 71.6159 | 91.4984 | 87.3031 | 86.1841 | 90.2090 |
| M+3 | 0.9564 | 1.2447 | 0.9142 | 1.2582 | 0.0000 | 0.0000 | 0.0000 | 0.0000 |
| M+4 | 1.1216 | 1.2703 | 0.9965 | 1.3143 | 0.0082 | 0.0054 | 0.0055 | 0.0208 |
| M+5 | 5.0947 | 5.4021 | 4.8535 | 5.4394 | 5.3568 | 5.2016 | 5.3252 | 4.5706 |
| M+6 | 1.1697 | 1.3687 | 1.2104 | 1.3576 | 0.4247 | 0.3923 | 0.4287 | 0.4114 |
| M+7 | 1.1726 | 1.4924 | 1.4302 | 1.6055 | 0.0296 | 0.0348 | 0.0282 | 0.0384 |
| M+8 | 1.4843 | 1.9584 | 1.5510 | 1.8731 | 0.0102 | 0.0085 | 0.0068 | 0.0092 |
| M+9 | 2.2204 | 2.6208 | 1.9228 | 2.6394 | 0.0098 | 0.0087 | 0.0101 | 0.0156 |
| M+10 | 2.5808 | 2.9057 | 2.0348 | 2.9199 | 0.0124 | 0.0195 | 0.0124 | 0.0283 |
| M+11 | 2.4314 | 2.7560 | 2.2563 | 2.7261 | 0.0138 | 0.0198 | 0.0166 | 0.0252 |
| M+12 | 2.5360 | 2.6347 | 2.1738 | 2.2434 | 0.0226 | 0.0261 | 0.0196 | 0.0317 |
| **Isotopologue distribution in RPL6-** **overexpressing cells (%)** | | | | | | | | |
|  | Vector | | | | RPL6 | | | |
| M+0 | 88.8363 | 88.8991 | 90.0081 | 91.3721 | 78.1017 | 81.5012 | 81.0915 | 80.0111 |
| M+3 | 0.0000 | 0.0000 | 0.0000 | 0.0000 | 1.6982 | 1.7809 | 1.5949 | 2.0714 |
| M+4 | 0.0000 | 0.0000 | 0.0000 | 0.0000 | 2.3196 | 2.3802 | 2.4578 | 2.4080 |
| M+5 | 5.8523 | 5.0283 | 5.3333 | 4.7214 | 4.7859 | 4.7718 | 4.9184 | 4.7219 |
| M+6 | 0.4221 | 0.4309 | 0.4456 | 0.4163 | 0.6176 | 0.6309 | 0.6027 | 0.6053 |
| M+7 | 0.0237 | 0.0265 | 0.0268 | 0.0193 | 0.4161 | 0.4332 | 0.4046 | 0.4287 |
| M+8 | 0.0020 | 0.0010 | 0.0024 | 0.0006 | 0.5732 | 0.5726 | 0.6056 | 0.5207 |
| M+9 | 0.0001 | 0.0000 | 0.0000 | 0.0000 | 0.8886 | 0.9255 | 0.8747 | 0.9488 |
| M+10 | 0.0002 | 0.0001 | 0.0000 | 0.0000 | 1.1801 | 1.4201 | 1.3305 | 1.3116 |
| M+11 | 0.0007 | 0.0001 | 0.0002 | 0.0005 | 1.4343 | 1.5168 | 1.6065 | 1.6930 |
| M+12 | 0.0002 | 0.0006 | 0.0002 | 0.0002 | 1.8643 | 1.7858 | 1.8142 | 2.1250 |
